# Application of heterocyclic aldehydes as components in Ugi–Smiles couplings

**DOI:** 10.3762/bjoc.12.191

**Published:** 2016-09-15

**Authors:** Katelynn M Mason, Michael S Meyers, Abbie M Fox, Sarah B Luesse

**Affiliations:** 1 Department of Chemistry, Southern Illinois University Edwardsville, Edwardsville, Illinois 62026, USA

**Keywords:** Diels–Alder cycloaddition, epoxyisoindoline, multicomponent coupling reaction, tandem reaction, Ugi–Smiles coupling

## Abstract

Efficient one-pot Ugi–Smiles couplings are reported for the use of furyl-substituted aldehyde components. In the presence of these heterocyclic aldehydes, reactions tolerated variations in amine components and led to either isolated *N*-arylamide Ugi–Smiles adducts or *N*-arylepoxyisoindolines, products of tandem Ugi–Smiles Diels–Alder cyclizations, in moderate yields. A thienyl-substituted aldehyde was also a competent component for Ugi–Smiles adduct formation.

## Introduction

Synthetic methods to efficiently prepare libraries of biologically-relevant compounds are in demand and have inspired the development of new multicomponent coupling reactions. Isocyanide-based multicomponent couplings [1], led by the foundational Ugi four-component coupling [2–3], have been used extensively for the synthesis of natural products and the preparation of diverse heterocyclic scaffolds. In 2005, El Kaïm and co-workers extended the utility of the Ugi reaction with the development of an Ugi–Smiles reaction, replacing the carboxylic acid component with an electron-deficient phenol [4–7].

Recent efforts to assemble biologically-relevant heterocycles have used multicomponent couplings in combination with post-condensation processes to efficiently increase structural complexity [8–10]. One of the most effective routes to polycyclic core structures uses intramolecular Diels–Alder reactions (IMDA) of tethered, substituted furans to provide stereoselective construction of nitrogen-containing heterocyclic systems [11–13]. Multicomponent coupling reactions (MCRs) have been combined with IMDA approaches to efficiently increase molecular complexity [14] and prepare complex molecular scaffolds for the synthesis of natural products [15–16]. While the Ugi–Smiles condensation has generally found success in cascade processes [17–19], the intolerance of heterocyclic aldehyde components has prevented use with common IMDA strategies.

**Scheme 1:**
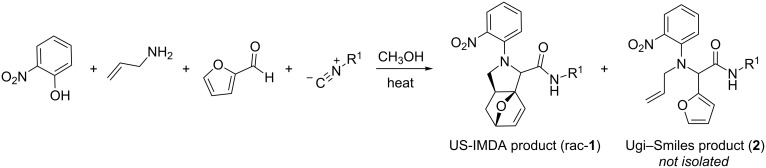
*N*-Arylepoxyisoindolines via tandem Ugi–Smiles/IMDA reaction.

Although heterocyclic aldehyde components are competent partners for the classic Ugi reaction [20], they have been inefficient carbonyl reactants for the four-component Ugi–Smiles coupling [21]. A notable exception is one reported example of 2-furaldehyde participating in a Ti(O-iPr)_4_-catalyzed modified Ugi–Smiles reaction that used an isocyanide as an amine equivalent [22]. We recently reported a successful tandem Ugi–Smiles intramolecular Diels–Alder (US-IMDA) reaction with substituted 2-furaldehyde and allylamine (Scheme 1), which provides direct access to *N*-arylepoxyisoindolines **1** through a simple, one-pot reaction [23].

Through this stereoselective tandem process, six new bonds and four stereocenters are generated in one synthetic step from achiral starting materials, producing two diastereomeric *exo* products that feature rigid tricyclic cores. Lone Ugi–Smiles adducts **2** were not isolated for any reactions that used a substituted 2-furaldehyde component. However, generation of adduct **1** can be rationalized as an Ugi–Smiles reaction, followed by cyclization, implying that furaldehyde derivatives can be competent components in lone Ugi–Smiles couplings. Herein we report our work with conjugated, heterocyclic aldehydes in the presence of various amine components to access novel heterocyclic building blocks through Ugi–Smiles couplings and tandem US-IMDA reactions.

## Results and Discussion

To extend the potential scaffolds accessible through Ugi–Smiles couplings with 2-furaldehyde, 2-methylallylamine was used in place of allylamine and resulted in the expected Ugi–Smiles IMDA products (Table 1).

**Table 1:** Tandem Ugi–Smiles/IMDA reactions with 2-furaldehyde.

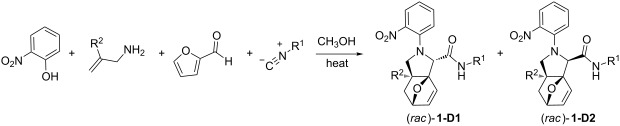					

Entry	R^1^ =	R^2^	Conditions	Products	Yield (%)^a^

1	*tert-*butyl	H	24 h, 60 °C	**1a-D1, 1a-D2**	52
2	cyclohexyl	H	24 h, 60 °C	**1b-D1, 1b-D2**	68
3	*tert-*butyl	CH_3_	30 h, 50 °C	**1c-D1, 1c-D2**	43^b^
4	cyclohexyl	CH_3_	30 h, 50 °C	**1d-D1, 1d-D2**	53^b,c^

^a^Standard reaction (0.5 mmol, 1.0 M) performed with 2.0 equiv isocyanide. Only *exo*-adducts observed. Both diastereomers at the α-amino amide carbon were observed in ≈1:1 ratio. Yields represent the sum of the two diastereomers obtained after products were separated via column chromatography; see Supporting Information File 1 for details. ^b^Diastereomers had same relative stereochemistry compared to analogous products **1a**,**b**, but a 1:2 diastereomeric ratio was observed. ^c^1.0 equiv isocyanide.

We examined a range of amine components to determine the substrate scope that would be tolerated in this tandem process (Table 2). Amines were combined with 2-nitrophenol, cyclohexyl or *tert*-butyl isocyanide, and 2-furaldehyde in methanol (50 °C, 30 h). As no lone Ugi–Smiles reactions had been reported with furyl-substituted aldehydes, initial studies evaluated only amines that included an available alkene for participation in a tandem US-IMDA reaction. However, these amine components provided access to uncyclized Ugi–Smiles adducts, demonstrating the first successful four-component Ugi–Smiles reactions with a furyl-substituted aldehyde. These results led to exploration of alkylamines, providing modest yields of Ugi–Smiles adducts. Propargylamine was not an effective amine for this reaction, providing no significant Ugi–Smiles or Ugi–Smiles-IMDA products with 2-furaldehyde.

**Table 2:** Ugi–Smiles couplings with 2-furaldehyde.

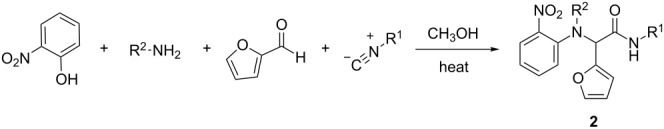				

Entry	R^1^ =	Amine (R^2^NH_2_)	Product	Yield (%)^a^

1	*tert-*butyl	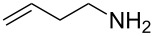	**2a**	40^b^
2	cyclohexyl	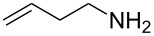	**2b**	34
3	cyclohexyl	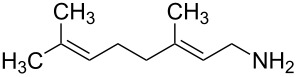	**2c**	35
4	cyclohexyl	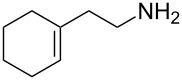	**2d**	35
5	cyclohexyl	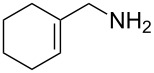	**2e**	36
6	cyclohexyl	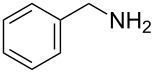	**2f**	28
7	cyclohexyl	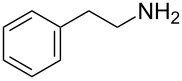	**2g**	28
8	cyclohexyl	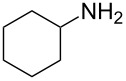	**2h**	25

^a^Standard reaction (0.5 mmol, 1.0 M, 50 °C, 30 h) performed with 1.0 equiv isocyanide. ^b^2.0 equiv isocyanide.

**Scheme 2:**
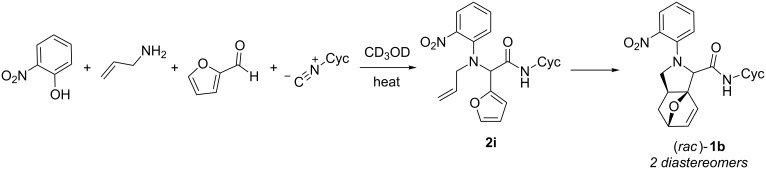
Reaction monitoring by ^1^H NMR for production of **1b**.

Observation of lone Ugi–Smiles products **2a**–**h** from use of 2-furaldehyde supported our understanding of the observed tandem US-IMDA reaction with allylamine as an Ugi–Smiles coupling followed by an intramolecular cyclization. We were interested in exploring the reaction pathway in an effort to improve reaction conversion. As Ugi–Smiles products had not been isolated from crude reaction mixtures for reactions with 2-furaldehyde and allylamine after 30 h, the cycloaddition step was assumed to be rapid compared to the Ugi–Smiles coupling. A standard reaction, with 2-furaldehyde and allylamine components to produce product **1b** in methanol*-d*_4_, was performed in a sealed NMR tube and monitored by ^1^H NMR to investigate the formation of Ugi–Smiles products prior to cyclization (Scheme 2). Conversion was determined by ^1^H NMR integration of product peaks relative to an aromatic peak of the starting material, 2-nitrophenol (see Supporting Information File 1 for ^1^H NMRs used in reaction monitoring).

After six hours, the reaction mixture contained ≈15% Ugi–Smiles adduct **2i** and ≈17% cyclized US-IMDA diastereomers **1b** (as determined by ^1^H NMR integration), with the remainder of the material present as unreacted starting material or imine, generated from 2-furaldehyde and allylamine. This crude reaction mixture was purified via column chromatography to provide an isolated sample of **2i** for characterization. Notably, product **2i** underwent almost complete Diels–Alder cycloaddition even without heating after 72 hours at 23 °C.

The use of 3-furaldehyde as a component resulted in standard Ugi–Smiles adducts **3** (Table 3). The lack of oxatricyclic epoxyisoindoline formation is not surprising, given the more remote relative proximity of the diene and dienophile. Propargylamine and 3-butenylamine were also satisfying partners with 3-furaldehyde in this process.

**Table 3:** Ugi–Smiles couplings with 3-furaldehyde.

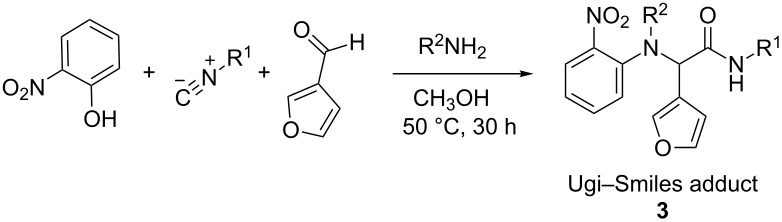				

Entry	R^1^	R^2^NH_2_	Product	Yield (%)^a^

1	*tert-*butyl	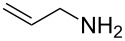	**3a**	45^b^
2	cyclohexyl	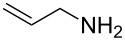	**3b**	64
3	*tert-*butyl	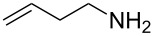	**3c**	58^b^
4	cyclohexyl	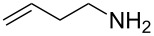	**3d**	52
5	*tert-*butyl	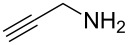	**3e**	23^b^
6	cyclohexyl	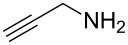	**3f**	48

^a^Standard reaction conditions (0.5 mmol, 1.0 M). ^b^2.0 equiv isocyanide.

Ugi–Smiles reactions with 3-furaldehyde were generally higher yielding than the analogous 2-furaldehyde examples. This difference in reactivity can be explained by the greater delocalization present for the 2-furaldehyde carbonyl system, making the carbonyl (and resulting imine intermediate) less susceptible to nucleophilic attack. Both competitive studies and side-by-side reactions, monitored by ^1^H NMR, showed that the formation of product **3b** from 3-furaldehyde is more rapid than the formation of uncyclized **2i** and cyclized **1b** from 2-furaldehyde. It is significant to note that there is never substantial accumulation of Ugi–Smiles product **2i** without observation of cyclized product **1b**.

For heterocyclic aldehydes, allylamine generally provided the most efficient amine coupling partner, but a range of simple amines were competent components in this reaction. Computational studies of substituent effects in the Ugi–Smiles reaction have indicated that both aryl-imidate formation and the final Smiles rearrangement are rate-determining steps [24]. Reactions using efficient amine components have relatively low activation energies for aryl-imidate formation and Smiles rearrangement [25] that may compensate for the higher barriers associated with the use of heterocyclic aldehydes, compared to simple aliphatic aldehydes, providing access to Ugi–Smiles adducts.

**Scheme 3:**
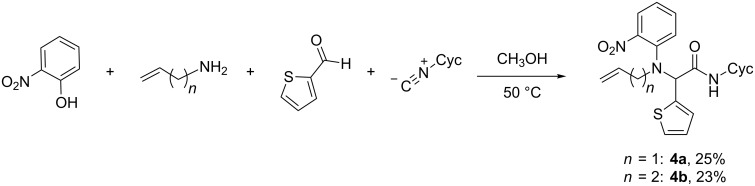
Use of a thienyl-substituted aldehyde for Ugi–Smiles couplings.

The use of a sulfur-based heterocyclic aldehyde, thiophene-2-carboxaldehyde, provided the Ugi–Smiles adducts in low yields (Scheme 3). While such thienyl-substituted aldehydes have been employed in standard Ugi reactions for the preparation of druglike heterocycles [26–28], *N*-arylamides **4a,b** represent the first examples of analogous Ugi–Smiles adducts incorporating a thienyl-substituted aldehyde component.

## Conclusion

In summary, Ugi–Smiles couplings have been observed for 2- and 3-furaldehyde with a variety of amine components. In the presence of a competent dienophile, the Ugi–Smiles coupling is followed by a facile intramolecular Diels–Alder cycloaddition to generate oxatricyclic *N*-arylepoxyisoindolines. Initial results with thiophene-2-carboxaldehyde show promise for the incorporation of other heterocyclic aldehydes in the Ugi–Smiles reaction. It is noteworthy that these examples expand the range of successful aldehyde components for Ugi–Smiles couplings, while providing direct access to heterocyclic *N*-arylamide adducts.

## Experimental

### General procedure for the synthesis of Ugi–Smiles or US-IMDA products

To a solution of 2-nitrophenol (69.5 mg, 0.5 mmol, 1 equiv) in methanol (0.50 mL) was added aldehyde (0.5 mmol, 1 equiv), amine (0.5 mmol, 1 equiv), and an isocyanide (0.5 mmol, 1 equiv). The reaction mixture was warmed at 50 °C for 30 h. Removal of volatiles gave the crude material, which was purified via flash column chromatography on silica gel. For full details, see Supporting Information File 1.

## Supporting Information

File 1 Experimental procedures and analytical data for Ugi–Smiles and US-IMDA products.
