# Supplementary material for: Application of heterocyclic aldehydes as components in Ugi–Smiles couplings
Source: Beilstein J Org Chem. 2016 Sep 15;12:2032–7. doi: 10.3762/bjoc.12.191 (PMC5082724; doi:10.3762/bjoc.12.191)
Supplement: File 1 — Experimental procedures and analytical data for Ugi–Smiles and US-IMDA products. [file Beilstein_J_Org_Chem-12-2032-s001.pdf]

Supporting Information

for

Application of heterocyclic aldehydes as components in Ugi–Smiles couplings

Katelynn M. Mason[1], Michael S. Meyers[1], Abbie M. Fox[1] and Sarah B. Luesse[1]*

Address: [1]Department of Chemistry, Southern Illinois University Edwardsville, Edwardsville, Illinois 62026, USA

Email: Sarah Luesse - sluesse@siue.edu

* Corresponding author

Experimental procedures and analytical data for Ugi–Smiles and US-IMDA products

Table of Contents

1. General experimental details S1

2. Synthesis of Ugi-Smiles adducts **1c-4b** S2

3. Stacked ^{1}H NMRs for reaction monitoring (related to Scheme 2) S13

4. ^{1}H and ^{13}C NMR spectra of new compounds S14

General experimental details

Methanol was distilled from CaH_2 under N_2 immediately before use. 2-Furaldehyde was distilled under reduced pressure (62 °C, 18 mm). Cyclohex-1-en-1-ylmethyl amine and 1-cyclohex-1-en-1-ylethyl amine were purchased as hydrochloride salts and converted to the free base form via 1.0 M NaOH prior to use. All other reagents and solvents were commercial grade (Aldrich or Acros) and used without purification. Characterization data for compounds **1a-D1**, **1a-D2**, **1b-D1**, and **1b-D2** have been reported previously [1]. Novel Ugi-Smiles and tandem US-IMDA products in this work were prepared in a similar manner to experimental procedures previously reported for known US-IMDA products [1].

1. Richey, B.; Mason, K. M.; Meyers, M. S.; Luesse, S. B. *Tetrahedron Lett*. **2016**, *57*, 492-494.

Thin layer chromatography (TLC) was performed using plastic-backed silica gel (225 µm) plates and flash chromatography utilized 230–400 mesh silica gel from Sigma-Aldrich. Some compounds were purified via automated chromatography on the Biotage Isolera™ Flash Purification system using a gradient method with SNAP Ultra cartridges. Products were visualized by UV light, and/or the use of ceric ammonium molybdate, *p*-anisaldehyde, and potassium iodoplatinate solutions.

IR spectra were recorded on a Nicolet™ iS5 FT-IR Spectrometer and are reported in wavenumbers (cm^{-1}). Liquids and oils were analyzed as neat films on a NaCl plate (transmission), whereas solids were applied to a diamond plate (ATR). Nuclear magnetic resonance spectra (NMR) were acquired on a Varian Unity Plus (300 MHz) or a Bruker Ascend (400 MHz) Spectrometer and processed with TopSpin 3.2. Chemical shifts are measured relative to residual solvent peaks as an internal standard set to δ 7.26 and δ 77.1 ($CDCl_3$). HRMS FAB data was collected from a JEOL MStation [JMS-700] Mass Spectrometer at the University of Missouri-St Louis. Direct infusion mass spectrometry (DIMS) was used to verify accurate masses for several compounds, using a Triversa Nanomate nanospray direct infusion robot (Advion) attached to a Q-Exactive Mass Spectrometer. These samples were analyzed at the Proteomics & Mass Spectrometry Facility at the Danforth Plant Science Center (St Louis, MO).

Synthesis of *N*-(*tert*-butyl)-7a-methyl-2-(2-nitrophenyl)-1,2,3,6,7,7a-hexahydro-3a,6-epoxyisoindole-3-carboxamide (1c).

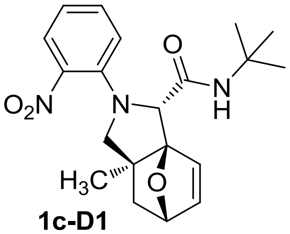

1c-D1

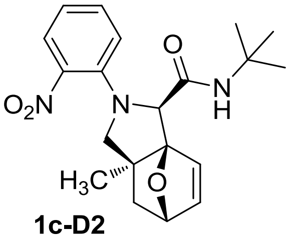

1c-D2

In a similar manner as described in [1], to a solution of 2-nitrophenol (69.6 mg, 0.5 mmol, 1 equiv) in methanol (0.50 mL) was added 2-furaldehyde (41.4 µL, 0.5 mmol, 1 equiv), 2-methylallylamine (45.6 µL, 0.5 mmol, 1 equiv), and *tert*-butyl isocyanide (114 µL, 1.0 mmol, 2 equiv). The reaction mixture was warmed at 50 °C for 30 h. Removal of volatiles gave crude material, which was purified via flash column chromatography on silica gel (10% ethyl acetate in hexanes) to afford epoxyisoindolines **1c-D1** (26.0 mg, 14%) and **1c-D2** (53.9 mg, 29%).

1c-D1: R_f = 0.40 (40:60 EtOAc:Hex); ^{1}H NMR (400 MHz, $CDCl_3$) δ 7.73 (m, 2H), 7.47 (dd, *J* = 8.2, 7.6 Hz, 1H), 7.32 (d, *J* = 8.5 Hz, 1H), 7.09 (dd, *J* = 7.8, 7.7 Hz, 1H), 6.56 (d, *J* = 5.9 Hz, 1H), 6.42 (d, *J* = 5.8 Hz, 1H), 4.97 (d, *J* = 5.0 Hz, 1H), 4.57 (s, 1H), 3.45 (d, *J* = 8.5 Hz, 1H), 2.98 (d, *J* = 8.2 Hz, 1H), 2.08 (dd, *J* = 4.9, 4.4 Hz, 1H), 1.32 (s, 9H), 1.28-1.23 (m, 1H), 1.08 (s, 3H); ^{13}C NMR (100 MHz, $CDCl_3$) δ 166.9, 142.3, 139.0, 137.7, 133.6, 132.5, 126.5, 118.9, 116.2, 96.9, 80.7, 65.4, 62.9, 51.4, 49.1, 38.6, 28.5, 22.6; DIMS $[M+H]^+$ calcd for $C_{20}H_{26}N_3O_4$ 372.1923; found 372.1916.

1c-D2: R_f = 0.29 (40:60 EtOAc:Hex); ^{1}H NMR (400 MHz, $CDCl_3$) δ 7.78 (d, *J* = 8.2 Hz, 1H), 7.42 (dd, *J* = 8.1, 7.8 Hz, 1H), 7.07 (d, *J* = 8.5 Hz, 1H), 6.90 (dd, *J* = 7.9, 7.6 Hz, 1H), 6.56 (d, *J* = 5.6 Hz, 1H), 6.48 (d, *J* = 5.7 Hz, 1H), 6.45 (s, 1H), 5.08 (s, 1H), 4.75 (s, 1H), 3.70 (d, *J* = 9.6 Hz, 1H), 2.63 (d, *J* = 9.6 Hz, 1H), 2.19 (dd, *J* = 4.7, 4.6 Hz, 1H), 1.19 (s, 9H), 1.05 (d, *J* = 11.6 Hz, 1H), 0.82 (s, 3H); ^{13}C NMR ($CDCl_3$) δ 168.2, 142.1, 135.7, 134.0, 133.1, 125.8, 122.8,

121.9, 98.5, 79.7, 65.1, 65.3, 51.3, 48.6, 39.8, 29.3, 28.6, 23.2; HR-FAB MS $[M+Na]^+$ calcd for $C_{20}H_{25}N_3NaO_4$ 394.1743; found 394.1746.

Synthesis of *N*-cyclohexyl-7a-methyl-2-(2-nitrophenyl)-1,2,3,6,7,7a-hexahydro-3a,6-epoxyisoindole-3-carboxamide (1d).

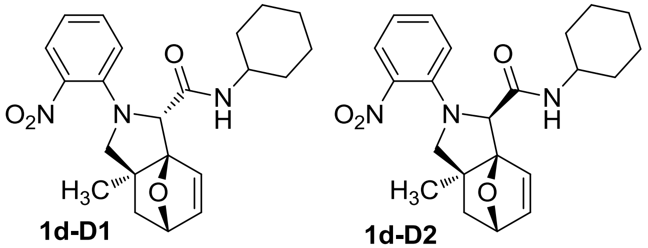

1d-D1 **1d-D2**

In a similar manner as described in [1], to a solution of 2-nitrophenol (69.8 mg, 0.50 mmol, 1 equiv) in methanol (0.50 mL) was added 2-furaldehyde (41.4 μL, 0.5 mmol, 1 equiv), 2-methylallylamine (45.6 μL, 0.5 mmol, 1 equiv), and cyclohexyl isocyanide (62.2 μL, 0.5 mmol, 1 equiv). The reaction mixture was warmed at 50 °C for 30 h. Removal of volatiles gave crude material, which was purified via flash column chromatography on silica gel (15% ethyl acetate in hexanes) to give epoxyisoindolines **1d-D1** (36.9 mg, 19%) and **1d-D2** (67.5 mg, 34%).

1d-D1: R_f = 0.35 (50:50 EtOAc:Hex); ^{1}H NMR (400 MHz, $CDCl_3$) δ 7.84 (d, *J* = 7.8 Hz, 1H, NH), 7.72 (dd, *J* = 8.1, 1.4 Hz, 1H), 7.46 (dd, *J* = 7.9, 7.7 Hz, 1H), 7.31 (d, *J* = 8.2 Hz, 1H), 7.09 (dd, *J* = 7.3, 7.3 Hz, 1H), 6.56 (d, *J* = 5.8 Hz, 1H), 6.43 (dd, *J* = 5.8, 1.6 Hz, 1H), 4.98 (dd, *J* = 4.6, 1.5 Hz, 1H), 4.70 (s, 1H), 3.81-3.69 (m, 1H), 3.43 (d, *J* = 8.4 Hz, 1H), 3.00 (d, *J* = 8.4 Hz, 1H), 2.08 (dd, *J* = 11.5, 4.8 Hz, 1H), 1.95-1.87 (m, 1H), 1.74-1.59 (m, 4H), 1.41-1.11 (m, 5H), 1.00 (s, 3H), 0.94-0.79 (m, 1H); ^{13}C NMR (100 MHz, $CDCl_3$) δ 167.8, 141.8, 135.6, 133.9, 132.9, 125.6, 122.8, 121.9, 98.2, 79.6, 65.3, 64.7, 48.6, 48.0, 39.6, 32.9, 32.5, 30.9, 25.3, 24.6(2), 23.0; HR-FAB MS $[M+Na]^+$ calcd for $C_{22}H_{27}N_3NaO_4$ 420.1899; found 420.1907.

1d-D2: R_f = 0.18 (50:50 EtOAc:Hex); ^{1}H NMR (400 MHz, $CDCl_3$) δ 7.78 (d, *J* = 8.2 Hz, 1H), 7.42 (dd, *J* = 7.8, 7.8 Hz, 1H), 7.07 (d, *J* = 8.4 Hz, 1H), 6.90 (dd, *J* = 7.4, 7.4 Hz, 1H), 6.59 (d, *J* = 5.7 Hz, 1H), 6.54 (s, 1H), 6.50 (d, *J* = 5.7 Hz, 1H), 5.08 (s, 1H), 4.87 (s, 1H), 3.73-3.70 (m, 2H), 2.65 (d, *J* = 9.6 Hz, 1H), 2.23-2.14 (m, 1H), 1.85-1.76 (m, 1H), 1.65-1.49 (m, 2H), 1.49-1.35 (m, 2H), 1.35-1.00 (m, 5H), 0.94-0.78 (m, 4H); ^{13}C NMR (100 MHz, $CDCl_3$) δ 166.6, 142.2, 139.0, 137.8, 133.6, 132.4, 126.5, 118.9, 116.2, 96.9, 80.7, 65.3, 62.6, 49.0, 47.8, 38.6, 32.5, 32.5, 25.5, 24.3, 24.2, 22.5; HR-FAB MS $[M+Na]^+$ calcd for $C_{22}H_{27}N_3NaO_4$ 420.1899; found 420.1907.

Synthesis of 2-(but-3-en-1-yl(2-nitrophenyl)amino)-*N*-(*tert*-butyl)-2-(furan-2-yl)acetamide (2a).

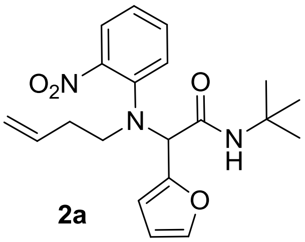

2a

In a similar manner as described in [1], to a solution of 2-nitrophenol (69.6 mg, 0.5 mmol, 1 equiv) in methanol (0.50 mL) was added 2-furaldehyde (41.4 μL, 0.5 mmol, 1 equiv), 3-

butenylamine (45.8 μL, 0.5 mmol, 1 equiv), and *tert*-butyl isocyanide (114 μL, 1.0 mmol, 2 equiv). The reaction mixture was warmed at 50 °C for 30 h. Removal of volatiles gave crude material, which was purified via flash column chromatography on silica gel (10% ethyl acetate/hexanes) to afford *N*-arylamide **2a** (71.1 mg, 40%). R_f = 0.20 (20:80 EtOAc:Hex); ^{1}H NMR (400 MHz, $CDCl_3$) δ 7.73 (d, *J* = 8.1 Hz, 1H), 7.49 (dd, *J* = 7.8, 7.7 Hz, 1H), 7.40 (s, 1H), 7.34 (br s, 1H, NH), 7.25-7.18 (m, 2H), 6.36-6.28 (m, 2H), 5.69-5.52 (m, 1H), 4.97-4.82 (m, 3H)¸ 3.03 (dd, *J* = 7.7, 7.0 Hz, 2H), 2.06-1.82 (m, 2H), 1.38 (s, 9H); ^{13}C NMR (100 MHz, $CDCl_3$) δ 167.3, 149.4, 147.5, 142.8, 142.1, 135.0, 133.0, 126.9, 125.4, 125.1, 117.2, 111.4, 110.5, 66.6, 51.4, 50.8, 32.0, 28.7; HR-FAB MS $[M+Na]^+$ calcd for $C_{20}H_{25}N_3NaO_4$ 394.1743; found 394.1748.

Synthesis of 2-(but-3-en-1-yl(2-nitrophenyl)amino)-*N*-cyclohexyl-2-(furan-2-yl)acetamide (2b).

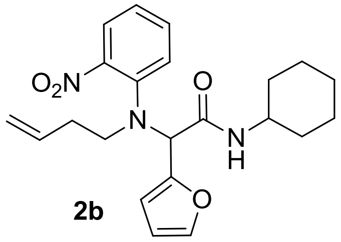

2b

In a similar manner as described in [1], to a solution of 2-nitrophenol (69.6 mg, 0.5 mmol, 1 equiv) in methanol (0.50 mL) was added 2-furaldehyde (41.5 μL, 0.5 mmol, 1 equiv), 3-butenylamine (45.8 μL, 0.5 mmol, 1 equiv), and cyclohexyl isocyanide (62.2 μL, 0.5 mmol, 1 equiv). The reaction mixture was warmed at 50 °C for 30 h. Removal of volatiles gave crude material, which was purified via flash column chromatography on silica gel (20% ethyl acetate in hexanes) to afford *N*-arylamide **2b** (68.2 mg, 34%). R_f = 0.51 (50:50 EtOAc:Hex); ^{1}H NMR (400 MHz, $CDCl_3$) δ 7.70 (d, *J* = 6.9 Hz, 1H), 7.50-7.41 (m, 2H), 7.36 (s, 1H), 7.28 (d, *J* = 7.3 Hz, 1H), 7.22 (dd, *J* = 7.4, 7.3 Hz, 1H), 6.32 (s, 2H), 5.59-5.48 (m, 1H), 5.02 (s, 1H), 4.92 (d, *J* = 10.1 Hz, 1H), 4.84 (d, *J* = 16.1 Hz, 1H), 3.80-3.73 (m, 1H), 3.06-2.93 (m, 2H), 1.96-1.84 (m, 3H), 1.80-1.67 (m, 3H), 1.59 (d, *J* = 12.5 Hz, 1H), 1.40-1.11 (m, 5H); ^{13}C NMR (100 MHz, $CDCl_3$) δ 167.0, 149.1, 147.2, 142.6, 142.0, 134.7, 132.9, 126.6, 125.1 124.9, 117.1, 111.2, 110.4, 65.7, 50.7, 48.3, 32.9, 32.5, 31.7, 25.4, 24.74, 24.67; HR-FAB MS $[M+H]^+$ calcd for $C_{22}H_{28}N_3O_4$ 398.2080; found 398.2068.

Synthesis of (E)-*N*-cyclohexyl-2-((3,7-dimethylocta-2,6-dien-1-yl)(2-nitrophenyl)amino)-2-(furan-2-yl)acetamide (2c).

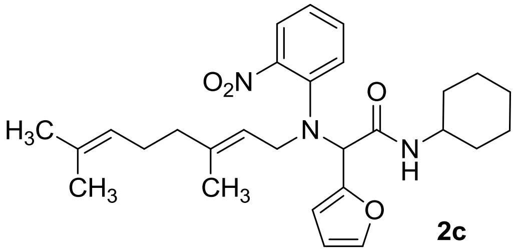

2c

In a similar manner as described in [1], to a solution of 2-nitrophenol (69.6 mg, 0.5 mmol, 1 equiv) in methanol (0.50 mL) was added 2-furaldehyde (41.5 μL, 0.5 mmol, 1 equiv), geranylamine (92.4 μL, 0.5 mmol, 1 equiv), and cyclohexyl isocyanide (62.2 μL, 0.5 mmol, 1 equiv). The reaction mixture was warmed at 50 °C for 30 h. Removal of volatiles gave crude material, which was purified via flash column on silica gel (40% ethyl acetate in hexanes) to afford *N*-arylamide **2c** (83.2 mg, 35%). R_f = 0.16 (40:60 EtOAc:Hex); ^{1}H NMR (400 MHz, $CDCl_3$) δ 7.66 (d, *J* = 8.0 Hz, 1H), 7.51 (d, *J* = 8.2 Hz, 1H), 7.44 (dd, *J* = 8.2, 8.1 Hz, 1H), 7.35-7.31 (m, 2H), 7.17 (dd, *J* = 7.3, 7.2 Hz, 1H), 6.40 (d, *J* = 2.9 Hz, 1H), 6.30 (d, *J* = 3.0 Hz, 1H), 5.06 (s, 1H), 4.95 (dd, *J* = 6.9, 6.8 Hz, 1H), 4.87 (dd, *J* = 6.8, 6.7 Hz, 1H), 3.73-3.66 (m, 1H), 3.55 (dd, *J* = 14.9, 7.6 Hz, 1H), 3.44 (dd, *J* = 14.8, 6.8 Hz, 1H), 1.91-1.84 (m, 5H), 1.72-1.51 (m, 10H), 1.37-1.13 (m, 7H), 1.12-1.01 (m, 1H); ^{13}C NMR (100 MHz, $CDCl_3$) δ 167.3, 149.6, 147.0, 142.7, 142.5, 141.3, 132.8, 131.7, 126.6, 124.74, 124.73, 123.9, 117.9, 110.9, 110.6, 64.8, 50.2, 48.2, 39.7, 32.8, 32.6, 26.3, 25.7, 25.5, 24.8, 24.7, 17.7, 15.9; HR-FAB MS $[M+Na]^+$ calcd for $C_{28}H_{37}N_3NaO_4$ 502.2682; found 502.2684.

Synthesis of 2-((2-(cyclohex-1-en-1-yl)ethyl)(2-nitrophenyl)amino)-*N*-cyclohexyl-2-(furan-2-yl)acetamide (2d).

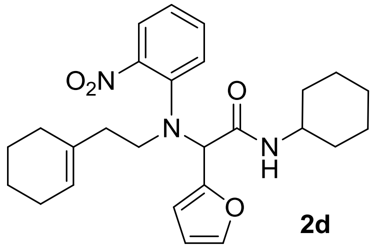

2d

In a similar manner as described in [1], to a solution of 2-nitrophenol (69.6 mg, 0.5 mmol, 1 equiv) in methanol (0.50 mL) was added 2-furaldehyde (41.5 μL, 0.5 mmol, 1 equiv), 2-(1-cyclohexenyl)ethylamine (69.7 μL, 0.5 mmol, 1 equiv), and cyclohexyl isocyanide (62.2 μL, 0.5 mmol, 1 equiv). The reaction mixture was warmed at 50 °C for 30 h. Removal of volatiles gave crude material, which was purified via flash column chromatography on silica gel (40% ethyl acetate in hexanes) to afford *N*-arylamide **2d** (85.8 mg, 35%). R_f = 0.13 (40:60 EtOAc:Hex); ^{1}H NMR (400 MHz, $CDCl_3$) δ 7.69 (d, *J* = 8.1 Hz, 1H), 7.49-7.45 (m, 2H), 7.37 (s, 1H), 7.33 (d, *J* = 7.7 Hz, 1H), 7.19 (dd, *J* = 7.3, 7.3 Hz, 1H), 6.38 (d, *J* = 2.5 Hz, 1H), 6.35-6.31 (m, 1H), 5.16 (s, 1H), 5.09 (s, 1H), 3.78-3.70 (m, 1H), 3.04-2.88 (m, 2H), 1.95-1.88 (m, 1H), 1.86-1.80 (m, 2H), 1.77-1.56 (m, 8H), 1.45-1.37 (m, 4H), 1.33-1.06 (m, 5H); ^{13}C NMR (100 MHz, $CDCl_3$) δ 167.3, 149.3, 146.6, 142.7, 142.5, 134.3, 133.0, 126.4, 125.0, 124.7, 123.4, 111.3, 110.6, 65.2, 50.4, 48.3, 35.9, 32.9, 32.6, 28.1, 25.5, 25.2, 24.8, 24.7, 22.8, 22.2; HR-FAB MS $[M+H]^+$ calcd for $C_{26}H_{34}N_3O_4$ 452.2544; found 452.2560.

Synthesis of 2-((cyclohex-1-en-1-ylmethyl)(2-nitrophenyl)amino)-*N*-cyclohexyl-2-(furan-2-yl)acetamide (2e).

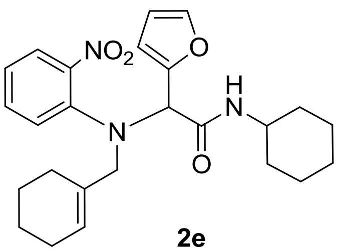

2e

Cyclohex-1-en-1-ylmethyl amine hydrochloride (95.3 mg) was treated with aqueous NaOH (1.0 M, 1 equiv), extracted with diethyl ether, and concentrated *in vacuo* to afford the free cyclohex-1-en-1-ylmethyl) amine (74.0 mg). In a similar manner as described in [1], to a solution of 2-nitrophenol (93.2 mg, 0.67 mmol, 1 equiv) in methanol (0.67 mL) was added 2-furaldehyde (55.5 μL, 0.67 mmol, 1 equiv), cyclohex-1-en-1-ylmethyl amine (74.0 mg, 0.67 mmol, 1 equiv), and cyclohexyl isocyanide (83.5 μL, 0.67 mmol, 1 equiv). The reaction mixture was warmed at 50 °C for 30 h. Removal of volatiles gave crude material, which was purified via flash column chromatography on silica gel (20% ethyl acetate in hexanes) to afford *N*-arylamide **2e** (105.1 mg, 36%). R_f = 0.55 (50:50 EtOAc:Hex); ^{1}H NMR (400 MHz, $CDCl_3$) δ 7.73 (d, *J* = 6.9 Hz, 1H), 7.52 (d, *J* = 8.1 Hz, 1H), 7.46 (dd, *J* = 8.4, 8.4 Hz, 1H), 7.37-7.33 (m, 2H), 7.13 (dd, *J* = 7.3, 7.3 Hz, 1H), 6.40 (d, *J* = 2.9 Hz, 1H), 6.34-6.31 (m, 1H), 5.28 (s, 1H), 5.16 (s, 1H), 3.81-3.73 (m, 1H), 3.50 (d, *J* = 14.2 Hz, 1H), 3.44 (d, *J* = 14.2 Hz, 1H), 1.97-1.90 (m, 1H), 1.78-1.58 (m, 6H), 1.52-1.49 (m, 2H), 1.42-1.24 (m, 7H), 1.20-1.11 (m, 2H); ^{13}C NMR (100 MHz, $CDCl_3$) δ 166.9, 149.5, 145.5, 142.6, 142.4, 133.1, 132.4, 128.4, 126.1, 125.2, 123.8, 111.3, 110.5, 66.0, 57.2, 48.4, 33.0, 32.6, 26.5, 25.5, 25.1, 24.80, 24.78. 22.4, 21.9; DIMS $[M+H]^+$ calcd for $C_{25}H_{32}N_3O_4$ 438.2393; found 438.2387.

Synthesis of 2-(benzyl(2-nitrophenyl)amino)-*N*-cyclohexyl-2-(furan-2-yl)acetamide (2f).

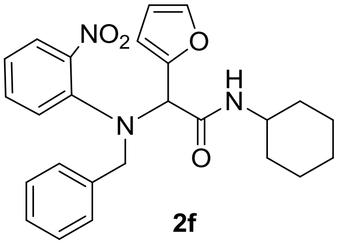

2f

In a similar manner as described in [1], to a solution of 2-nitrophenol (69.6 mg, 0.5 mmol, 1 equiv) in methanol (0.50 mL) was added 2-furaldehyde (41.5 μL, 0.5 mmol, 1 equiv), benzylamine (54.6 μL, 0.5 mmol, 1 equiv), and cyclohexyl isocyanide (62.2 μL, 0.5 mmol, 1 equiv). The reaction mixture was warmed at 50 °C for 30 h. Removal of volatiles gave crude material, which was purified via flash column chromatography (gradient 10-40% ethyl acetate in hexanes, 36 mL/min, Biotage Isolera, SNAP-Ultra cartridge) to afford *N*-arylamide **2f** (60.9 mg, 28%). R_f = 0.62 (50:50 EtOAc:Hex); ^{1}H NMR (400 MHz, $CDCl_3$) δ 7.62 (d, *J* = 8.0 Hz, 1H), 7.44 (br s, 1H, NH), 7.40-7.33 (m, 2H), 7.19-7.14 (m, 3H), 7.13-7.09 (m, 2H), 6.92-6.87 (m, 2H), 6.40 (d, *J* = 2.8 Hz, 1H), 6.35-6.32 (m, 1H), 5.06 (s, 1H), 4.20-4.06 (m, 2H), 3.80-3.69 (m, 1H), 1.93-1.84 (m, 1H), 1.77-1.64 (m, 3H), 1.63-1.58 (m, 1H), 1.36-1.28 (m, 2H), 1.27-1.21 (m, 2H), 1.18-1.10 (m, 1H); ^{13}C NMR (100 MHz, $CDCl_3$) δ 167.1, 149.3, 146.9, 142.9, 142.0, 135.6, 132.8, 129.0, 129.0, 128.4, 128.4, 127.7, 127.0, 125.1, 125.0, 111.4, 110.7, 65.7, 55.9, 48.4, 32.9, 32.7, 25.6, 24.9, 24.8; DIMS $[M+H]^+$ calcd for $C_{25}H_{28}N_3O_4$ 434.2080; found 434.2075.

Synthesis of *N*-cyclohexyl-2-(furan-2-yl)-2-((2-nitrophenyl)(phenethyl)amino)acetamide (2g).

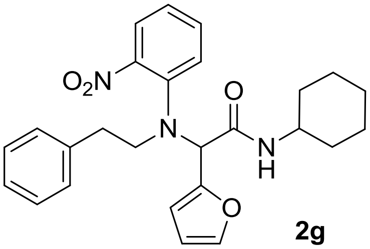

2g

In a similar manner as described in [1], to a solution of 2-nitrophenol (69.6 mg, 0.5 mmol, 1 equiv) in methanol (0.50 mL) was added 2-furaldehyde (41.5 µL, 0.5 mmol, 1 equiv), phenethylamine (62.9 µL, 0.5 mmol, 1 equiv), and cyclohexyl isocyanide (62.2 µL, 0.5 mmol, 1 equiv). The reaction mixture was warmed at 50 °C for 30 h. Removal of volatiles gave crude material, which was purified via flash column chromatography (gradient 13-40% ethyl acetate in hexanes, 36 mL/min, Biotage Isolera, SNAP-Ultra cartridge) to afford *N*-arylamide **2g** (62.7 mg, 28%). R_f = 0.55 (50:50 EtOAc:Hex); ^{1}H NMR (400 MHz, $CDCl_3$) δ 7.74 (d, *J* = 8.0 Hz, 1H), 7.52 (dd, *J* = 7.8, 7.8 Hz, 1H), 7.43-7.37 (m, 2H), 7.34 (br s, 1H, NH), 7.25-7.14 (m, 4H), 6.89 (d, *J* = 6.8 Hz, 2H), 6.40 (dd, *J* = 11.3, 2.2 Hz, 2H), 5.14 (s, 1H), 3.78-3.67 (m, 1H), 3.21-3.04 (m, 2H), 2.46-2.32 (m, 2H), 1.93-1.83 (m, 1H), 1.76-1.66 (m, 3H), 1.64-1.56 (m, 1H), 1.37-1.23 (m, 2H), 1.22-1.13 (m, 2H), 1.10-0.98 (m, 1H); ^{13}C NMR (100 MHz, $CDCl_3$) δ 167.0, 149.3, 146.7, 142.8, 142.2, 138.5, 133.2, 128.7, 128.6, 126.5, 126.2, 125.3, 125.0, 111.5, 110.7, 65.4, 53.0, 48.4, 33.9, 32.9, 32.5, 25.5, 24.9(2C); DIMS $[M+H]^+$ calcd for $C_{26}H_{30}N_3O_4$ 448.2236; found 448.2232.

Synthesis of *N*-cyclohexyl-2-(cyclohexyl(2-nitrophenyl)amino)-2-(furan-2-yl)acetamide (2h).

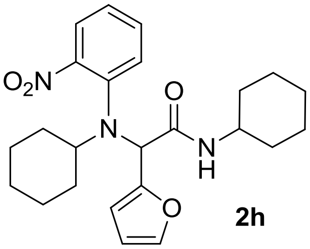

2h

In a similar manner as described in [1], to a solution of 2-nitrophenol (69.6 mg, 0.5 mmol, 1 equiv) in methanol (0.50 mL) was added 2-furaldehyde (41.5 µL, 0.5 mmol, 1 equiv), cyclohexylamine (57.3 µL, 0.5 mmol, 1 equiv), and cyclohexyl isocyanide (62.2 µL, 0.5 mmol, 1 equiv). The reaction mixture was warmed at 50 °C for 30 h. Removal of volatiles gave crude material, which was purified via flash column chromatography (gradient 12-40% ethyl acetate in hexanes, 36 mL/min, Biotage Isolera, SNAP-Ultra cartridge) to afford *N*-arylamide **2h** (53.2 mg, 25%). R_f = 0.57 (50:50 EtOAc:Hex); ^{1}H NMR (400 MHz, $CDCl_3$) δ 7.82 (br s, 1H, NH), 7.60 (d, *J* = 8.0 Hz, 1H), 7.37 (dd, *J* = 7.4, 7.4 Hz, 1H), 7.25-7.17 (m, 3H), 6.33 (d, *J* = 2.9 Hz, 1H), 6.18-6.14 (m, 1H), 5.06 (s, 1H), 3.77-3.65 (m, 1H), 2.80-2.70 (m, 1H), 1.91-1.83 (m, 2H), 1.77-1.62 (m, 6H), 1.61-1.54 (m, 1H), 1.52-1.45 (m, 1H), 1.36-1.18 (m, 4H), 1.16-1.05 (m, 3H), 0.96-0.74 (m, 3H); ^{13}C NMR (100 MHz, $CDCl_3$) δ 168.9, 150.1, 150.0, 142.3, 139.4, 131.8, 131.2, 126.4, 124.3, 110.8, 110.3, 64.8, 63.0, 48.1, 32.8, 32.7, 30.6, 29.2, 26.1, 25.9, 25.5, 24.9; DIMS $[M+H]^+$ calcd for $C_{24}H_{32}N_3O_4$ 426.2393; found 426.2388.

Synthesis of 2-(allyl(2-nitrophenyl)amino)-*N*-cyclohexyl-2-(furan-2-yl)acetamide (2i).

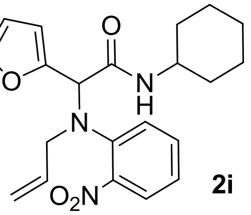

2i

In a similar manner as described in [1], to a solution of 2-nitrophenol (69.5 mg, 0.5 mmol, 1 equiv) in methanol (0.50 mL) was added 2-furaldehyde (41 µL, 0.5 mmol, 1 equiv), allyl amine (38 µL, 0.5 mmol, 1 equiv), and cyclohexyl isocyanide (62.2 µL, 0.5 mmol, 1 equiv). The reaction mixture was warmed at 50 °C for 6 h. Removal of volatiles gave the crude material, which was purified via flash column chromatography on silica gel (35% ethyl acetate in hexanes) to afford **2i** for characterization. R_f = 0.10 (40:60 Et_2O:Hex); ^{1}H NMR (400 MHz, $CDCl_3$) δ 7.70 (d, *J* = 8.1 Hz, 1H), 7.48 (dd, *J* = 7.8, 7.8 Hz, 1H), 7.38 (d, *J* = 1.8 Hz, 1H), 7.35 (d, *J* = 8.2 Hz, 1H), 7.19 (dd, *J* = 7.8, 7.7 Hz, 1H), 6.42 (d, *J* = 3.2 Hz, 1H), 6.33 (dd, *J* = 3.3, 1.4 Hz, 1H), 5.54-5.44 (m, 1H), 5.10 (s, 1H), 4.99 (d, *J* = 10.2 Hz, 1H), 4.92 (d, *J* = 17.1 Hz, 1H), 3.79-3.69 (m, 1H), 3.59-3.49 (m, 2H), 1.92-1.88 (m, 1H), 1.74-157 (m, 5H), 1.38-1.07 (m, 4H); ^{13}C NMR (100 MHz, $CDCl_3$) δ 166.9, 149.2, 142.7, 142.1, 133.0, 132.2, 126.3, 125.0, 124.7, 119.5, 111.2, 110.6, 77.2, 65.0, 54.5, 48.2, 32.9, 32.5, 25.5, 24.8, 24.7.

Synthesis of 2-(allyl(2-nitrophenyl)amino)-*N*-(*tert*-butyl)-2-(furan-3-yl)acetamide (3a).

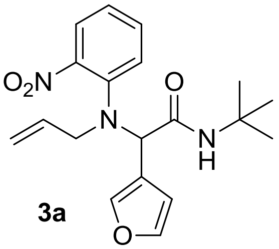

3a

In a similar manner as described in [1], to a solution of 2-nitrophenol (50.1 mg, 0.37 mmol, 1 equiv) in methanol (0.37 mL) was added 3-furaldehyde (31.2 µL, 0.37 mmol, 1 equiv), allylamine (27 µL, 0.37 mmol, 1 equiv), and *tert*-butyl isocyanide (81.5 µL, 0.74 mmol, 2 equiv). The reaction mixture was warmed at 50 °C for 30 h. Removal of volatiles gave crude material, which was purified via flash column chromatography on silica gel (20% ethyl acetate in hexanes) to afford *N*-arylamide **3a** (79.9 mg, 45%). R_f = 0.76 (50:50 EtOAc:Hex); ^{1}H NMR (400 MHz, $CDCl_3$) δ 7.67 (d, *J* = 7.4 Hz, 1H), 7.47-7.39 (m, 2H), 7.33 (s, 1H), 7.24-7.15 (m, 3H), 6.35 (s, 1H), 5.66-5.53 (m, 1H), 5.04 (d, *J* = 10.1 Hz, 1H), 4.95-4.89 (m, 1H), 4.76 (s, 1H), 3.58 (dd, *J* = 6.7, 6.6 Hz, 1H), 3.47 (dd, *J* = 6.5, 6.5 Hz, 1H), 1.25 (s, 9H); ^{13}C NMR (100 MHz, $CDCl_3$) δ 169.1, 147.2, 143.4, 142.2, 142.1, 132.7, 131.9, 126.6, 125.1, 124.8, 120.4, 120.0, 110.1, 64.0, 55.1, 51.1, 28.5; HR-FAB MS $[M+H]^+$ calcd for $C_{19}H_{24}N_3O_4$ 358.1767; found 358.1767.

Synthesis of 2-(allyl(2-nitrophenyl)amino)-*N*-cyclohexyl-2-(furan-3-yl)acetamide (3b).

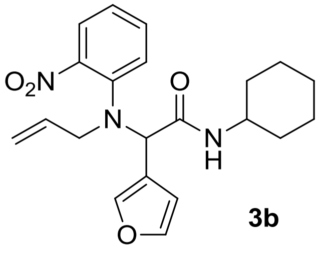

3b

TIn a similar manner as described in [1], to a solution of 2-nitrophenol (69.6 mg, 0.5 mmol, 1 equiv) in methanol (0.50 mL) was added 3-furaldehyde (43.3 μL, 0.5 mmol, 1 equiv), allylamine (38.0 μL, 0.5 mmol, 1 equiv), and cyclohexyl isocyanide (62.2 μL, 0.5 mmol, 1 equiv). The reaction mixture warmed at 50 °C for 30 h. Removal of volatiles gave crude material, which was purified via flash column chromatography on silica gel (15% ethyl acetate in hexanes) to afford *N*-arylamide **3b** (123.8 mg, 64%). R_f = 0.51 (50:50 EtOAc:Hex); ^{1}H NMR (400 MHz, $CDCl_3$) δ 7.75 (d, *J* = 8.0 Hz, 1H), 7.53-7.50 (m, 2H), 7.41 (s, 1H), 7.34-7.28 (m, 3H), 6.44 (s, 1H), 5.72-5.62 (m, 1H), 5.13 (d, *J* = 10.1 Hz, 1H), 5.02 (d, *J* = 17.1 Hz, 1H), 4.96 (s, 1H), 3.79-3.71 (m, 1H), 3.65 (dd, *J* = 6.7, 6.6 Hz, 1H), 3.55 (dd, *J* = 6.6, 6.5 Hz, 1H), 1.94-1.86 (m, 1H), 1.78-1.62 (m, 4H), 1.44-1.19 (m, 4H), 1.15-1.03 (m, 1H); ^{13}C NMR (100 MHz, $CDCl_3$) δ 168.8, 147.1, 143.3, 142.1, 141.8, 132.6, 131.6, 126.3, 125.0, 124.6, 120.3, 120.0, 109.9, 63.0, 55.1, 47.9, 32.7, 32.5, 25.4, 24.6, 24.5; HR-FAB MS $[M+Na]^+$ calcd for $C_{21}H_{25}N_3NaO_4$ 406.1743; found 406.1743.

Synthesis of 2-(but-3-en-1-yl(2-nitrophenyl)amino)-*N*-(*tert*-butyl)-2-(furan-3-yl)acetamide (3c).

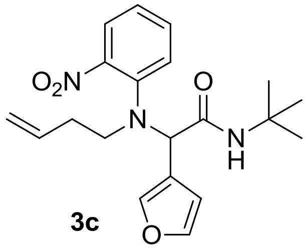

3c

In a similar manner as described in [1], to a solution of 2-nitrophenol (69.6 mg, 0.5 mmol, 1 equiv) in methanol (0.50 mL) was added 3-furaldehyde (43.2 μL, 0.5 mmol, 1 equiv), 3-butenylamine (45.8 μL, 0.5 mmol, 1 equiv), and *tert*-butyl isocyanide (113.1 μL, 1.0 mmol, 2 equiv). The reaction mixture was warmed at 50 °C for 30 h. Removal of volatiles gave crude material, which was purified via flash column chromatography on silica gel (15% ethyl acetate in hexanes) to afford *N*-arylamide **3c** (108.1 mg, 58%). R_f = 0.18 (20:80 EtOAc:Hex); ^{1}H NMR (400 MHz, $CDCl_3$) δ 7.64 (d, *J* = 7.9 Hz, 1H), 7.40 (dd, *J* = 7.4, 7.4 Hz, 1H), 7.30-7.23 (m, 2H), 7.23-7.08 (m, 3H), 6.28 (s, 1H), 5.61-5.46 (m, 1H), 4.92-4.79 (m, 2H), 4.67 (s, 1H), 3.06-2.94 (m, 1H), 2.91-2.79 (m, 1H), 2.03-1.81 (m, 2H), 1.24 (s, 9H); ^{13}C NMR (100 MHz, $CDCl_3$) δ 169.1, 147.9, 143.2, 142.3, 141.9, 135.0, 132.7, 126.9, 125.6, 124.9, 119.5, 117.2, 110.6, 64.8, 51.2, 51.1, 31.3, 29.6; HR-FAB MS $[M+H]^+$ calcd for $C_{20}H_{26}N_3O_4$ 372.1923; found 372.1911.

Synthesis of 2-(but-3-en-1-yl(2-nitrophenyl)amino)-*N*-cyclohexyl-2-(furan-3-yl)acetamide (3d).

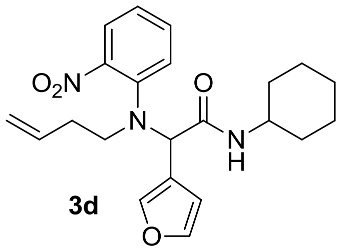

3d

In a similar manner as described in [1], to a solution of 2-nitrophenol (69.6 mg, 0.5 mmol, 1 equiv) in methanol (0.50 mL) was added 3-furaldehyde (43.3 µL, 0.5 mmol, 1 equiv), 3-butenylamine (45.8 µL, 0.5 mmol, 1 equiv), and cyclohexyl isocyanide (62.2 µL, 0.5 mmol, 1 equiv). The reaction mixture was warmed at 50 °C for 30 h. Removal of volatiles gave crude material, which was purified via flash column chromatography on silica gel (15% ethyl acetate in hexanes) to afford *N*-arylamide **3d** (103.3 mg, 52%). R_f = 0.51 (50:50 EtOAc:Hex); ^{1}H NMR (400 MHz, $CDCl_3$) δ 7.69 (d, *J* = 7.8 Hz, 1H), 7.47 (dd, *J* = 7.4, 7.4 Hz, 1H), 7.36 (s, 1H), 7.33 (s, 1H), 7.28-7.19 (m, 3H), 6.33 (s, 1H), 5.62-5.51 (m, 1H), 4.95-4.84 (m, 3H), 3.74-3.67 (m, 1H), 3.08-3.01 (m, 1H), 2.94-2.87 (m, 1H), 2.05-1.91 (m, 2H), 1.86 (d, *J* = 12.0 Hz, 1H), 1.75-1.54 (m, 5H), 1.38-1.12 (m, 3H), 1.09-1.00 (m, 1H); ^{13}C NMR (100 MHz, $CDCl_3$) δ 168.8, 147.7, 143.1, 142.2, 141.8, 134.7, 132.7, 126.6, 125.4, 124.8, 119.5, 117.1, 110.3, 63.9, 51.1, 48.0, 32.8, 32.5, 31.0, 25.4, 24.7, 24.6; HR-FAB MS $[M+Na]^+$ calcd for $C_{22}H_{27}N_3NaO_4$ 420.1899; found 420.1918.

Synthesis of *N*-(*tert*-butyl)-2-(furan-3-yl)-2-((2-nitrophenyl)-(prop-2-yn-1-yl)amino) acetamide (3e).

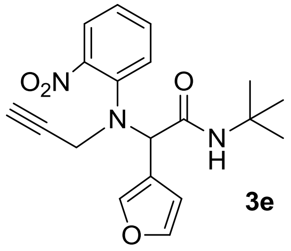

3e

In a similar manner as described in [1], to a solution of 2-nitrophenol (69.7 mg, 0.5 mmol, 1 equiv) in methanol (0.50 mL) was added 3-furaldehyde (43.2 µL, 0.5 mmol, 1 equiv), propargylamine (32.0 µL, 0.5 mmol, 1 equiv), and *tert*-butyl isocyanide (114 µL, 1.0 mmol, 2 equiv). The reaction mixture was warmed at 50 °C for 30 h. Removal of volatiles gave crude material, which was purified via flash column chromatography on silica gel (20% ethyl acetate in hexanes) to afford *N*-arylamide **3e** (40.7 mg, 23%). R_f = 0.70 (50:50 EtOAc:Hex); ^{1}H NMR (400 MHz, $CDCl_3$) δ 7.68 (d, *J* = 7.7 Hz, 1H), 7.58 (s, 1H), 7.54-7.49 (m, 2H), 7.37 (s, 1H), 7.34-7.28 (m, 1H), 6.89 (s, 1H), 6.41 (s, 1H), 4.95 (s, 1H), 3.61 (dd, *J* = 17.9, 2.2 Hz, 1H), 3.53 (dd, *J* = 17.8, 2.0 Hz, 1H), 2.27 (s, 1H), 1.12 (s, 9H); ^{13}C NMR (100 MHz, $CDCl_3$) δ 168.7, 147.7, 143.9, 142.6, 141.1, 132.7, 126.7, 126.4, 124.3, 120.6, 109.2, 76.8, 75.2, 62.4, 51.2, 43.6, 28.4; DIMS $[M+H]^+$ calcd for $C_{19}H_{22}N_3O_4$ 356.1610; found 356.1601.

Synthesis of *N*-cyclohexyl-2-(furan-3-yl)-2-((2-nitrophenyl)(prop-2-yn-1-yl)amino) acetamide (3f).

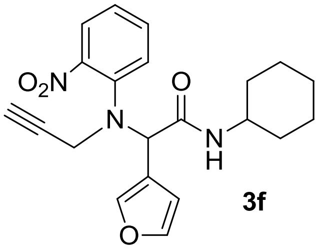

3f

In a similar manner as described in [1], to a solution of 2-nitrophenol (69.6 mg, 0.5 mmol, 1 equiv) in methanol (0.50 mL) was added 3-furaldehyde (43.3 μL, 0.5 mmol, 1 equiv), propargylamine (32.1 μL, 0.5 mmol, 1 equiv), and cyclohexyl isocyanide (62.2 μL, 0.5 mmol, 1 equiv). The reaction mixture was warmed at 50 °C for 30 h. Removal of volatiles gave crude material, which was purified via flash column chromatography on silica gel (20% ethyl acetate in hexanes) to afford *N*-arylamide **3f** (92.2 mg, 48%). R_f = 0.47 (50:50 EtOAc:Hex); ^1H NMR (400 MHz, $CDCl_3$) δ 7.65 (d, *J* = 8.0 Hz, 1H), 7.59 (s, 1H), 7.51-7.50 (m, 2H), 7.36 (s, 1H), 7.33-7.30 (m, 1H), 7.02 (d, *J* = 8.3 Hz, 1H), 6.40 (s, 1H), 5.07 (s, 1H), 3.70-3.54 (m, 3H), 2.27 (s, 1H), 1.78-1.64 (m, 3H), 1.56-1.47 (m, 2H), 1.30-1.04 (m, 4H), 0.79-0.70 (m, 1H); ^{13}C NMR (100 MHz, $CDCl_3$) δ 168.5, 147.7, 143.8, 142.6, 140.9, 132.7, 126.4(2C), 124.2, 120.5, 109.1, 76.7, 75.2, 61.4, 47.9, 43.6, 32.7, 32.4, 25.4, 24.8, 24.7; HR-FAB MS $[M+H]^+$ calcd for $C_{21}H_{24}N_3O_4$ 382.1767; found 382.1768.

Synthesis of 2-(allyl(2-nitrophenyl)amino)-*N*-cyclohexyl-2-(thiophen-2-yl)acetamide (4a).

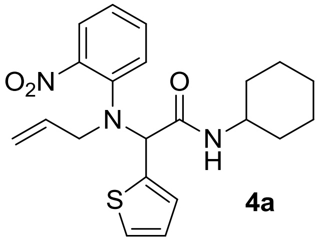

4a

In a similar manner as described in [1], to a solution of 2-nitrophenol (69.8 mg, 0.5 mmol, 1 equiv) in methanol (0.50 mL) was added 2-thiophenecarboxaldehyde (46.7 μL, 0.5 mmol, 1 equiv), allylamine (38 μL, 0.5 mmol, 1 equiv), and cyclohexyl isocyanide (62 μL, 0.5 mmol, 1 equiv). The reaction mixture was warmed at 50 °C for 30 h. Removal of volatiles gave crude material, which was purified via flash column chromatography on silica gel (10% ethyl acetate in hexanes) to afford *N*-aryl amide **4a** (49.3 mg, 25%). R_f = 0.13 (20:80 EtOAc:Hex); ^1H NMR (400 MHz, $CDCl_3$) δ 7.69 (d, *J* = 8.0 Hz, 1H), 7.45 (dd, *J* = 8.0, 7.6 Hz, 1H), 7.29-7.19 (m, 4H), 7.05 (d, *J* = 2.6 Hz, 1H), 6.91 (dd, *J* = 4.3, 4.3 Hz, 1H), 5.65-5.55 (m, 1H), 5.22 (s, 1H), 5.06 (d, *J* = 10.1 Hz, 1H), 4.91 (d, *J* = 17.1 Hz, 1H), 3.67-3.62 (m, 1H), 3.60-3.46 (m, 2H), 1.86-1.77 (m, 1H), 1.73-1.64 (m, 1H), 1.60-1.50 (m, 3H), 1.33-1.09 (m, 4H), 0.96-0.88 (m, 1H); ^{13}C NMR (100 MHz, $CDCl_3$) δ 168.4, 147.1, 141.8, 138.4, 132.7, 131.4, 128.4, 126.5, 126.4 (2C), 125.3, 124.7, 120.3, 66.8, 55.4, 48.0, 32.5, 32.4, 25.4, 24.7, 24.6; DIMS $[M+H]^+$ calcd for $C_{21}H_{26}N_3O_3S$ 400.1695; found 400.1690.

Synthesis of 2-(but-3-en-1-yl(2-nitrophenyl)amino)-*N*-cyclohexyl-2-(thiophen-2-yl) acetamide (4b).

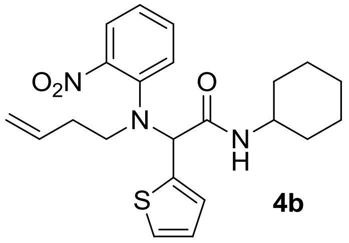

4b

In a similar manner as described in [1], to a solution of 2-nitrophenol (69.8 mg, 0.5 mmol, 1 equiv) in methanol (0.50 mL) was added 2-thiophenecarboxaldehyde (46.7 μL, 0.5 mmol, 1 equiv), 3-butenylamine (45.8 μL, 0.5 mmol, 1 equiv), and cyclohexyl isocyanide (61.3 μL, 0.5 mmol, 1 equiv). The reaction mixture was warmed at 50 °C for 48 h. Removal of volatiles gave crude material, which was purified via flash column chromatography on silica gel (20% ethyl ether in hexanes) to afford *N*-aryl amide **4b** (47.5 mg, 23%). R_f = 0.10 (40:60 Et_2O:Hex); ^1H NMR (400 MHz, $CDCl_3$) δ 7.70 (dd, *J* = 8.1, 1.6 Hz, 1H), 7.46 (dd, *J* = 8.7, 7.0 Hz, 1H), 7.30-7.18 (m, 4H), 6.96 (d, *J* = 3.5 Hz, 1H), 6.91 (dd, *J* = 5.1, 3.5 Hz, 1H), 5.60-5.49 (m, 1H), 5.20 (s, 1H), 4.92 (dd, *J* = 10.2, 1.6 Hz, 1H), 4.86 (dd, *J* = 17.1, 1.6 Hz, 1H), 3.74-3.64 (m, 1H), 3.08-2.89 (m, 2H), 2.10-1.99 (m, 1H), 1.98-1.90 (m, 1H), 1.89-1.82 (m, 1H), 1.74-1.66 (m, 1H), 1.66-1.56 (m, 2H), 1.35-1.13 (m, 5H), 1.06-0.96 (m, 1H); ^{13}C NMR (100 MHz, $CDCl_3$) δ 168.5, 147.8, 141.8, 137.6, 134.9, 132.8, 128.7, 126.9, 126.6, 126.4, 125.6, 124.9, 117.2, 67.5, 51.3, 48.2, 32.8, 32.6, 31.0, 25.6, 24.8, 24.7; DIMS $[M+H]^+$ calcd for $C_{22}H_{28}N_3O_3S$ 414.1851; found 414.1845.

Stacked ^{1}H NMRs for reaction monitoring (related to Scheme 2)

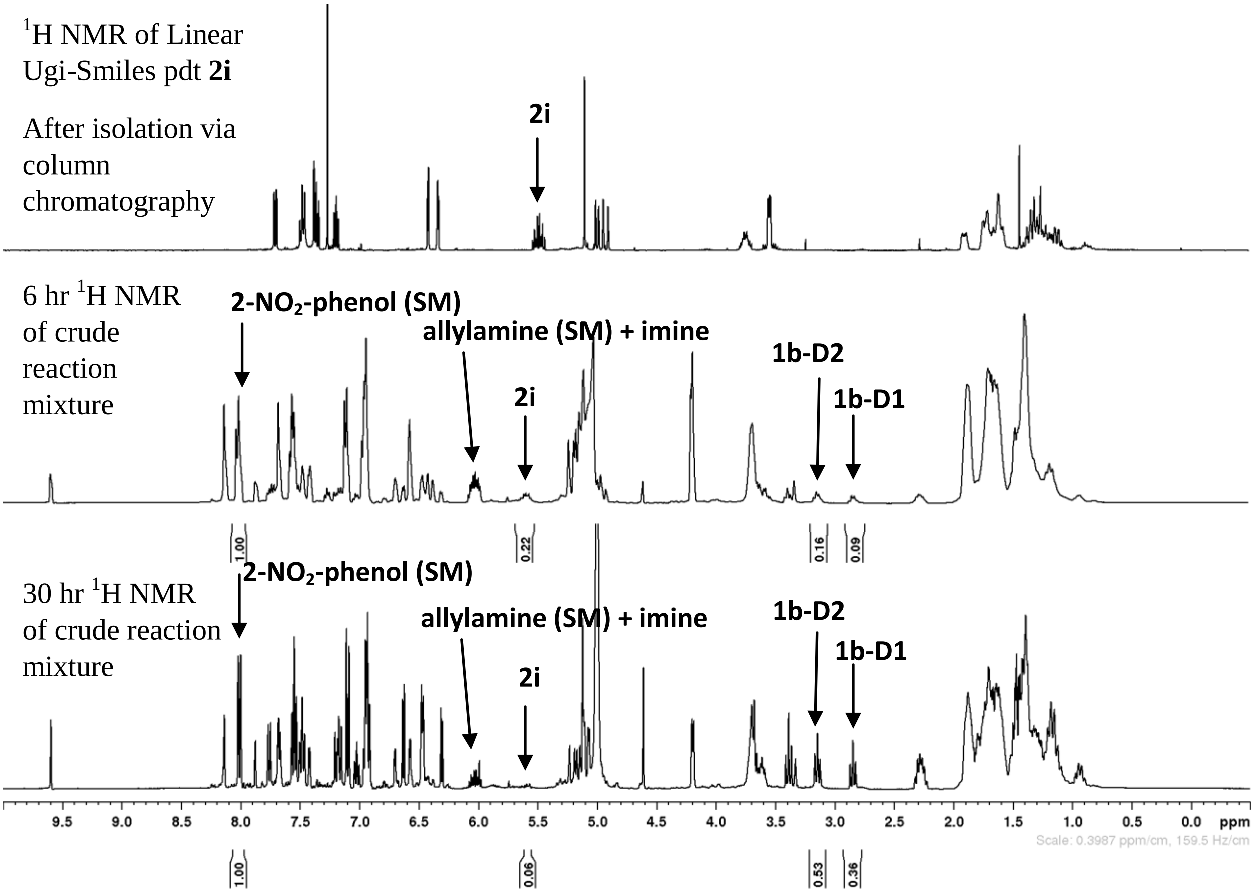

All ^{1}H spectra were acquired in $CDCl_3$. Formation of linear Ugi-Smiles adduct **2i** was tracked by the appearance of the representative diagnostic allylic peak (marked **2i** above), as that peak did not overall with other starting material or US-IMDA product peaks. Formation of diastereomers **1b-D1** and **1b-D2** were tracked by the diagnostic dd between 2.5-3.2 ppm, as these did not overlap with starting material or linear **2i** product peaks. Conversion was estimated by integrations.

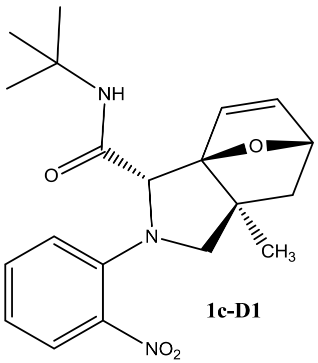

1c-D1

^{1}H NMR, $CDCl_3$

400 MHz

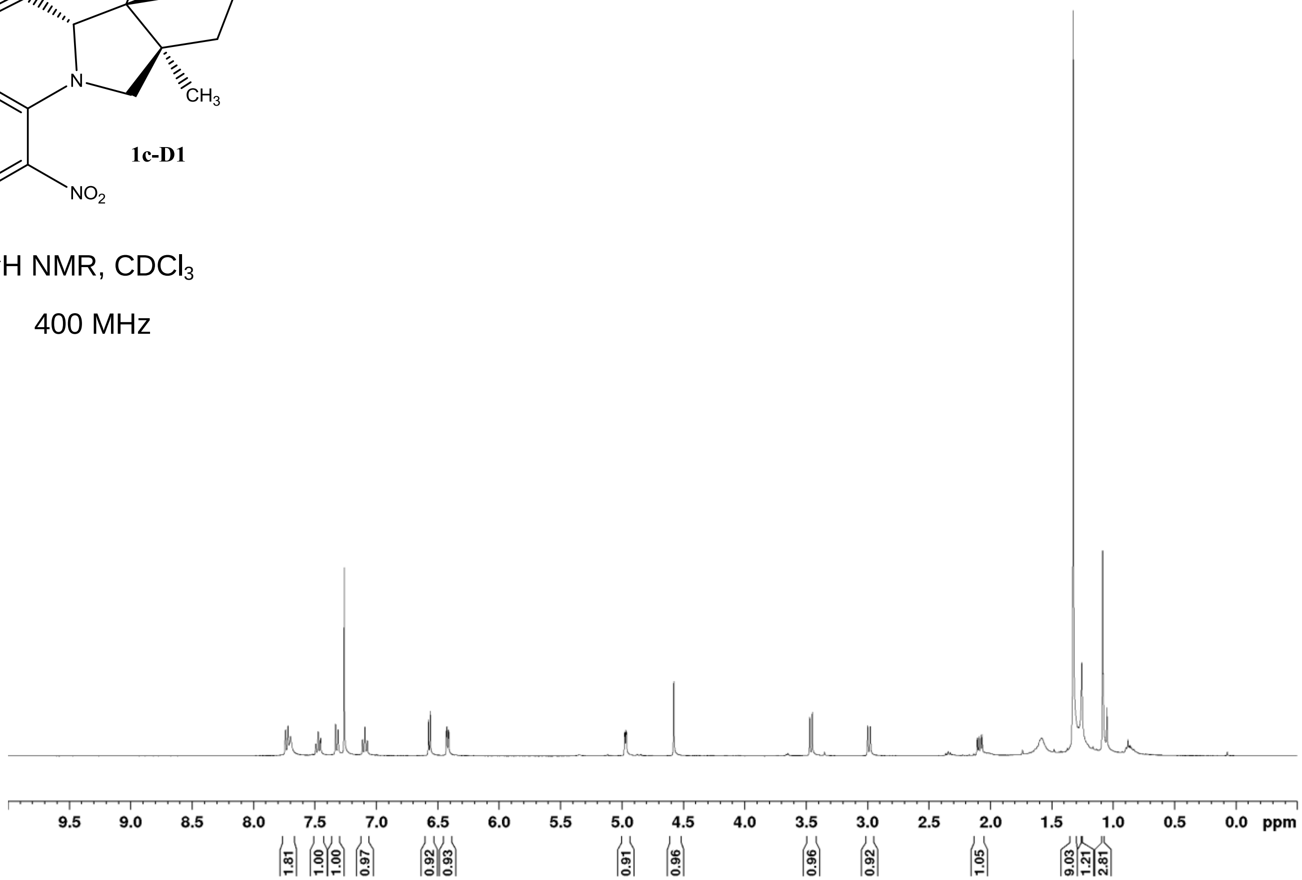


```
Current Data Parameters
NAME     methyl allylamine D
EXPNO              1
PROCNO             1

F2 - Acquisition Parameters
Date_          20160718
Time               2.38
INSTRUM           spect
PROBHD   5 mm PABBO BB/
PULPROG            zg30
TD                65536
SOLVENT           CDCl3
NS                   32
DS                    2
SWH            8012.820 Hz
FIDRES         0.122266 Hz
AQ            4.0894465 sec
RG               188.11
DW               62.400 usec
DE                 6.50 usec
TE                298.2 K
D1           1.00000000 sec
TD0                   1

======== CHANNEL f1 ========
SFO1        400.1524711 MHz
NUC1                 1H
P1                15.00 usec
PLW1         9.80000019 W

F2 - Processing parameters
SI                65536
SF          400.1500099 MHz
WDW                  EM
SSB      0
LB                 0.30 Hz
GB       0
PC                 1.00
```

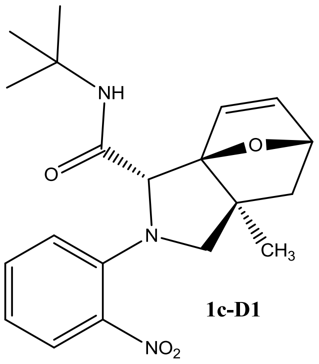

1c-D1

^{13}C NMR, $CDCl_3$

100 MHz

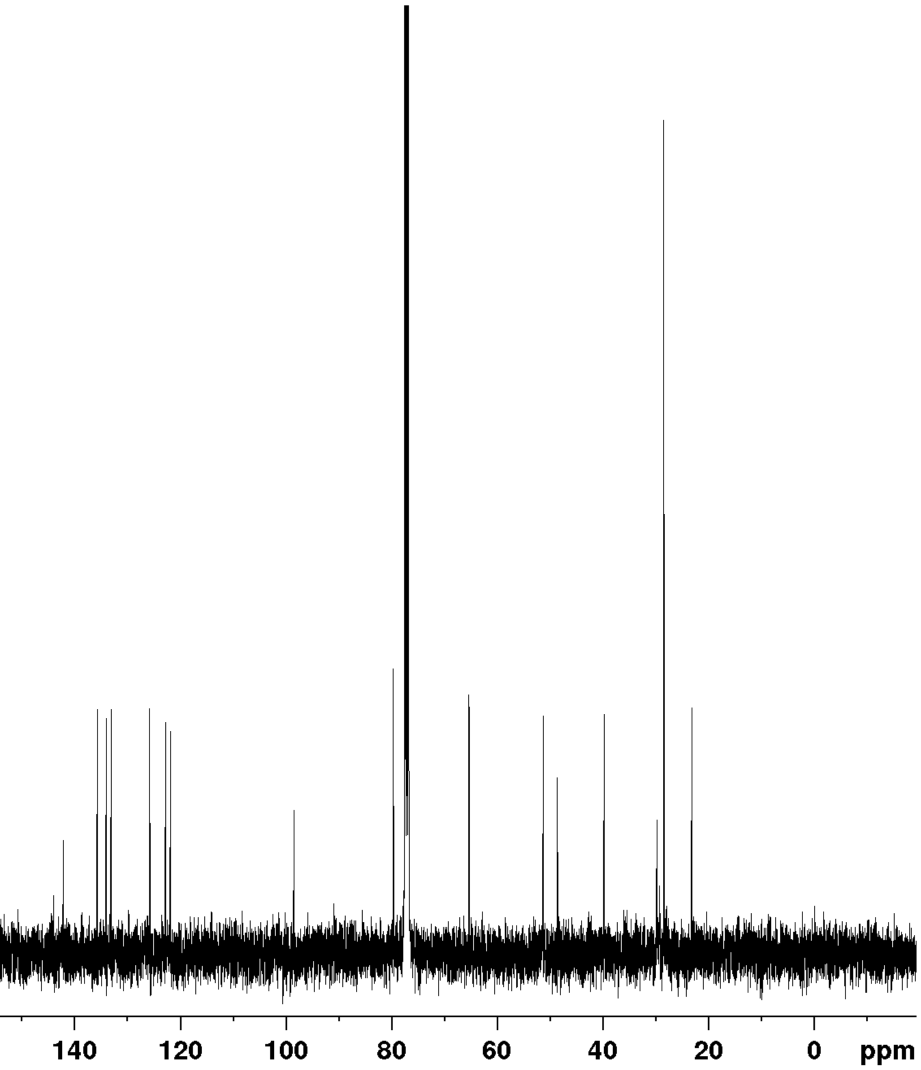


```
Current Data Parameters
NAME       KM-35-D1-C-again
EXPNO                    20
PROCNO                    1

F2 - Acquisition Parameters
Date_              20150526
Time                  21.41
INSTRUM               spect
PROBHD     5 mm PABBO BB/
PULPROG             zgpg30
TD                   65536
SOLVENT              CDCl3
NS                    2048
DS                       4
SWH              24038.461 Hz
FIDRES            0.366798 Hz
AQ               1.3631488 sec
RG                  210.96
DW                  20.800 usec
DE                   10.00 usec
TE                   293.3 K
D1              2.00000000 sec
D11             0.03000000 sec
TD0                      1

======== CHANNEL f1 ========
SFO1          100.6278588 MHz
NUC1                   13C
P1                   10.00 usec
PLW1           48.20000076 W

======== CHANNEL f2 ========
SFO2          400.1516006 MHz
NUC2                    1H
CPDPRG[2           waltz16
PCPD2                90.00 usec
PLW2            9.80000019 W
PLW12           0.27221999 W
PLW13           0.22050001 W

F2 - Processing parameters
SI                   32768
SF            100.6177873 MHz
WDW                     EM
SSB      0
LB                    1.00 Hz
GB       0
PC                    1.40
```

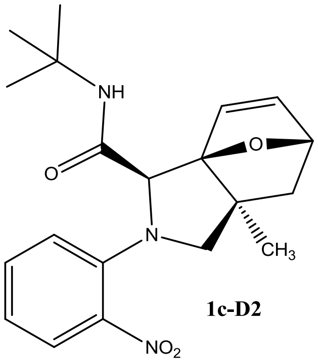

1c-D2

^{1}H NMR, $CDCl_3$

400 MHz

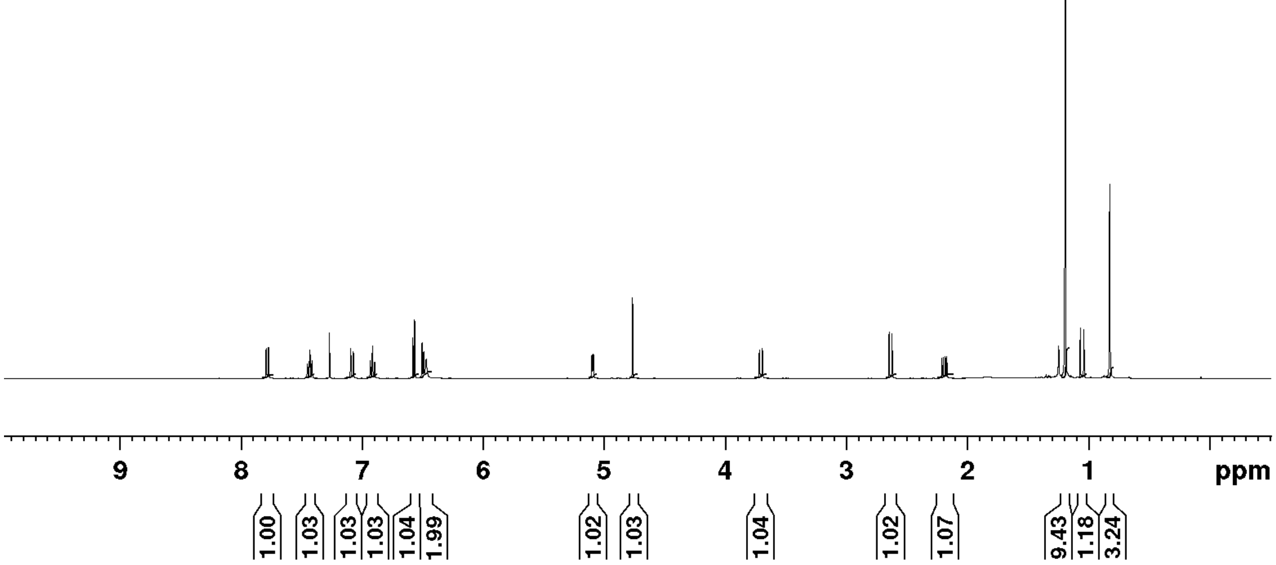


```
Current Data Parameters
NAME              KM-35-D2
EXPNO                    1
PROCNO                   1

F2 - Acquisition Parameters
Date_             20150427
Time                 11.10
INSTRUM              spect
PROBHD    5 mm PABBO BB/
PULPROG               zg30
TD                   65536
SOLVENT              CDCl3
NS                      16
DS                       2
SWH               8012.820 Hz
FIDRES            0.122266 Hz
AQ               4.0894465 sec
RG                   95.15
DW                  62.400 usec
DE                    6.50 usec
TE                   292.4 K
D1              1.00000000 sec
TD0                      1

======== CHANNEL f1 ========
SFO1           400.1524711 MHz
NUC1                    1H
P1                   15.00 usec
PLW1            9.80000019 W

F2 - Processing parameters
SI                   65536
SF             400.1500076 MHz
WDW                     EM
SSB      0
LB                    0.30 Hz
GB       0
PC                    1.00
```

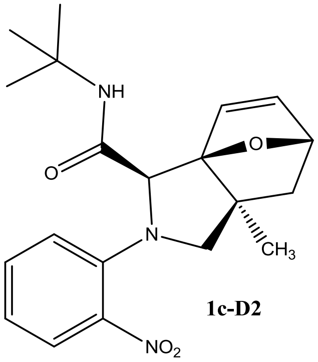

1c-D2

^{13}C NMR, $CDCl_3$

100 MHz

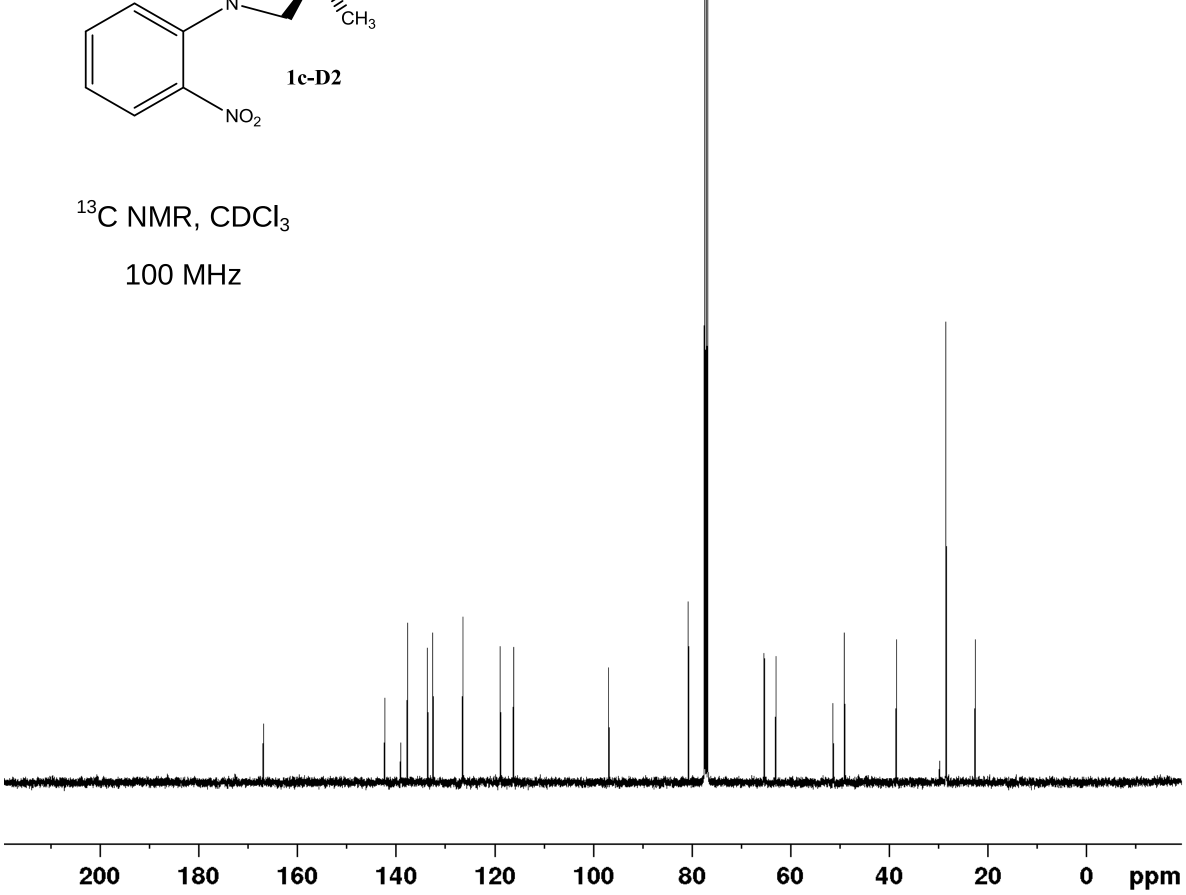

BRUKER

```
Current Data Parameters
NAME          KM-35-D2-C
EXPNO                  1
PROCNO                 1

F2 - Acquisition Parameters
Date_           20150522
Time               12.52
INSTRUM            spect
PROBHD   5 mm PABBO BB/
PULPROG          zgpg30
TD                 65536
SOLVENT            CDCl3
NS                   512
DS                     4
SWH            24038.461 Hz
FIDRES          0.366798 Hz
AQ             1.3631488 sec
RG                210.96
DW                20.800 usec
DE                 10.00 usec
TE                 293.8 K
D1            2.00000000 sec
D11           0.03000000 sec
TD0                    1

======== CHANNEL f1 ========
SFO1         100.6278588 MHz
NUC1                 13C
P1                 10.00 usec
PLW1         48.20000076 W

======== CHANNEL f2 ========
SFO2         400.1516006 MHz
NUC2                  1H
CPDPRG[2         waltz16
PCPD2              90.00 usec
PLW2          9.80000019 W
PLW12         0.27221999 W
PLW13         0.22050001 W

F2 - Processing parameters
SI                 32768
SF           100.6177874 MHz
WDW                   EM
SSB      0
LB                  1.00 Hz
GB       0
PC                  1.40
```

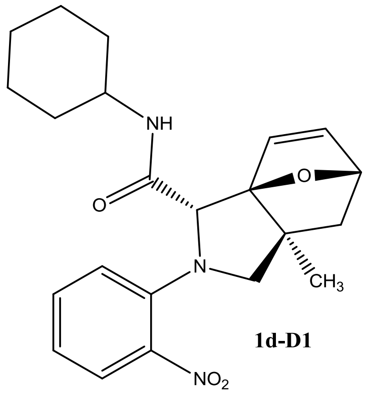

1d-D1

^{1}H NMR, $CDCl_3$

400 MHz

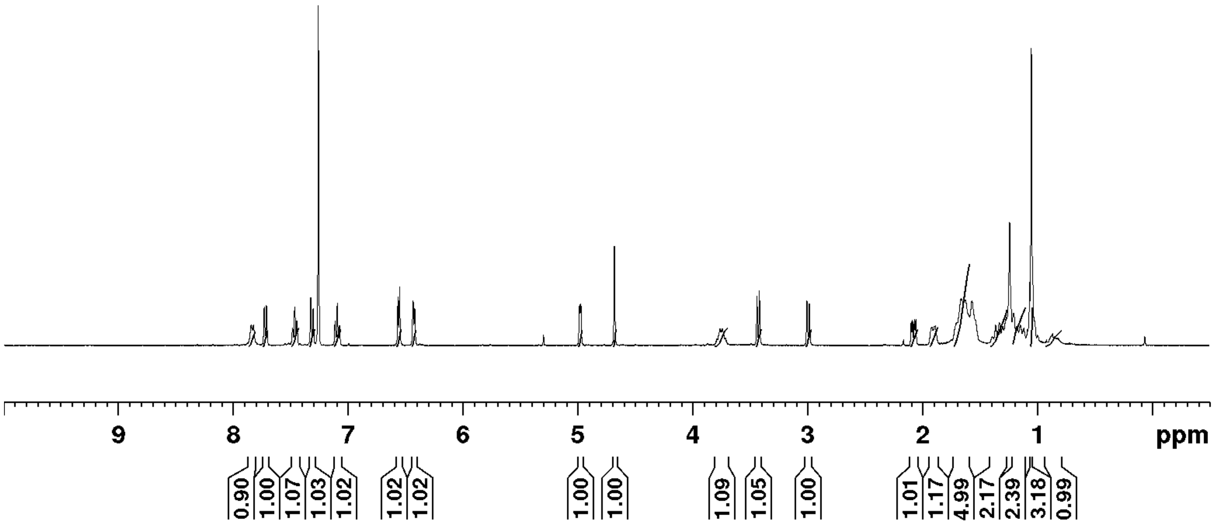

Current Data Parameters
NAME KM-38-D1R-2-F23-25
EXPNO 10
PROCNO 1

F2 - Acquisition Parameters
Date_ 20150515
Time 11.14
INSTRUM spect
PROBHD 5 mm PABBO BB/
PULPROG zg30
TD 65536
SOLVENT CDCl3
NS 16
DS 2
SWH 8012.820 Hz
FIDRES 0.122266 Hz
AQ 4.0894465 sec
RG 166.66
DW 62.400 usec
DE 6.50 usec
TE 292.3 K
D1 1.00000000 sec
TD0 1

======== CHANNEL f1 ========
SFO1 400.1524711 MHz
NUC1 1H
P1 15.00 usec
PLW1 9.80000019 W

F2 - Processing parameters
SI 65536
SF 400.1500120 MHz
WDW EM
SSB 0
LB 0.30 Hz
GB 0
PC 1.00

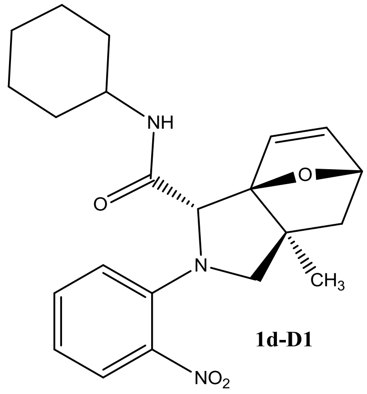

1d-D1

^{13}C NMR, $CDCl_3$

100 MHz

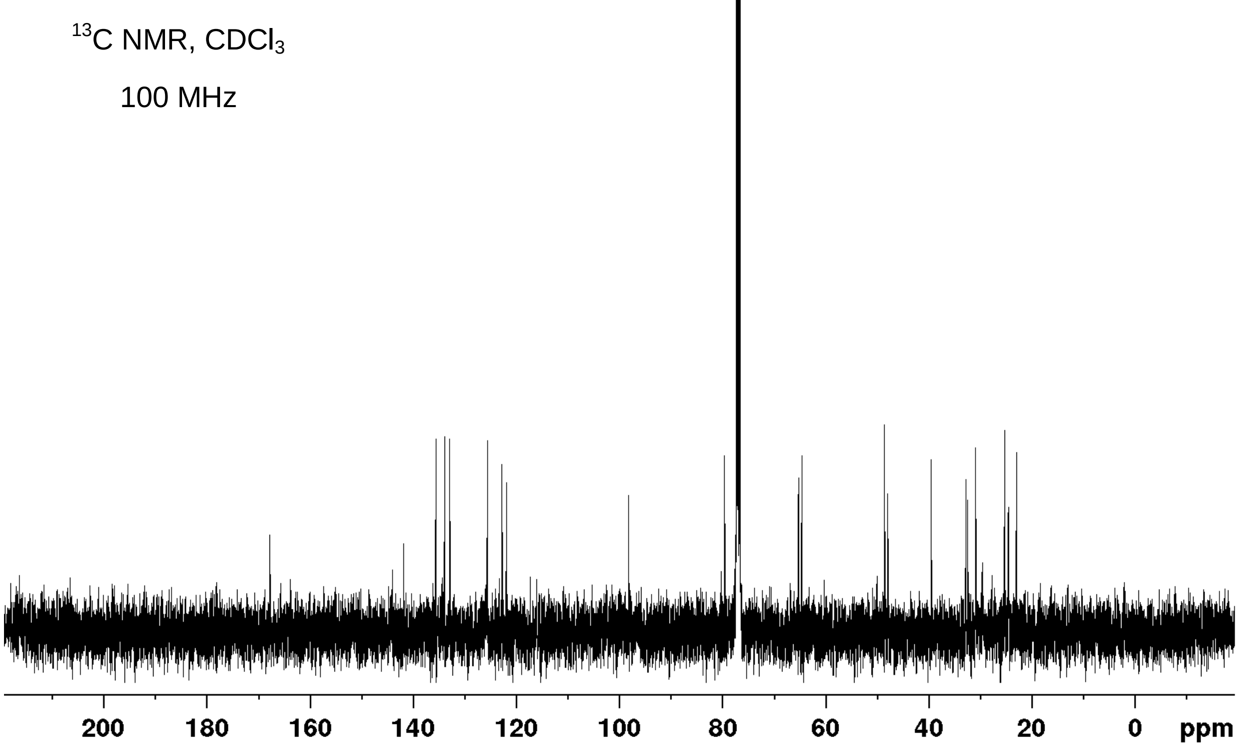


```
Current Data Parameters
NAME       KM-38-D1R-2-F23-25-C2
EXPNO                 10
PROCNO                 1

F2 - Acquisition Parameters
Date_           20150519
Time               13.19
INSTRUM            spect
PROBHD   5 mm PABBO BB/
PULPROG          zgpg30
TD                 65536
SOLVENT            CDCl3
NS                  1024
DS                     4
SWH            24038.461 Hz
FIDRES          0.366798 Hz
AQ             1.3631488 sec
RG                210.96
DW                20.800 usec
DE                 10.00 usec
TE                 293.8 K
D1            2.00000000 sec
D11           0.03000000 sec
TD0                    1

======== CHANNEL f1 ========
SFO1         100.6278588 MHz
NUC1                 13C
P1                 10.00 usec
PLW1         48.20000076 W

======== CHANNEL f2 ========
SFO2         400.1516006 MHz
NUC2                  1H
CPDPRG[2         waltz16
PCPD2              90.00 usec
PLW2          9.80000019 W
PLW12         0.27221999 W
PLW13         0.22050001 W

F2 - Processing parameters
SI                 32768
SF           100.6178032 MHz
WDW                   EM
SSB      0
LB                  1.00 Hz
GB       0
PC                  1.40
```

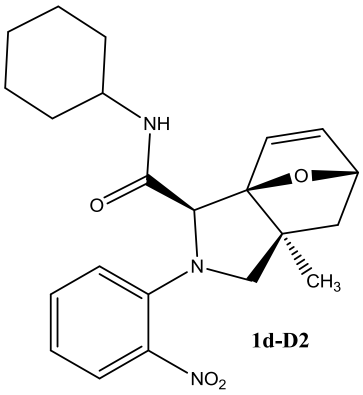

1d-D2

^{1}H NMR, $CDCl_3$

400 MHz

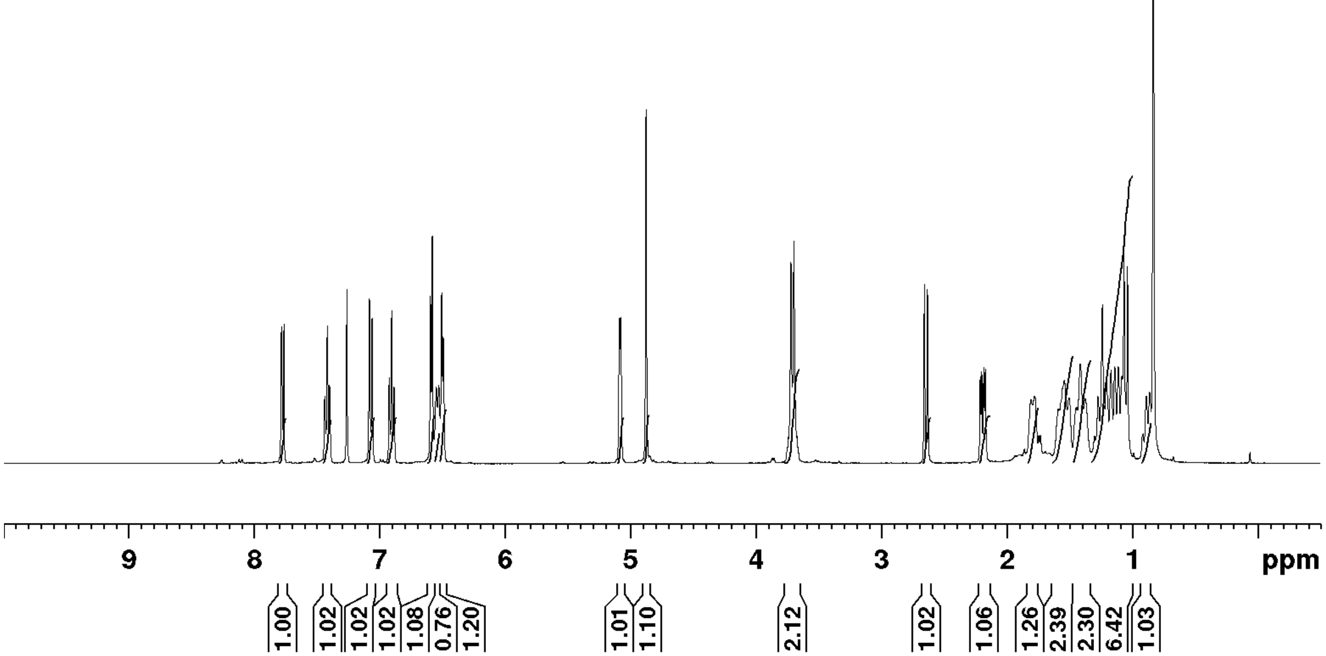

BRUKER

```
Current Data Parameters
NAME      KM-38-F63-80-D2
EXPNO                  10
PROCNO                  1

F2 - Acquisition Parameters
Date_            20150512
Time                14.29
INSTRUM             spect
PROBHD    5 mm PABBO BB/
PULPROG              zg30
TD                  65536
SOLVENT             CDCl3
NS                     16
DS                      2
SWH              8012.820 Hz
FIDRES           0.122266 Hz
AQ              4.0894465 sec
RG                 120.42
DW                 62.400 usec
DE                   6.50 usec
TE                  292.4 K
D1             1.00000000 sec
TD0                     1

======== CHANNEL f1 ========
SFO1          400.1524711 MHz
NUC1                   1H
P1                  15.00 usec
PLW1           9.80000019 W

F2 - Processing parameters
SI                  65536
SF            400.1500104 MHz
WDW                    EM
SSB       0
LB                   0.30 Hz
GB        0
PC                   1.00
```

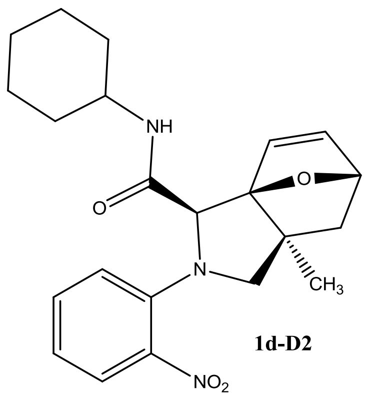

1d-D2

^{13}C NMR, $CDCl_3$

100 MHz

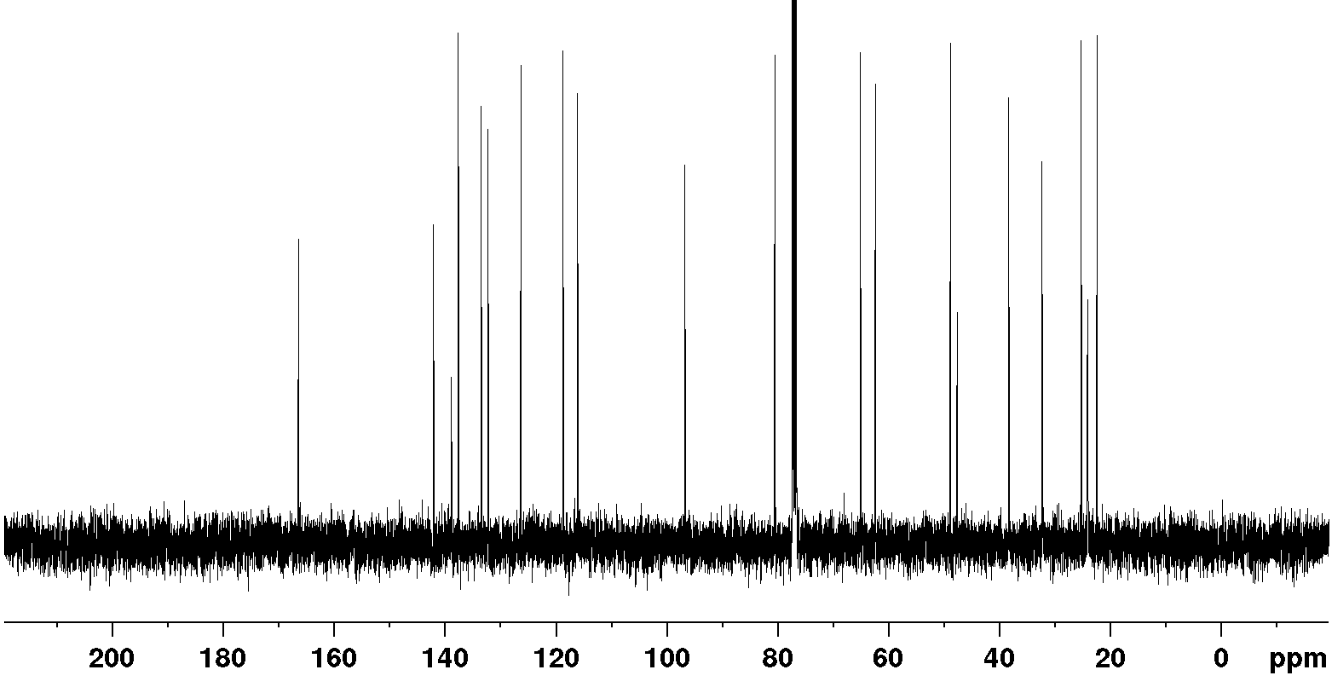


```
Current Data Parameters
NAME      KM-38-F63-80-D2
EXPNO                  20
PROCNO                  1

F2 - Acquisition Parameters
Date_            20150512
Time                14.38
INSTRUM             spect
PROBHD    5 mm PABBO BB/
PULPROG           zgpg30
TD                 65536
SOLVENT            CDCl3
NS                   128
DS                     4
SWH            24038.461 Hz
FIDRES          0.366798 Hz
AQ             1.3631488 sec
RG                210.96
DW                20.800 usec
DE                 10.00 usec
TE                 293.4 K
D1            2.00000000 sec
D11           0.03000000 sec
TD0                    1

======== CHANNEL f1 ========
SFO1         100.6278588 MHz
NUC1                 13C
P1                 10.00 usec
PLW1         48.20000076 W

======== CHANNEL f2 ========
SFO2         400.1516006 MHz
NUC2                  1H
CPDPRG[2         waltz16
PCPD2              90.00 usec
PLW2          9.80000019 W
PLW12         0.27221999 W
PLW13         0.22050001 W

F2 - Processing parameters
SI                 32768
SF           100.6177903 MHz
WDW                   EM
SSB      0
LB                  1.00 Hz
GB       0
PC                  1.40
```

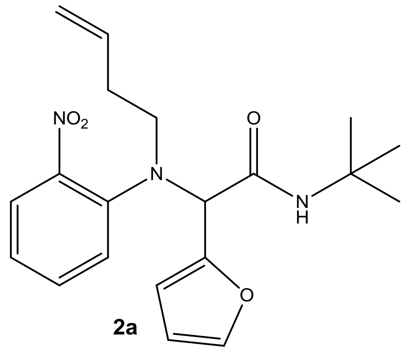

2a

^1H NMR, $CDCl_3$

400 MHz

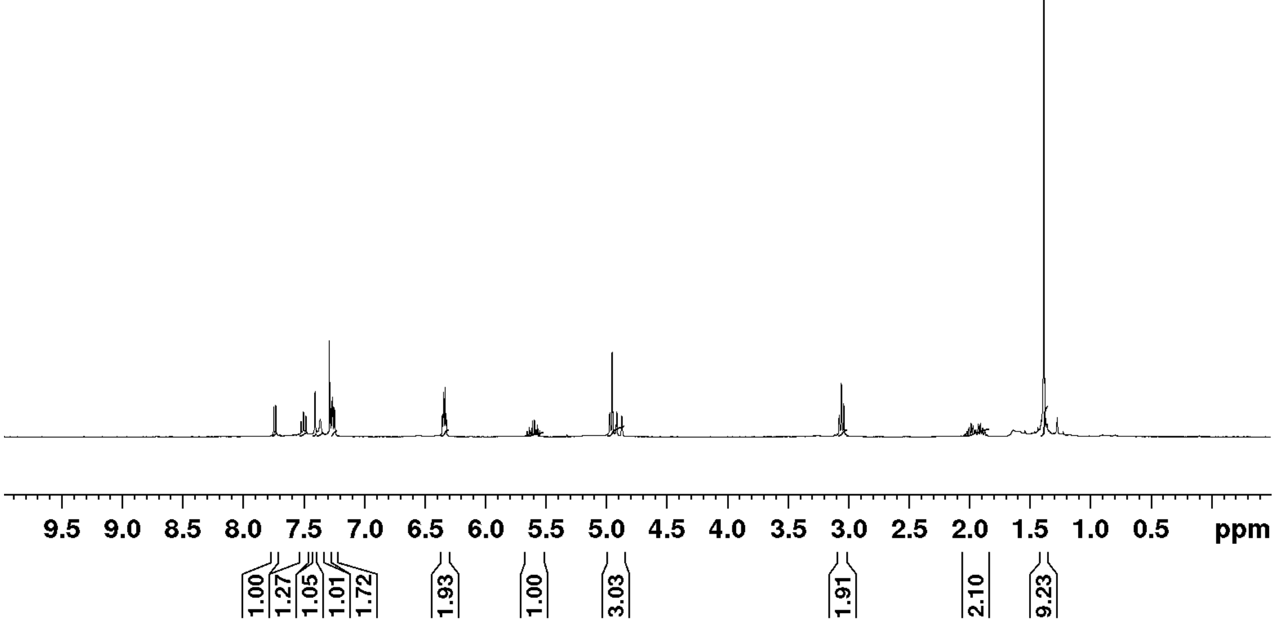


```
Current Data Parameters
NAME       KM-34-F31-41-test
EXPNO                   10
PROCNO                   1

F2 - Acquisition Parameters
Date_             20150204
Time                 15.13
INSTRUM              spect
PROBHD     5 mm PABBO BB/
PULPROG               zg30
TD                   65536
SOLVENT              CDCl3
NS                      16
DS                       2
SWH              8012.820 Hz
FIDRES           0.122266 Hz
AQ              4.0894465 sec
RG                   134.4
DW                  62.400 usec
DE                    6.50 usec
TE                   293.6 K
D1             1.00000000 sec
TD0                      1

======== CHANNEL f1 ========
SFO1          400.1524711 MHz
NUC1                    1H
P1                   15.00 usec
PLW1           9.80000019 W

F2 - Processing parameters
SI                   65536
SF            400.1500000 MHz
WDW                     EM
SSB      0
LB                    0.30 Hz
GB       0
PC                    1.00
```

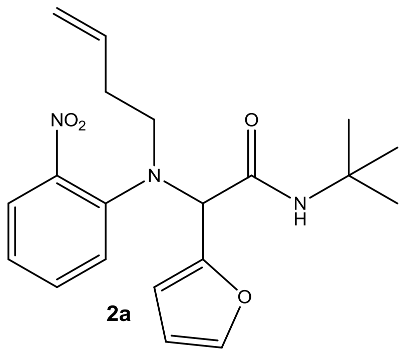

2a

^{13}C NMR, $CDCl_3$

100 MHz

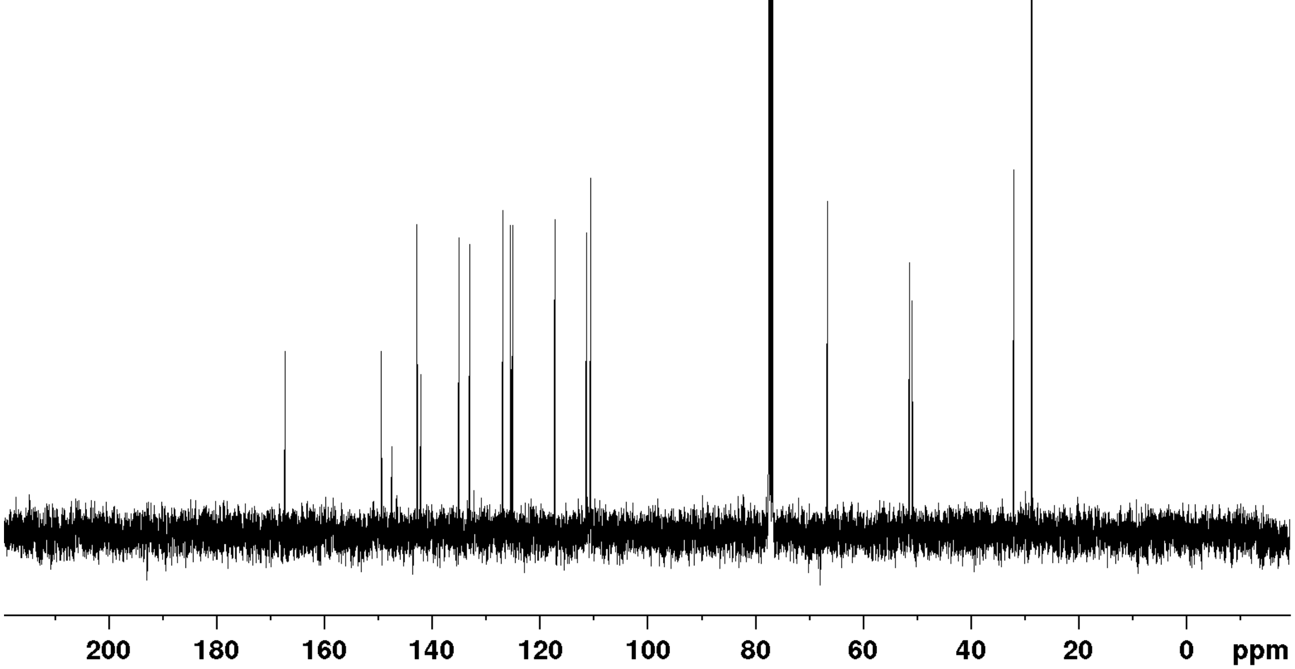


```
Current Data Parameters
NAME       KM-34-F31-41-C
EXPNO                  10
PROCNO                  1

F2 - Acquisition Parameters
Date_            20150202
Time                10.39
INSTRUM             spect
PROBHD     5 mm PABBO BB/
PULPROG           zgpg30
TD                  65536
SOLVENT             CDCl3
NS                    128
DS                      4
SWH             24038.461 Hz
FIDRES           0.366798 Hz
AQ              1.3631488 sec
RG                 210.96
DW                 20.800 usec
DE                  10.00 usec
TE                  294.7 K
D1             2.00000000 sec
D11            0.03000000 sec
TD0                     1

======== CHANNEL f1 ========
SFO1          100.6278588 MHz
NUC1                  13C
P1                  10.00 usec
PLW1          48.20000076 W

======== CHANNEL f2 ========
SFO2          400.1516006 MHz
NUC2                   1H
CPDPRG[2          waltz16
PCPD2               90.00 usec
PLW2           9.80000019 W
PLW12          0.27221999 W
PLW13          0.22050001 W

F2 - Processing parameters
SI                  32768
SF            100.6177859 MHz
WDW                    EM
SSB       0
LB                   1.00 Hz
GB        0
PC                   1.40
```

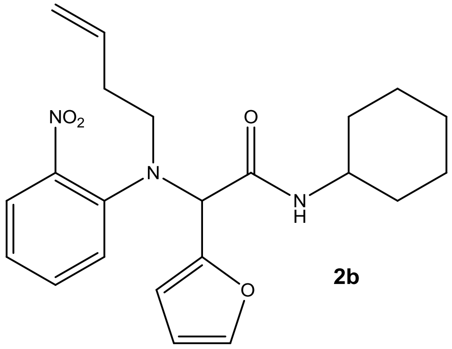

2b

^{1}H NMR, $CDCl_3$

400 MHz

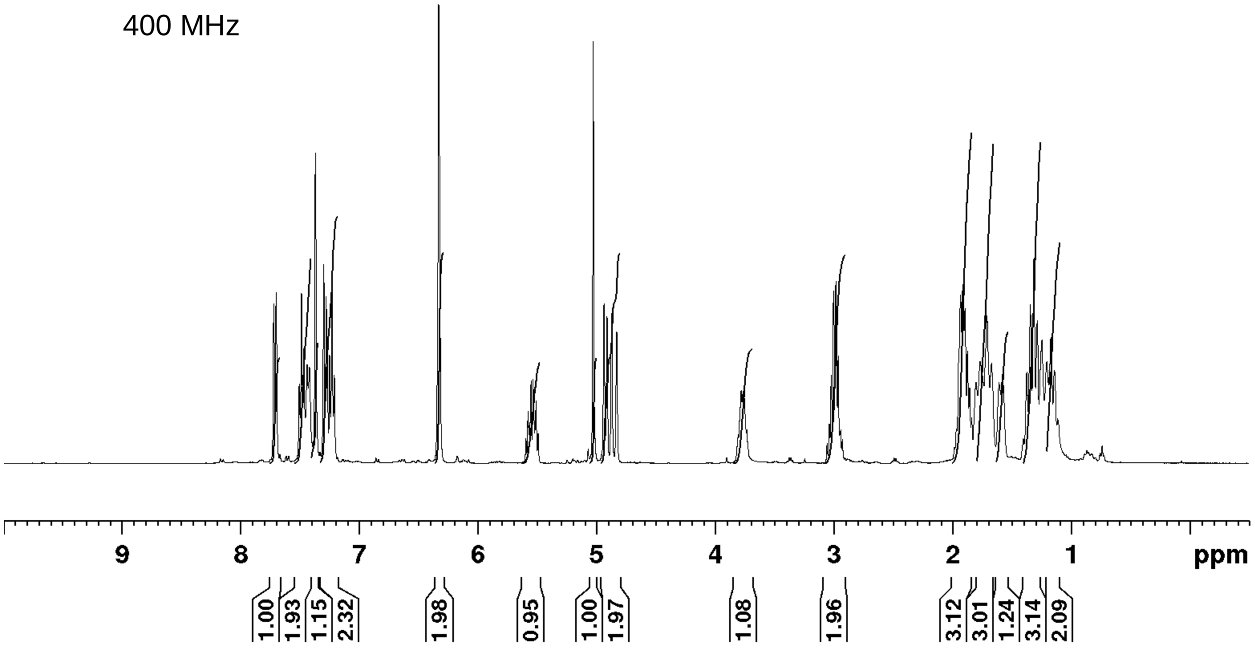


```
Current Data Parameters
NAME            MM-2-70
EXPNO                 1
PROCNO                1

F2 - Acquisition Parameters
Date_          20160307
Time              20.28
INSTRUM           spect
PROBHD   5 mm PABBO BB/
PULPROG            zg30
TD                65536
SOLVENT           CDCl3
NS                   16
DS                    2
SWH            8012.820 Hz
FIDRES         0.122266 Hz
AQ            4.0894465 sec
RG                75.54
DW               62.400 usec
DE                 6.50 usec
TE                296.1 K
D1           1.00000000 sec
TD0                   1

======== CHANNEL f1 ========
SFO1        400.1524711 MHz
NUC1                 1H
P1                15.00 usec
PLW1         9.80000019 W

F2 - Processing parameters
SI                65536
SF          400.1500081 MHz
WDW                  EM
SSB      0
LB                 0.30 Hz
GB       0
PC                 1.00
```

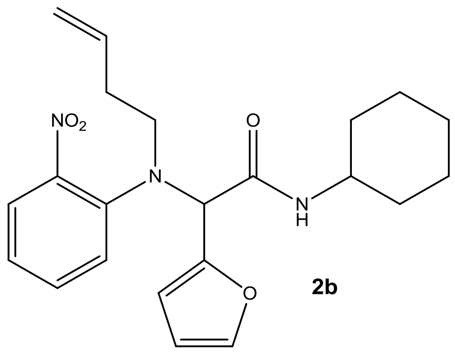

2b

^{13}C NMR, $CDCl_3$

100 MHz

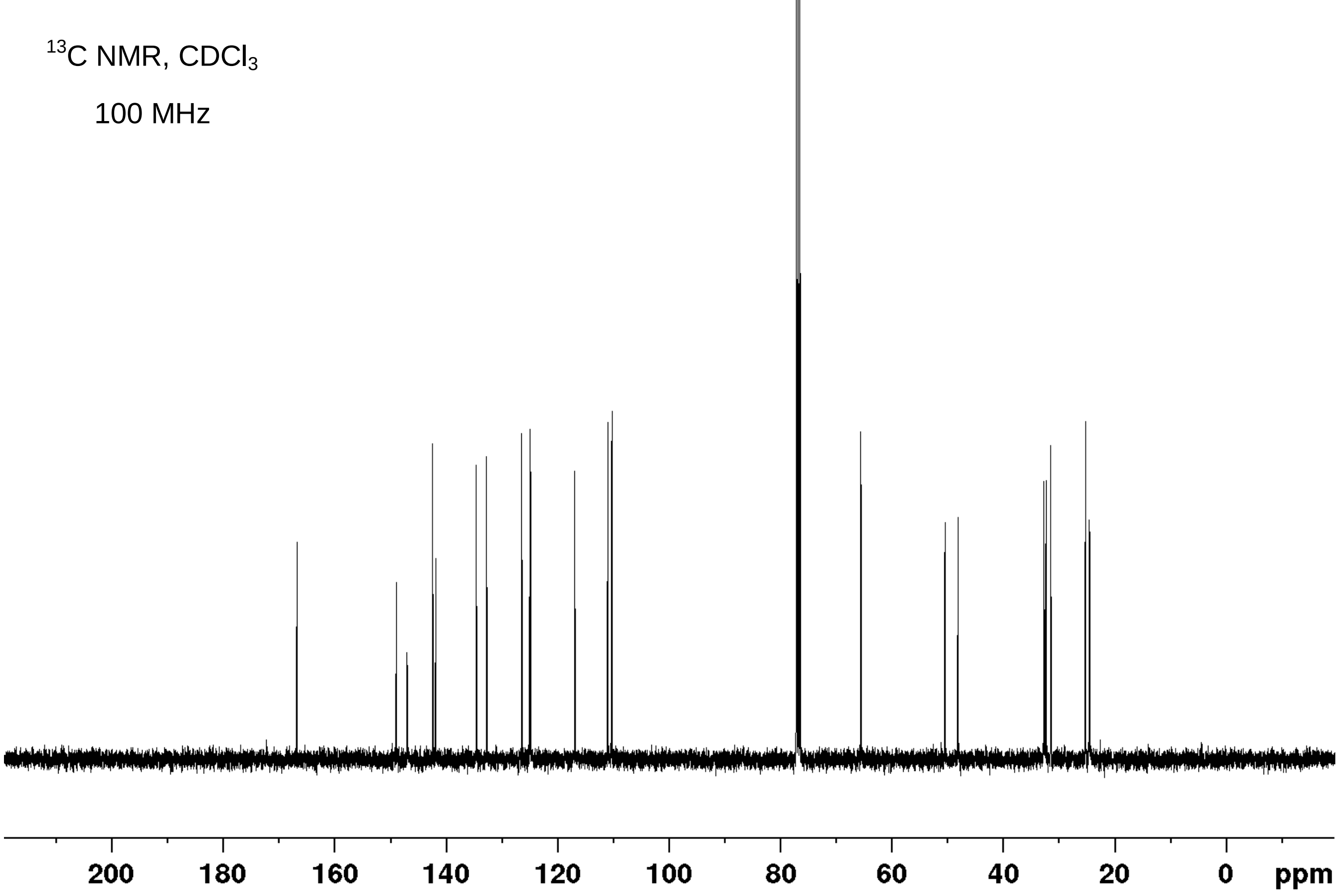

BRUKER

```
Current Data Parameters
NAME                MM-2-70
EXPNO                     2
PROCNO                    1

F2 - Acquisition Parameters
Date_              20160307
Time                  20.36
INSTRUM               spect
PROBHD    5 mm PABBO BB/
PULPROG             zgpg30
TD                   65536
SOLVENT              CDCl3
NS                     128
DS                       4
SWH              24038.461 Hz
FIDRES            0.366798 Hz
AQ               1.3631488 sec
RG                  210.96
DW                  20.800 usec
DE                   10.00 usec
TE                   297.1 K
D1              2.00000000 sec
D11             0.03000000 sec
TD0                      1

======== CHANNEL f1 ========
SFO1          100.6278588 MHz
NUC1                  13C
P1                   10.00 usec
PLW1          48.20000076 W

======== CHANNEL f2 ========
SFO2          400.1516006 MHz
NUC2                   1H
CPDPRG[2           waltz16
PCPD2                90.00 usec
PLW2           9.80000019 W
PLW12          0.27221999 W
PLW13          0.22050001 W

F2 - Processing parameters
SI                   32768
SF             100.6178041 MHz
WDW                     EM
SSB      0
LB                    1.00 Hz
GB       0
PC                    1.40
```

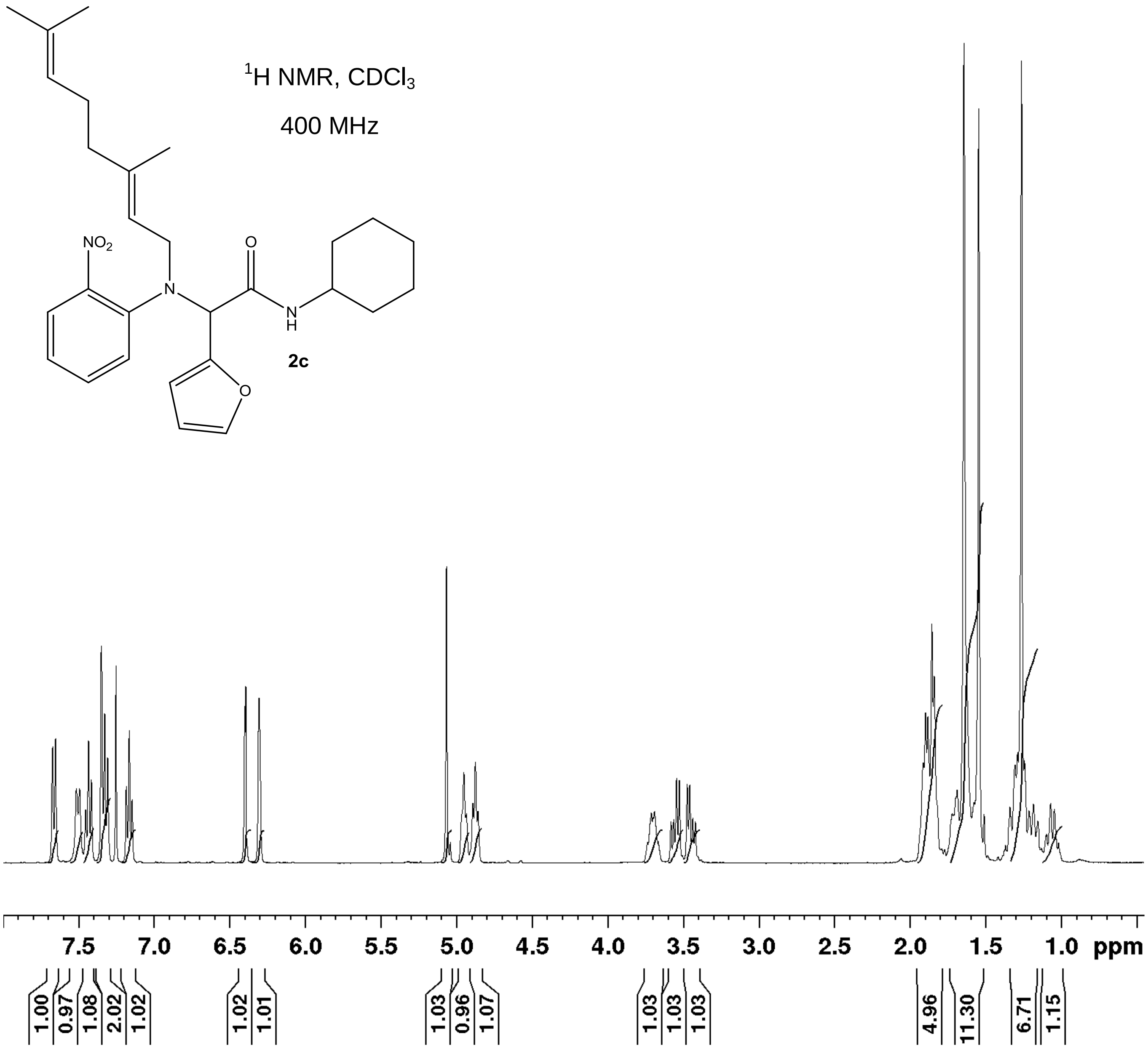

2c

^{1}H NMR, $CDCl_3$

400 MHz

BRUKER

```
Current Data Parameters
NAME                MM-2-63
EXPNO                     1
PROCNO                    1

F2 - Acquisition Parameters
Date_              20160307
Time                  20.14
INSTRUM               spect
PROBHD     5 mm PABBO BB/
PULPROG                zg30
TD                    65536
SOLVENT               CDCl3
NS                       16
DS                        2
SWH               8012.820 Hz
FIDRES            0.122266 Hz
AQ               4.0894465 sec
RG                   120.42
DW                   62.400 usec
DE                     6.50 usec
TE                    295.9 K
D1              1.00000000 sec
TD0                       1

======== CHANNEL f1 ========
SFO1           400.1524711 MHz
NUC1                     1H
P1                    15.00 usec
PLW1             9.80000019 W

F2 - Processing parameters
SI                    65536
SF             400.1500122 MHz
WDW                      EM
SSB      0
LB                     0.30 Hz
GB       0
PC                     1.00
```

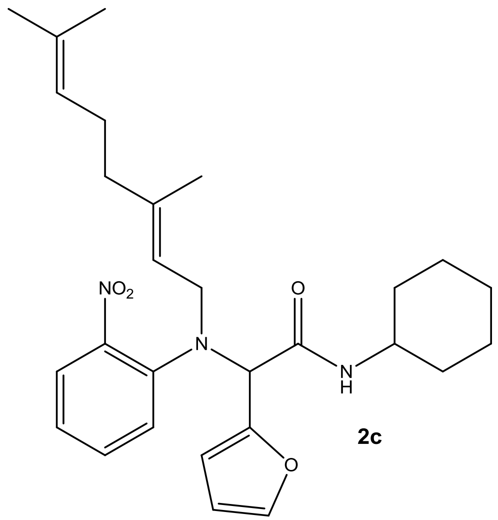

2c

^{13}C NMR, $CDCl_3$

100 MHz

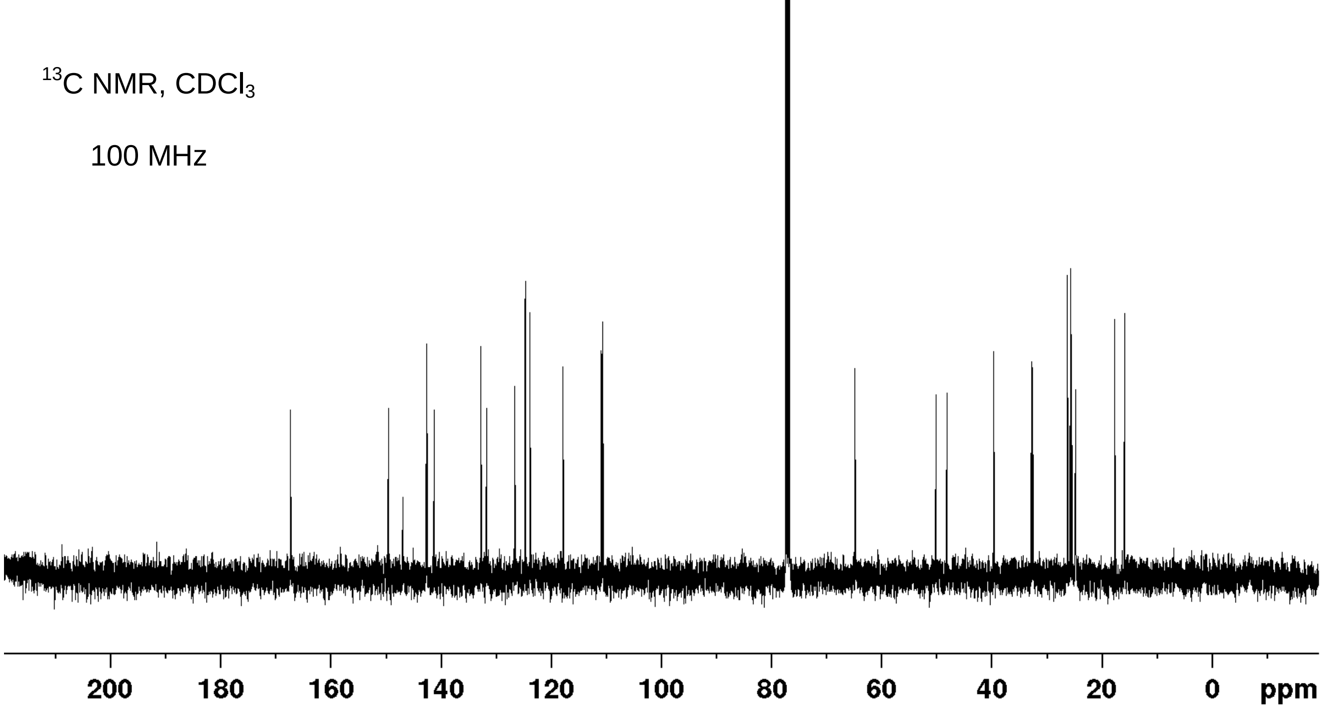

Current Data Parameters
NAME MM-2-63
EXPNO 2
PROCNO 1

F2 - Acquisition Parameters
Date_ 20160307
Time 20.23
INSTRUM spect
PROBHD 5 mm PABBO BB/
PULPROG zgpg30
TD 65536
SOLVENT CDCl3
NS 128
DS 4
SWH 24038.461 Hz
FIDRES 0.366798 Hz
AQ 1.3631488 sec
RG 210.96
DW 20.800 usec
DE 10.00 usec
TE 297.0 K
D1 2.00000000 sec
D11 0.03000000 sec
TD0 1

======== CHANNEL f1 ========
SFO1 100.6278588 MHz
NUC1 13C
P1 10.00 usec
PLW1 48.20000076 W

======== CHANNEL f2 ========
SFO2 400.1516006 MHz
NUC2 1H
CPDPRG[2 waltz16
PCPD2 90.00 usec
PLW2 9.80000019 W
PLW12 0.27221999 W
PLW13 0.22050001 W

F2 - Processing parameters
SI 32768
SF 100.6177919 MHz
WDW EM
SSB 0
LB 1.00 Hz
GB 0
PC 1.40

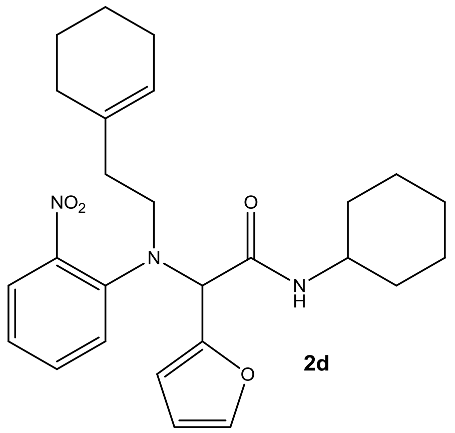

2d

^{1}H NMR, $CDCl_3$

400 MHz

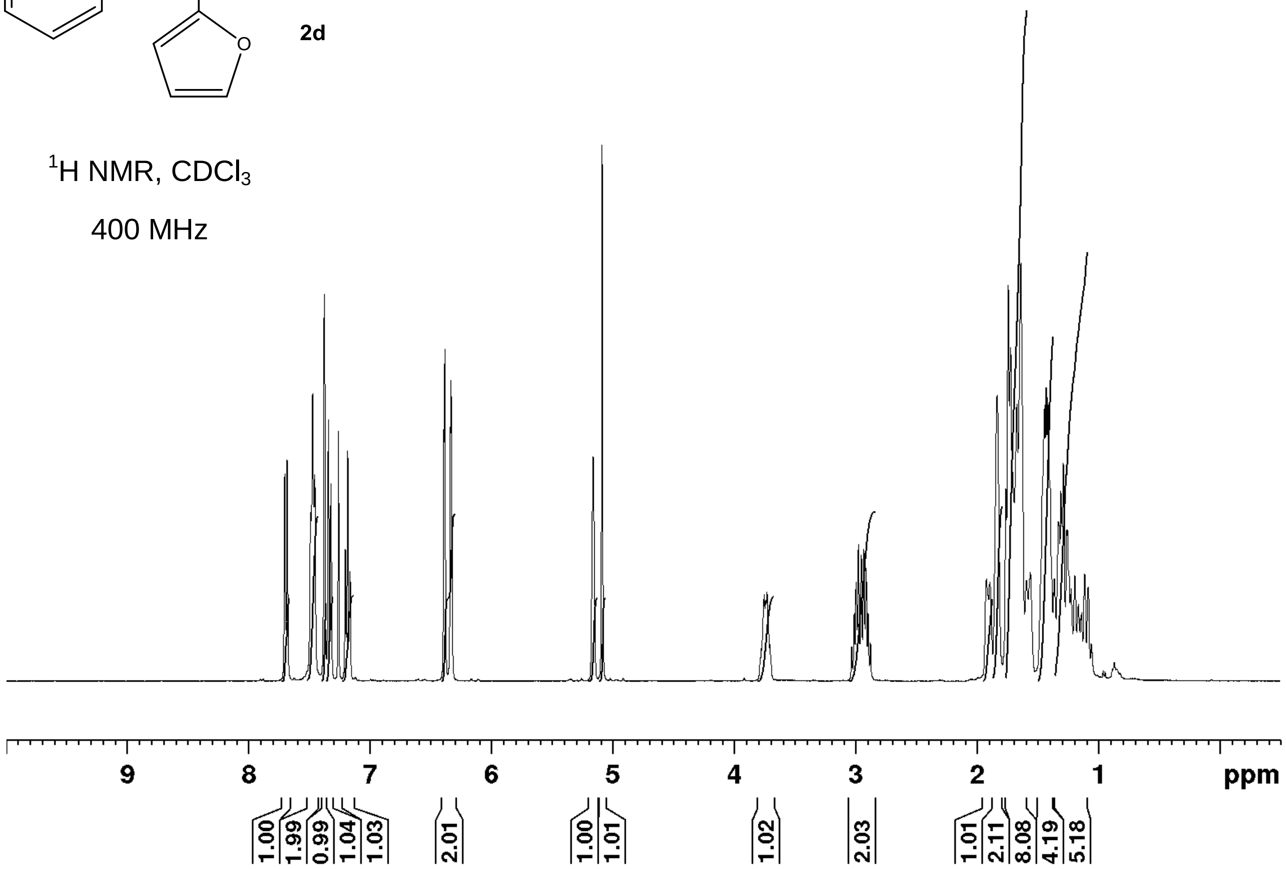


```
Current Data Parameters
NAME              MM-2-64
EXPNO                   1
PROCNO                  1

F2 - Acquisition Parameters
Date_            20160309
Time                11.31
INSTRUM             spect
PROBHD   5 mm PABBO BB/
PULPROG              zg30
TD                  65536
SOLVENT             CDCl3
NS                     16
DS                      2
SWH              8012.820 Hz
FIDRES           0.122266 Hz
AQ              4.0894465 sec
RG                 105.13
DW                 62.400 usec
DE                   6.50 usec
TE                  293.9 K
D1             1.00000000 sec
TD0                     1

======== CHANNEL f1 ========
SFO1          400.1524711 MHz
NUC1                   1H
P1                  15.00 usec
PLW1           9.80000019 W

F2 - Processing parameters
SI                  65536
SF            400.1500112 MHz
WDW                    EM
SSB      0
LB                   0.30 Hz
GB       0
PC                   1.00
```

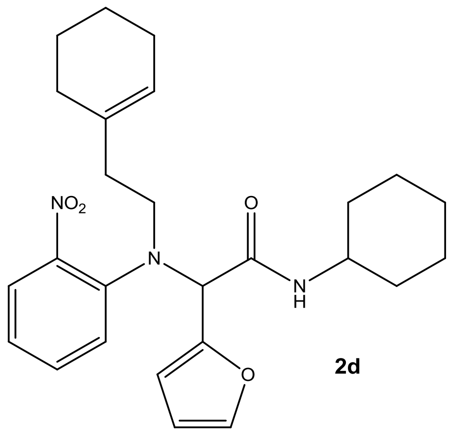

2d

^{13}C NMR, $CDCl_3$

100 MHz

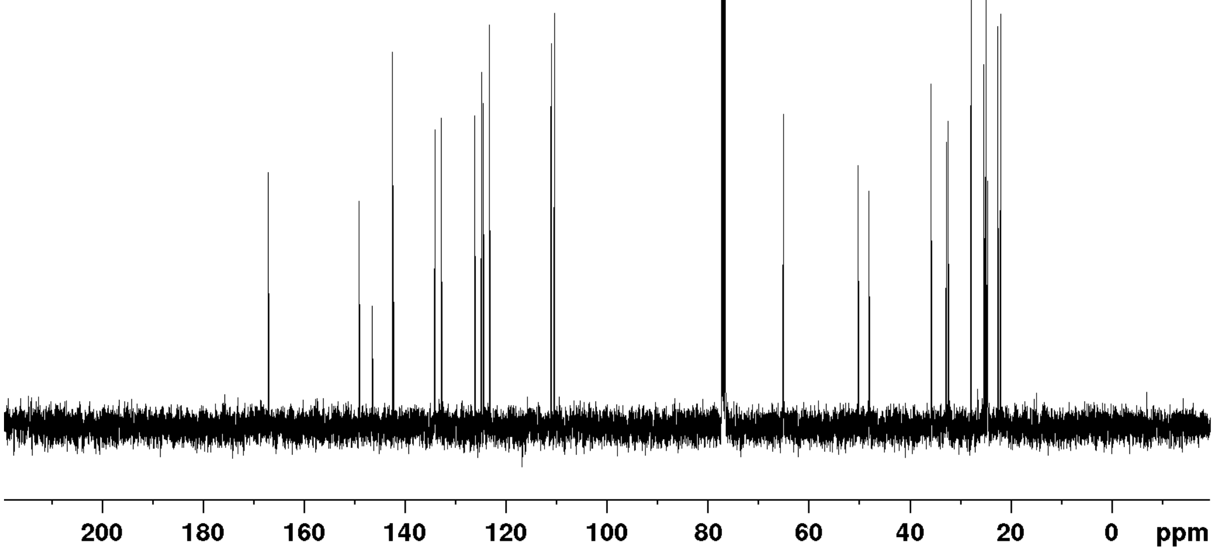

BRUKER

```
Current Data Parameters
NAME                MM-2-64
EXPNO                     2
PROCNO                    1

F2 - Acquisition Parameters
Date_              20160309
Time                  11.40
INSTRUM               spect
PROBHD    5 mm PABBO BB/
PULPROG             zgpg30
TD                    65536
SOLVENT               CDCl3
NS                      128
DS                        4
SWH               24038.461 Hz
FIDRES             0.366798 Hz
AQ                1.3631488 sec
RG                   210.96
DW                   20.800 usec
DE                    10.00 usec
TE                    295.0 K
D1               2.00000000 sec
D11              0.03000000 sec
TD0                       1

======== CHANNEL f1 ========
SFO1            100.6278588 MHz
NUC1                    13C
P1                    10.00 usec
PLW1            48.20000076 W

======== CHANNEL f2 ========
SFO2            400.1516006 MHz
NUC2                     1H
CPDPRG[2            waltz16
PCPD2                 90.00 usec
PLW2             9.80000019 W
PLW12            0.27221999 W
PLW13            0.22050001 W

F2 - Processing parameters
SI                    32768
SF              100.6177941 MHz
WDW                      EM
SSB      0
LB                     1.00 Hz
GB       0
PC                     1.40
```

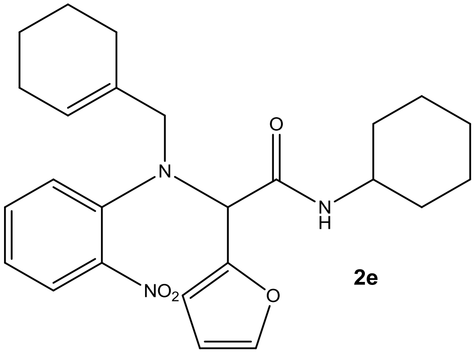

2e

^1H NMR, $CDCl_3$

400 MHz

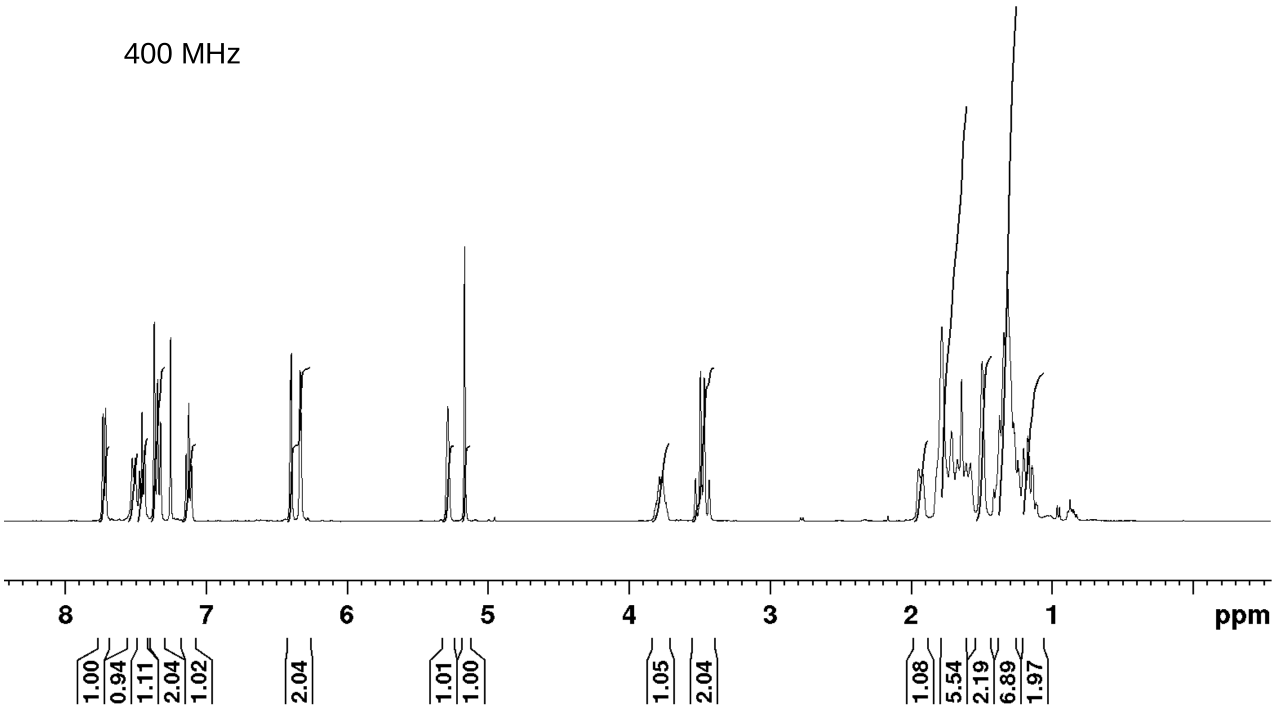


```
Current Data Parameters
NAME               MM-2-79
EXPNO                    1
PROCNO                   1

F2 - Acquisition Parameters
Date_             20160308
Time                 15.17
INSTRUM              spect
PROBHD     5 mm PABBO BB/
PULPROG               zg30
TD                   65536
SOLVENT              CDCl3
NS                      16
DS                       2
SWH               8012.820 Hz
FIDRES            0.122266 Hz
AQ               4.0894465 sec
RG                   134.4
DW                  62.400 usec
DE                    6.50 usec
TE                   293.9 K
D1              1.00000000 sec
TD0                      1

======== CHANNEL f1 ========
SFO1          400.1524711 MHz
NUC1                   1H
P1                  15.00 usec
PLW1           9.80000019 W

F2 - Processing parameters
SI                   65536
SF             400.1500119 MHz
WDW                     EM
SSB        0
LB                    0.30 Hz
GB         0
PC                    1.00
```

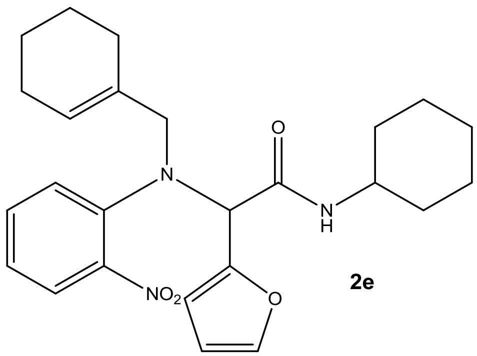

2e

^{13}C NMR, $CDCl_3$

100 MHz

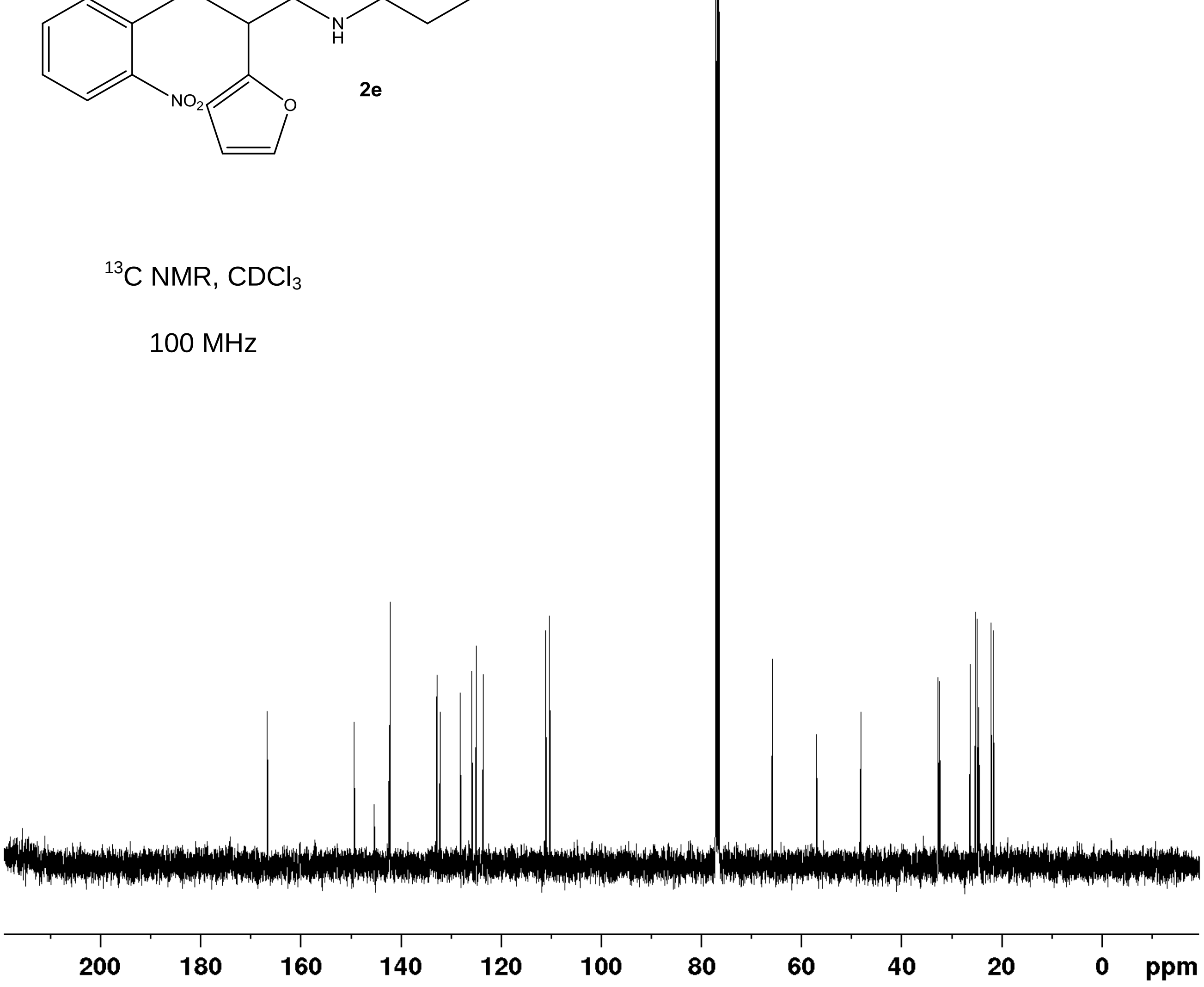


```
Current Data Parameters
NAME              MM-2-79
EXPNO                   2
PROCNO                  1

F2 - Acquisition Parameters
Date_            20160308
Time                15.26
INSTRUM             spect
PROBHD     5 mm PABBO BB/
PULPROG           zgpg30
TD                 65536
SOLVENT            CDCl3
NS                   128
DS                     4
SWH            24038.461 Hz
FIDRES          0.366798 Hz
AQ             1.3631488 sec
RG                210.96
DW                20.800 usec
DE                 10.00 usec
TE                 294.9 K
D1            2.00000000 sec
D11           0.03000000 sec
TD0                    1

======== CHANNEL f1 ========
SFO1         100.6278588 MHz
NUC1                 13C
P1                 10.00 usec
PLW1         48.20000076 W

======== CHANNEL f2 ========
SFO2         400.1516006 MHz
NUC2                  1H
CPDPRG[2         waltz16
PCPD2              90.00 usec
PLW2          9.80000019 W
PLW12         0.27221999 W
PLW13         0.22050001 W

F2 - Processing parameters
SI                 32768
SF           100.6178041 MHz
WDW                   EM
SSB      0
LB                  1.00 Hz
GB       0
PC                  1.40
```

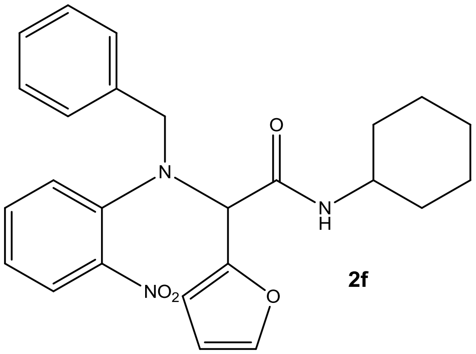

2f

^{1}H NMR, $CDCl_3$

400 MHz

```
Current Data Parameters
NAME             a4 good
EXPNO                  1
PROCNO                 1

F2 - Acquisition Parameters
Date_           20160710
Time               14.36
INSTRUM            spect
PROBHD   5 mm PABBO BB/
PULPROG             zg30
TD                 65536
SOLVENT            CDCl3
NS                    16
DS                     2
SWH             8012.820 Hz
FIDRES          0.122266 Hz
AQ             4.0894465 sec
RG                 134.4
DW                62.400 usec
DE                  6.50 usec
TE                 294.2 K
D1            1.00000000 sec
TD0                    1

======== CHANNEL f1 ========
SFO1         400.1524711 MHz
NUC1                  1H
P1                 15.00 usec
PLW1          9.80000019 W

F2 - Processing parameters
SI                 65536
SF           400.1500106 MHz
WDW                   EM
SSB      0
LB                  0.30 Hz
GB       0
PC                  1.00
```

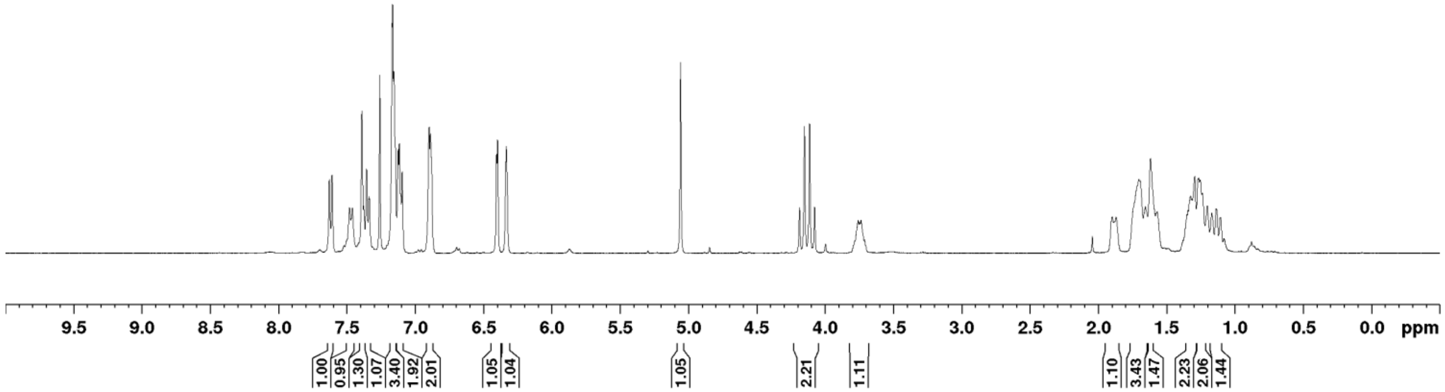

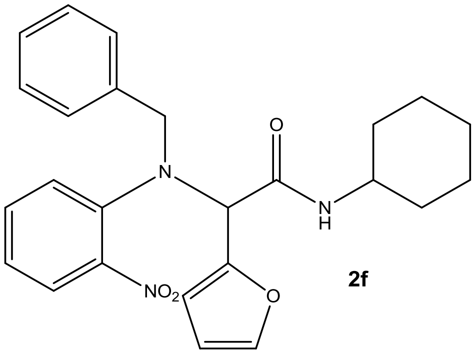

2f

^{13}C NMR, $CDCl_3$

100 MHz

```
Current Data Parameters
NAME            a4 carbon
EXPNO                   1
PROCNO                  1

F2   Acquisition Parameters
Date_            20160710
Time                23.39
INSTRUM             spect
PROBHD   5 mm PABBO BB/
PULPROG           zgpg30
TD                 65536
SOLVENT            CDCl3
NS                   256
DS                     4
SWH            24038.461 Hz
FIDRES          0.366798 Hz
AQ             1.3631488 sec
RG                210.96
DW                20.800 usec
DE                 10.00 usec
TE                 295.4 K
D1            2.00000000 sec
D11           0.03000000 sec
TD0                    1

======== CHANNEL f1 ========
SFO1         100.6278588 MHz
NUC1                 13C
P1                 10.00 usec
PLW1         48.20000076 W

======== CHANNEL f2 ========
SFO2         400.1516006 MHz
NUC2                  1H
CPDPRG[2         waltz16
PCPD2              90.00 usec
PLW2          9.80000019 W
PLW12         0.27221999 W
PLW13         0.22050001 W

F2   Processing parameters
SI                 32768
SF           100.6177879 MHz
WDW                   EM
SSB      0
LB                  1.00 Hz
GB       0
PC                  1.40
```

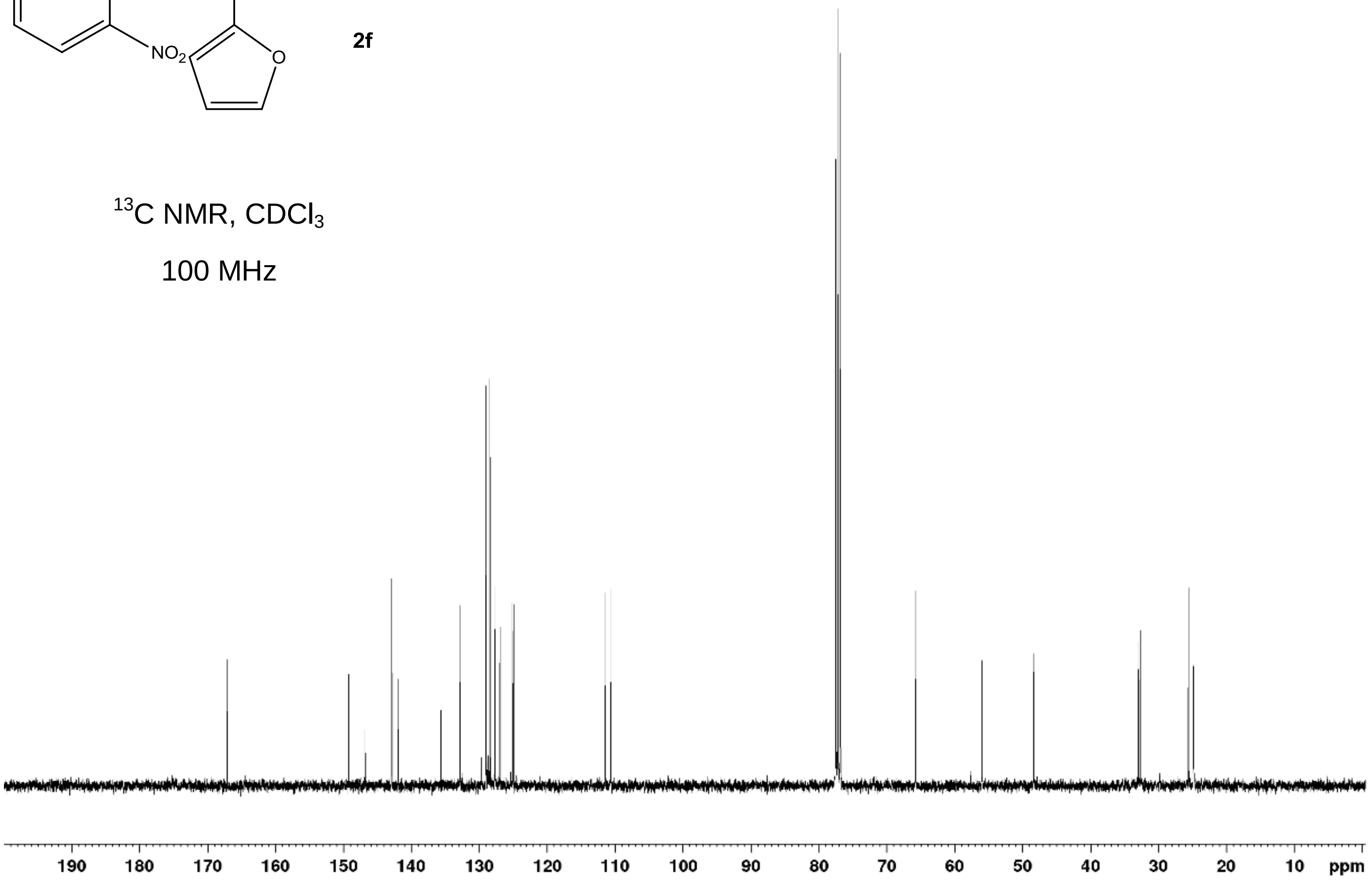

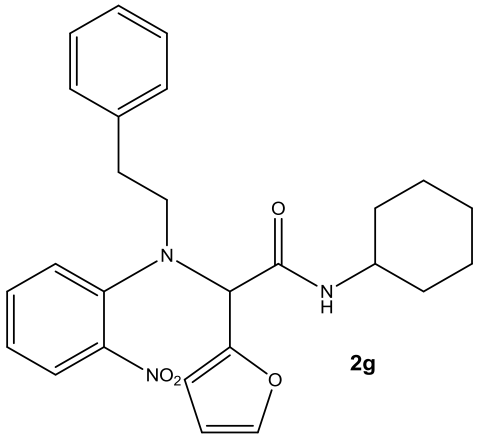

2g

^{1}H NMR, $CDCl_3$

400 MHz

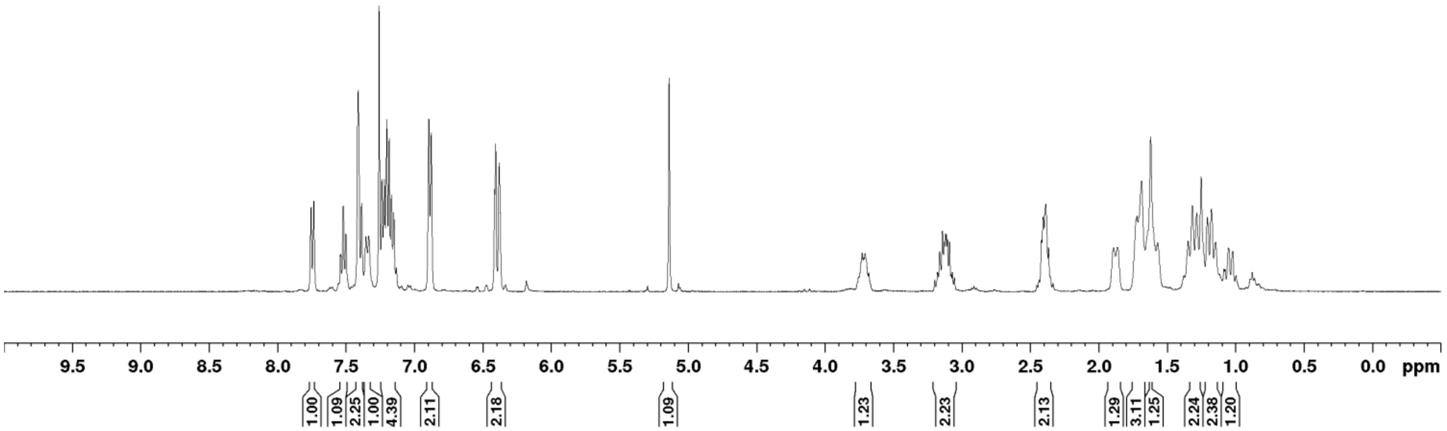

BRUKER

```
Current Data Parameters
NAME              a1 good
EXPNO                   1
PROCNO                  1

F2 - Acquisition Parameters
Date_            20160710
Time                14.31
INSTRUM             spect
PROBHD   5 mm PABBO BB/
PULPROG              zg30
TD                  65536
SOLVENT             CDCl3
NS                     16
DS                      2
SWH              8012.820 Hz
FIDRES           0.122266 Hz
AQ              4.0894465 sec
RG                  134.4
DW                 62.400 usec
DE                   6.50 usec
TE                  294.2 K
D1             1.00000000 sec
TD0                     1

======== CHANNEL f1 ========
SFO1         400.1524711 MHz
NUC1                   1H
P1                  15.00 usec
PLW1           9.80000019 W

F2 - Processing parameters
SI                  65536
SF          400.1500109 MHz
WDW                    EM
SSB      0
LB                   0.30 Hz
GB       0
PC                   1.00
```

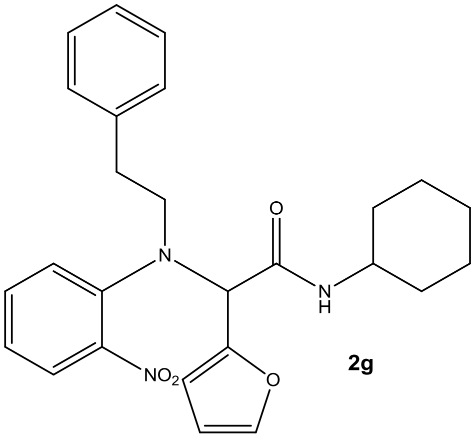

2g

^{13}C NMR, $CDCl_3$

100 MHz

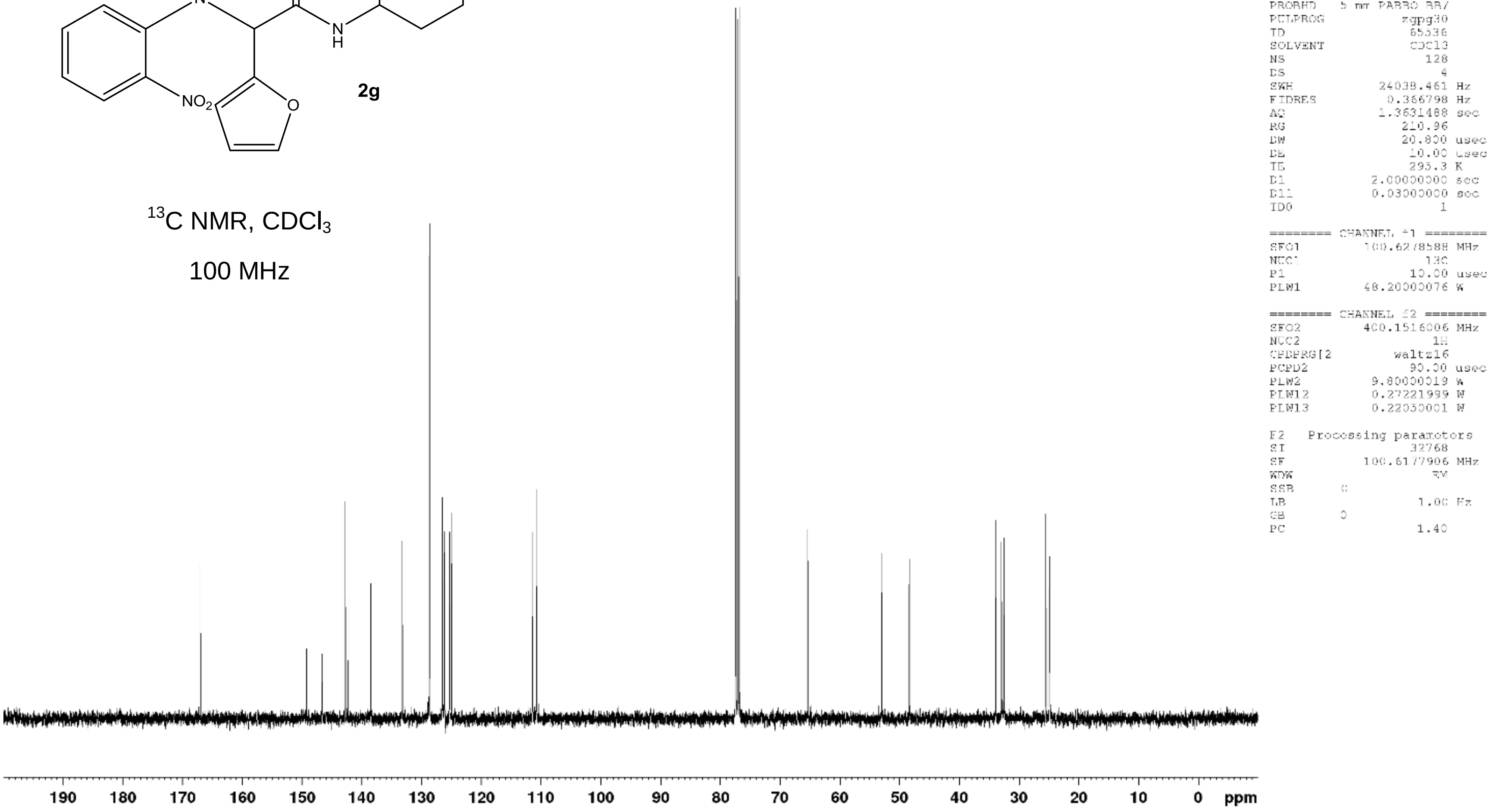

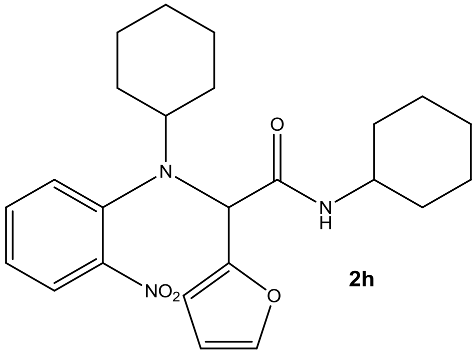

2h

^{1}H NMR, $CDCl_3$

400 MHz

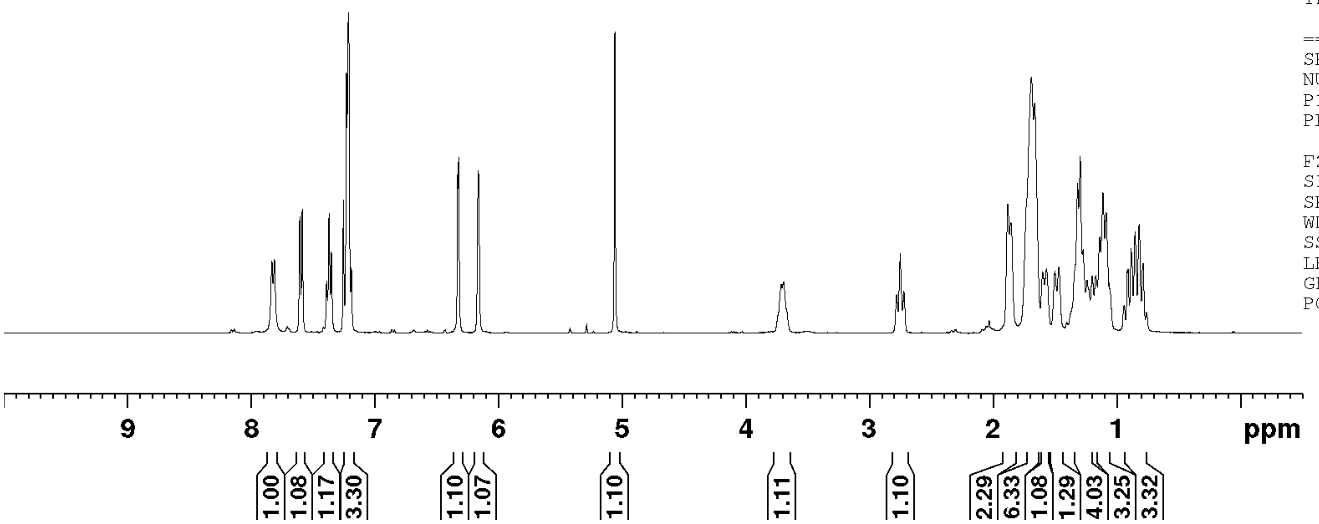


```
Current Data Parameters
NAME              A2-Better
EXPNO                     2
PROCNO                    1

F2 - Acquisition Parameters
Date_              20160711
Time                  18.57
INSTRUM               spect
PROBHD    5 mm PABBO BB/
PULPROG                zg30
TD                    65536
SOLVENT               CDCl3
NS                       16
DS                        2
SWH            8012.820 Hz
FIDRES         0.122266 Hz
AQ            4.0894465 sec
RG                   105.13
DW             62.400 usec
DE               6.50 usec
TE                  293.8 K
D1          1.00000000 sec
TD0                       1

======== CHANNEL f1 ========
SFO1       400.1524711 MHz
NUC1                     1H
P1               15.00 usec
PLW1         9.80000019 W

F2 - Processing parameters
SI                    65536
SF         400.1500136 MHz
WDW                      EM
SSB      0
LB                  0.30 Hz
GB       0
PC                     1.00
```

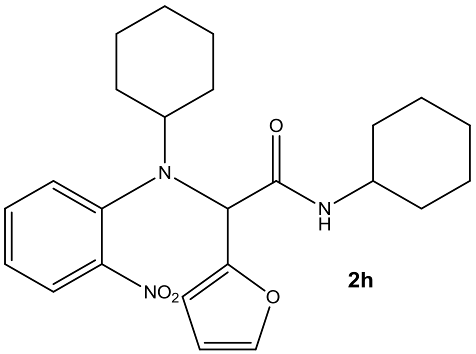

2h

^{13}C NMR, $CDCl_3$

100 MHz

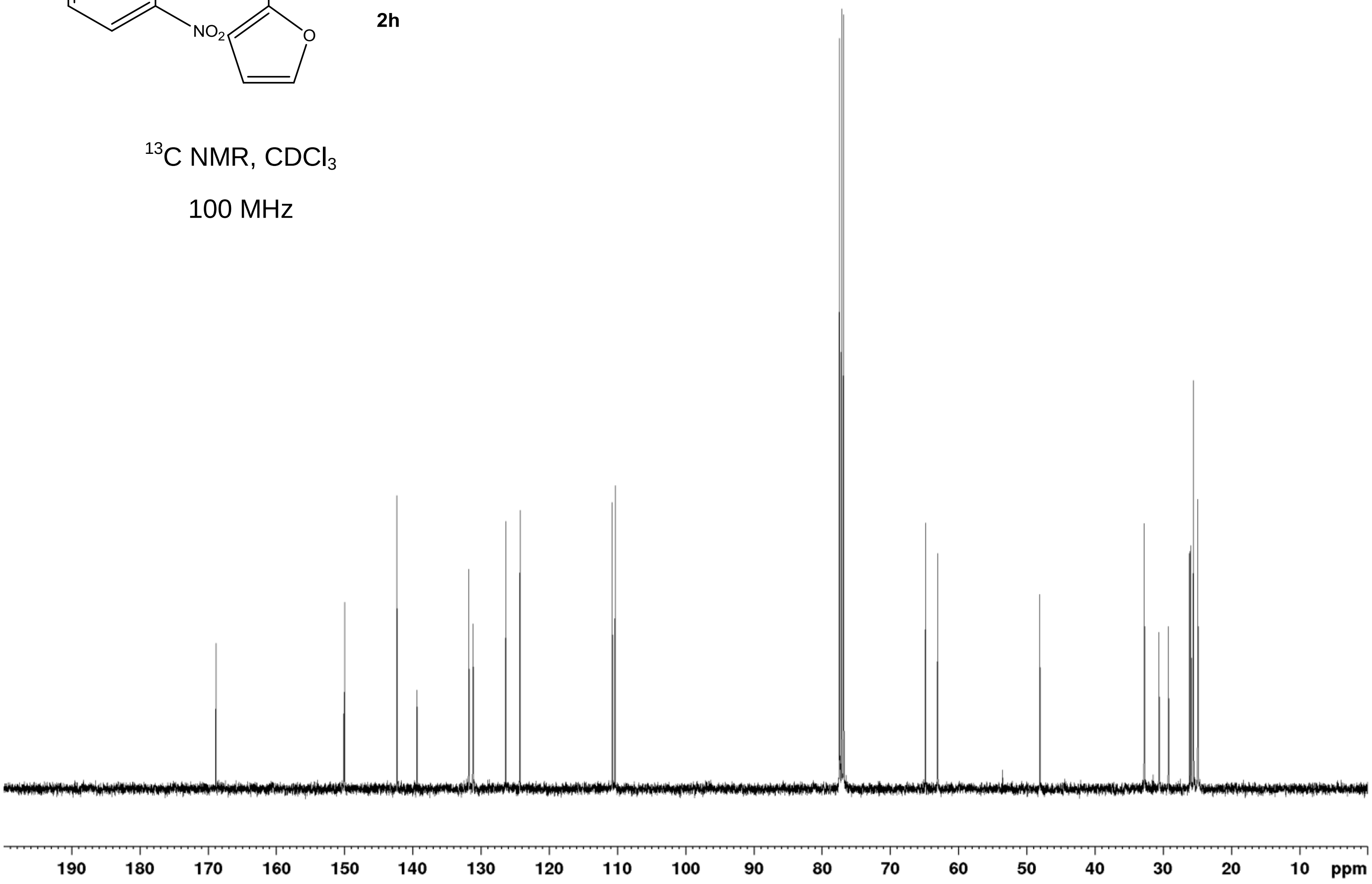


```
Current Data Parameters
NAME            a2 carbon
EXPNO                   1
PROCNO                  1

F2 - Acquisition Parameters
Date_            20160710
Time                21.40
INSTRUM             spect
PROBHD    5 mm PABBO BB/
PULPROG           zgpg30
TD                 65536
SOLVENT            CDCl3
NS                   256
DS                     4
SWH            24038.461 Hz
FIDRES          0.366798 Hz
AQ             1.3631488 sec
RG                210.96
DW                20.800 usec
DE                 10.00 usec
TE                 295.2 K
D1            2.00000000 sec
D11           0.03000000 sec
TD0                    1

======== CHANNEL f1 ========
SFO1         100.6278588 MHz
NUC1                 13C
P1                 10.00 usec
PLW1         48.20000076 W

======== CHANNEL f2 ========
SFO2         400.1516006 MHz
NUC2                  1H
CPDPRG[2         waltz16
PCPD2              90.00 usec
PLW2          9.80000019 W
PLW12         0.27221999 W
PLW13         0.22050001 W

F2 - Processing parameters
SI                 32768
SF           100.6177898 MHz
WDW                   EM
SSB      0
LB                  1.00 Hz
GB       0
PC                  1.40
```

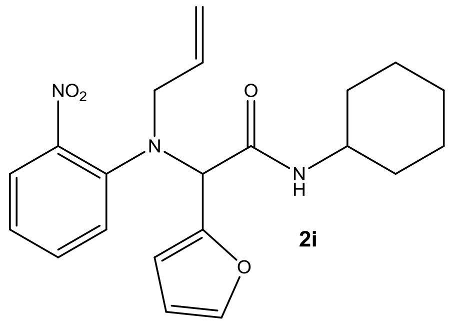

2i

^{1}H NMR, $CDCl_3$

400 MHz

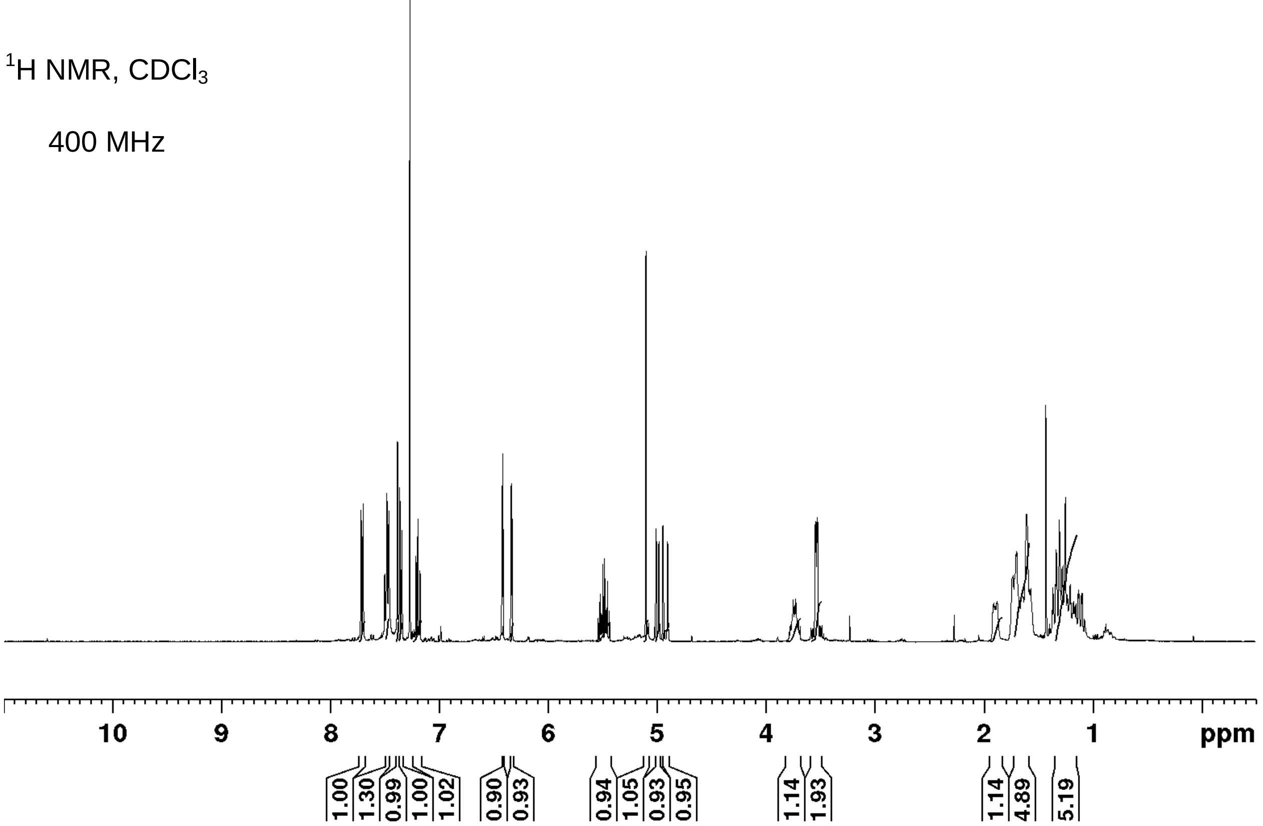


```
Current Data Parameters
NAME      MM-2-6Hr-F10-14
EXPNO                   1
PROCNO                  1

F2 - Acquisition Parameters
Date_            20160129
Time                18.48
INSTRUM             spect
PROBHD   5 mm PABBO BB/
PULPROG              zg30
TD                  65536
SOLVENT             CDCl3
NS                     16
DS                      2
SWH              8012.820 Hz
FIDRES           0.122266 Hz
AQ              4.0894465 sec
RG                 166.66
DW                 62.400 usec
DE                   6.50 usec
TE                  293.4 K
D1             1.00000000 sec
TD0                     1

======== CHANNEL f1 ========
SFO1          400.1524711 MHz
NUC1                   1H
P1                  15.00 usec
PLW1           9.80000019 W

F2 - Processing parameters
SI                  65536
SF            400.1500082 MHz
WDW                    EM
SSB      0
LB                   0.30 Hz
GB       0
PC                   1.00
```

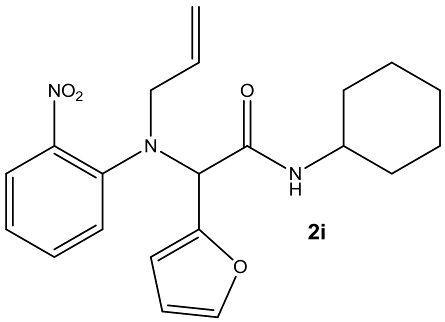

2i

^{13}C NMR, $CDCl_3$

100 MHz

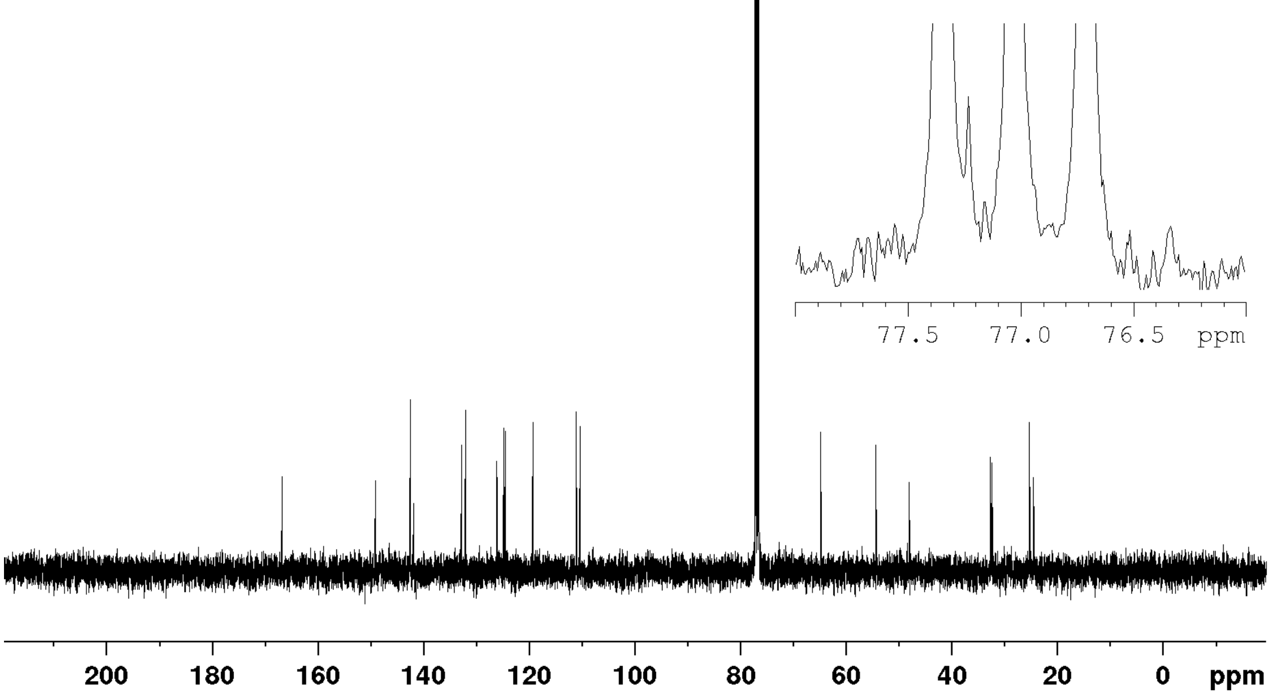


```
Current Data Parameters
NAME      MM-2-6Hr-F10-14
EXPNO                   3
PROCNO                  1

F2 - Acquisition Parameters
Date_            20160129
Time                19.28
INSTRUM             spect
PROBHD    5 mm PABBO BB/
PULPROG           zgpg30
TD                 65536
SOLVENT            CDCl3
NS                   256
DS                     4
SWH            24038.461 Hz
FIDRES          0.366798 Hz
AQ             1.3631488 sec
RG                210.96
DW                20.800 usec
DE                 10.00 usec
TE                 294.7 K
D1            2.00000000 sec
D11           0.03000000 sec
TD0                    1

======== CHANNEL f1 ========
SFO1         100.6278588 MHz
NUC1                 13C
P1                 10.00 usec
PLW1         48.20000076 W

======== CHANNEL f2 ========
SFO2         400.1516006 MHz
NUC2                  1H
CPDPRG[2         waltz16
PCPD2              90.00 usec
PLW2          9.80000019 W
PLW12         0.27221999 W
PLW13         0.22050001 W

F2 - Processing parameters
SI                 32768
SF           100.6177980 MHz
WDW                   EM
SSB      0
LB                  1.00 Hz
GB       0
PC                  1.40
```

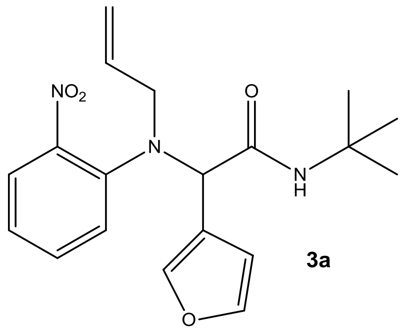

3a

^{1}H NMR, $CDCl_3$

400 MHz

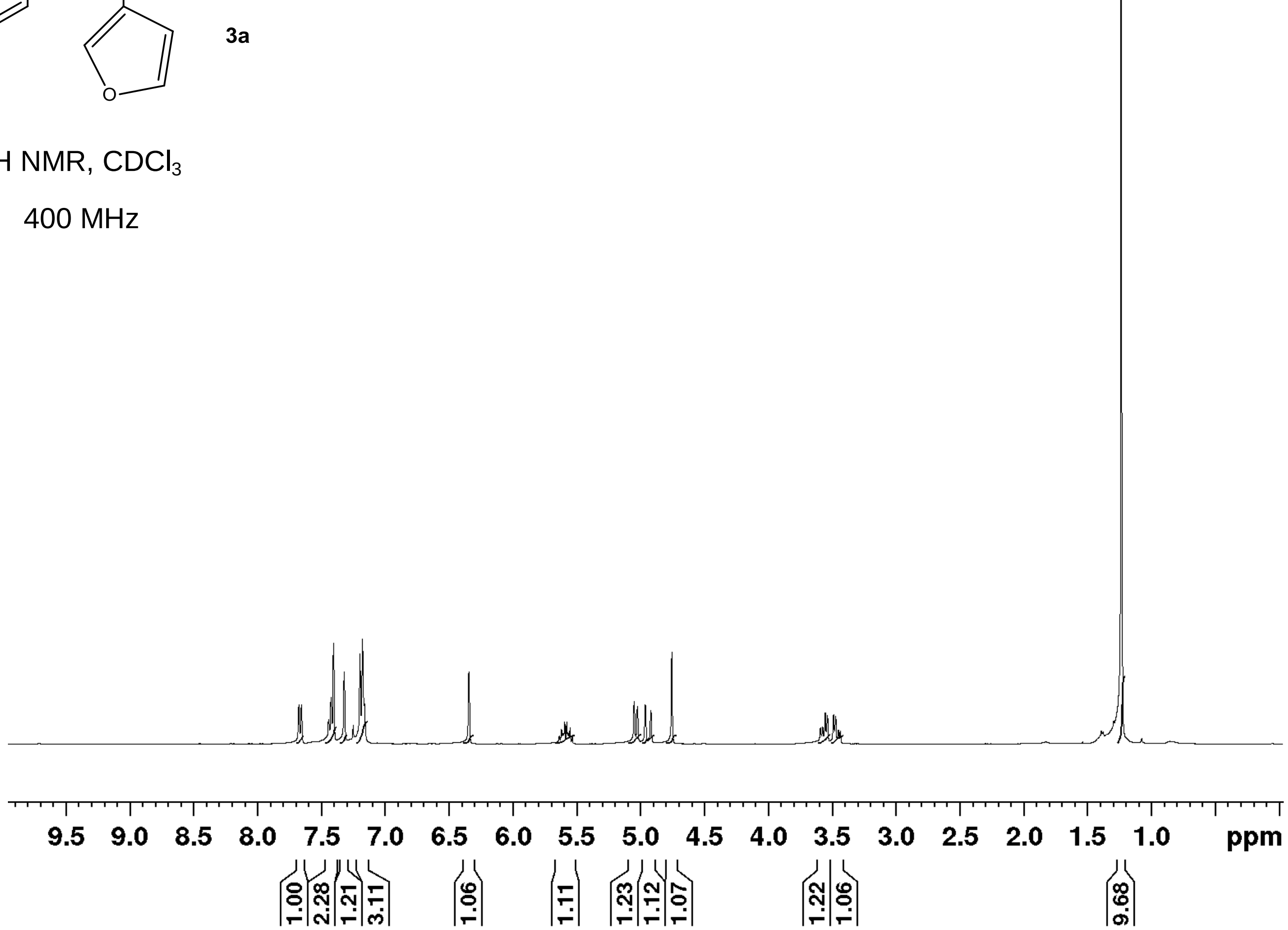

BRUKER

```
Current Data Parameters
NAME          KM-11-9-14R-F9-16
EXPNO                        10
PROCNO                        1

F2 - Acquisition Parameters
Date_              20150105
Time                  12.07
INSTRUM               spect
PROBHD   5 mm PABBO BB/
PULPROG                zg30
TD                    65536
SOLVENT               CDCl3
NS                       16
DS                        2
SWH                8012.820 Hz
FIDRES             0.122266 Hz
AQ                4.0894465 sec
RG                    57.34
DW                   62.400 usec
DE                     6.50 usec
TE                    292.9 K
D1               1.00000000 sec
TD0                       1

======== CHANNEL f1 ========
SFO1            400.1524711 MHz
NUC1                     1H
P1                    15.00 usec
PLW1             9.80000019 W

F2 - Processing parameters
SI                    65536
SF              400.1500149 MHz
WDW                      EM
SSB      0
LB                     0.30 Hz
GB       0
PC                     1.00
```

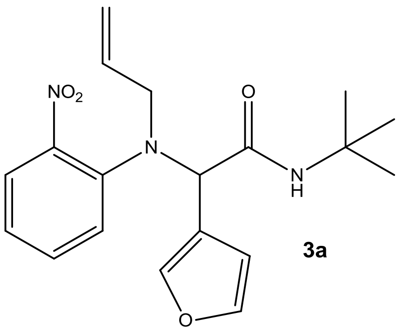

3a

^{13}C NMR, $CDCl_3$

100 MHz

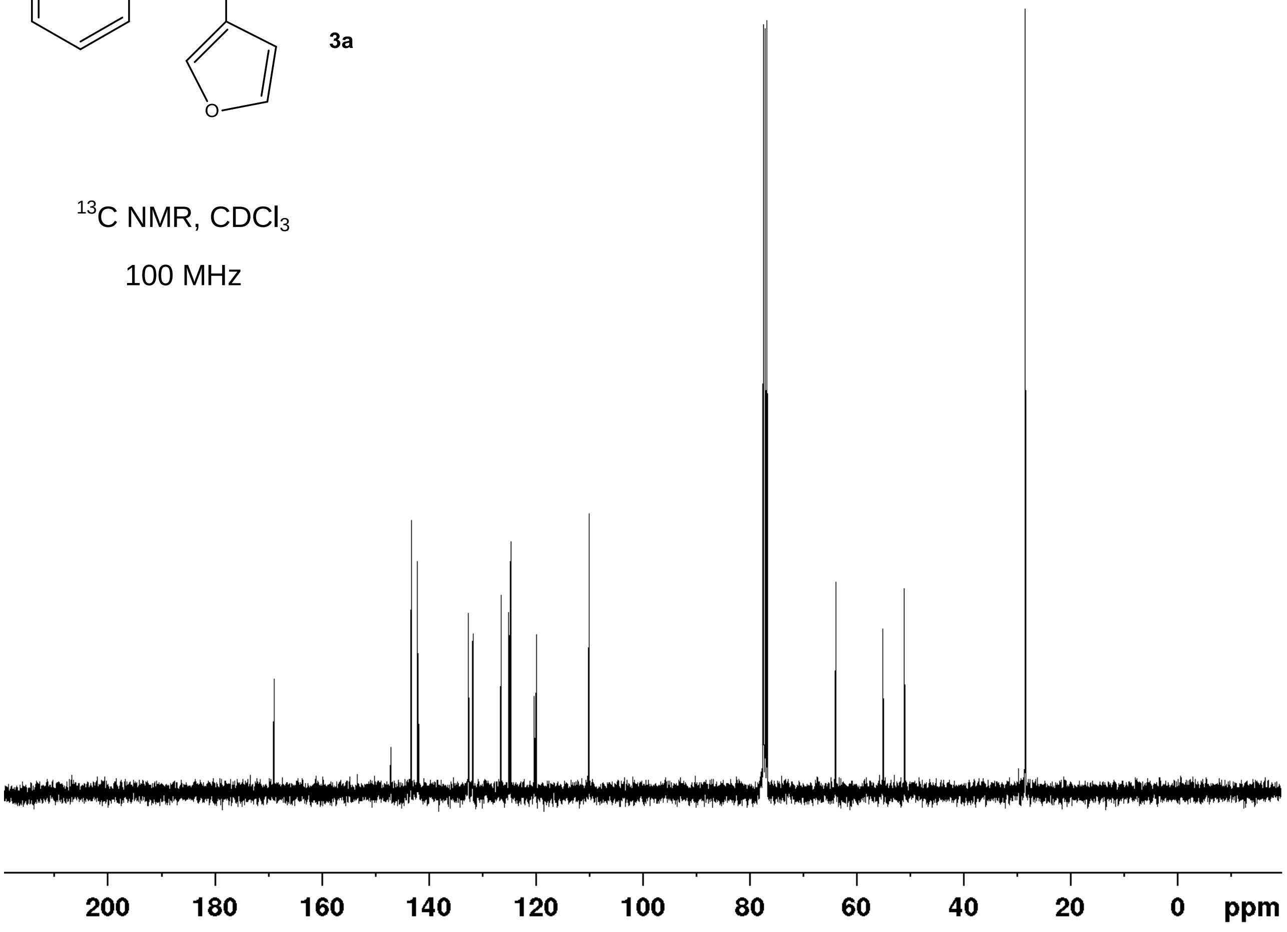


```
Current Data Parameters
NAME      KM-11-9-14R-F9-16-C
EXPNO                   10
PROCNO                   1

F2 - Acquisition Parameters
Date_             20150106
Time                 14.05
INSTRUM              spect
PROBHD    5 mm PABBO BB/
PULPROG            zgpg30
TD                  65536
SOLVENT             CDCl3
NS                    128
DS                      4
SWH             24038.461 Hz
FIDRES           0.366798 Hz
AQ              1.3631488 sec
RG                 210.96
DW                 20.800 usec
DE                  10.00 usec
TE                  294.3 K
D1             2.00000000 sec
D11            0.03000000 sec
TD0                     1

======== CHANNEL f1 ========
SFO1        100.6278588 MHz
NUC1                 13C
P1                 10.00 usec
PLW1         48.20000076 W

======== CHANNEL f2 ========
SFO2        400.1516006 MHz
NUC2                  1H
CPDPRG[2         waltz16
PCPD2              90.00 usec
PLW2          9.80000019 W
PLW12         0.27221999 W
PLW13         0.22050001 W

F2 - Processing parameters
SI                 32768
SF          100.6177971 MHz
WDW                   EM
SSB       0
LB                  1.00 Hz
GB        0
PC                  1.40
```

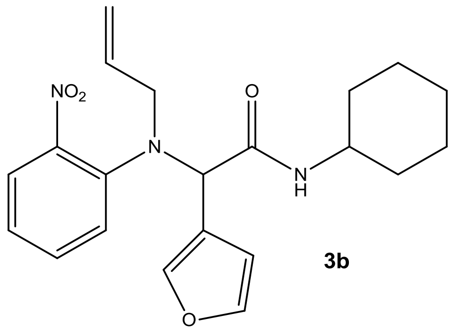

3b

^{1}H NMR, $CDCl_3$

400 MHz

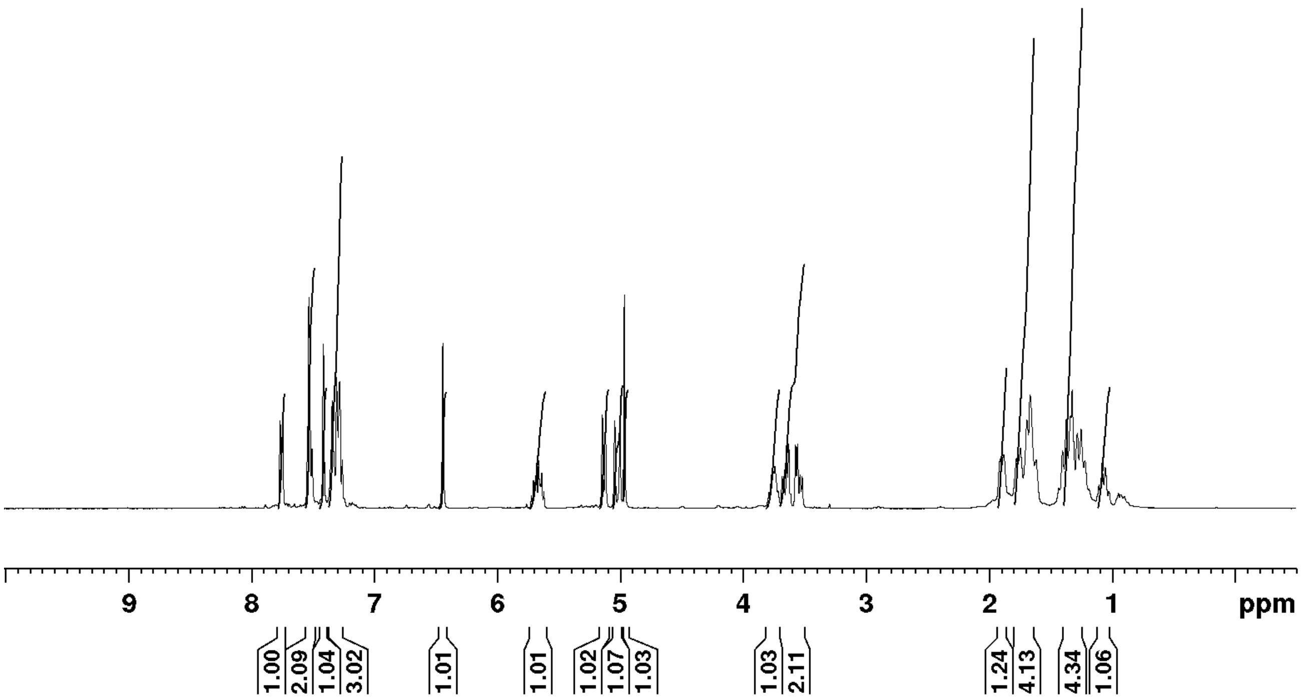

Current Data Parameters
NAME MM-2-71
EXPNO 1
PROCNO 1

F2 - Acquisition Parameters
Date_ 20160307
Time 20.42
INSTRUM spect
PROBHD 5 mm PABBO BB/
PULPROG zg30
TD 65536
SOLVENT CDCl3
NS 16
DS 2
SWH 8012.820 Hz
FIDRES 0.122266 Hz
AQ 4.0894465 sec
RG 51.73
DW 62.400 usec
DE 6.50 usec
TE 296.3 K
D1 1.00000000 sec
TD0 1

======== CHANNEL f1 ========
SFO1 400.1524711 MHz
NUC1 1H
P1 15.00 usec
PLW1 9.80000019 W

F2 - Processing parameters
SI 65536
SF 400.1499781 MHz
WDW EM
SSB 0
LB 0.30 Hz
GB 0
PC 1.00

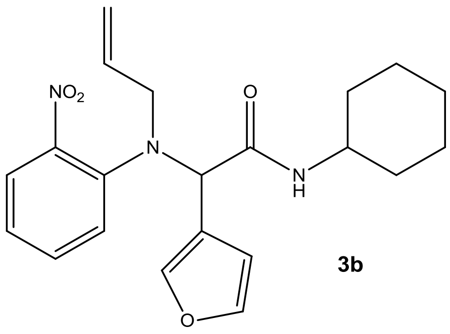

3b

^{13}C NMR, $CDCl_3$

100 MHz

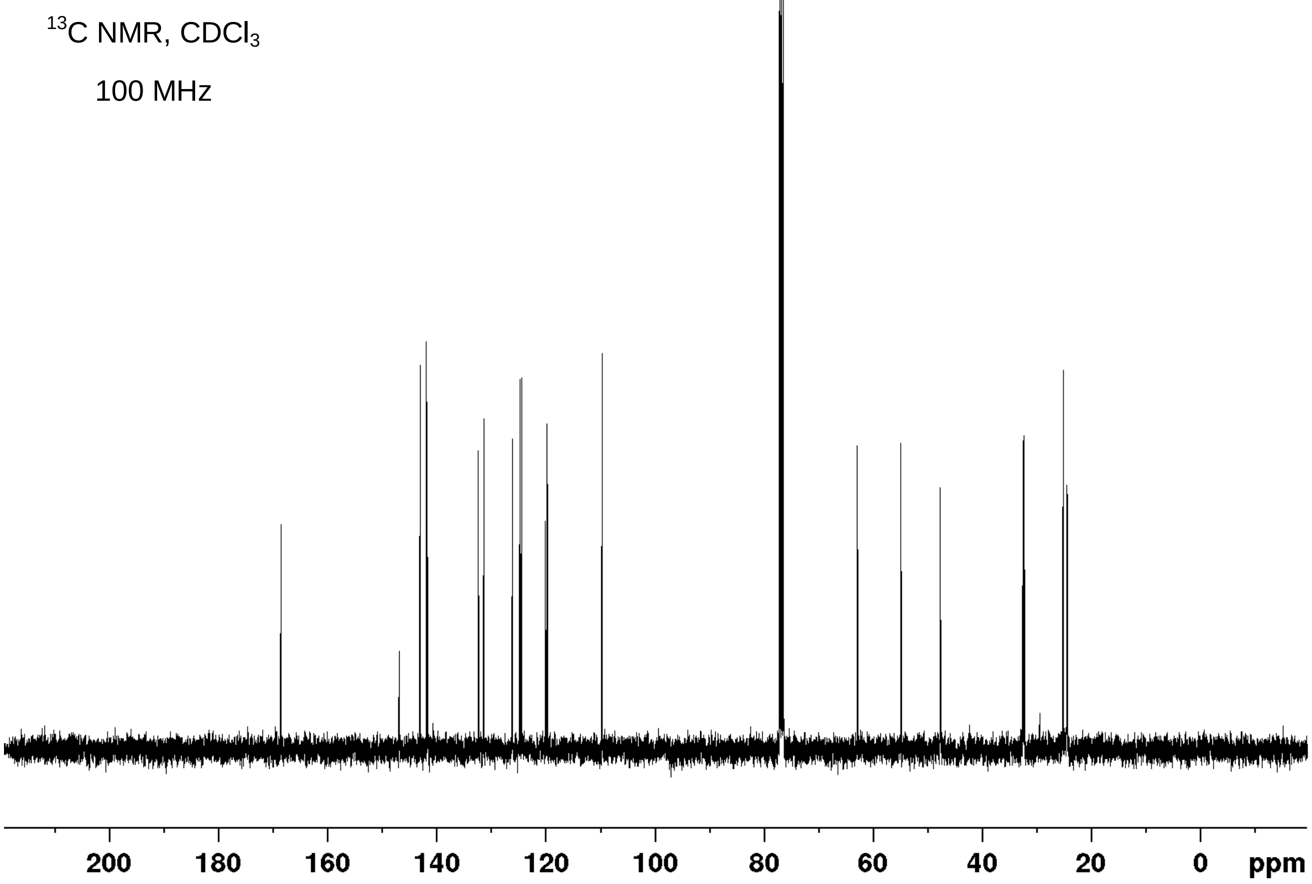


```
Current Data Parameters
NAME                MM-2-71
EXPNO                     2
PROCNO                    1

F2 - Acquisition Parameters
Date_              20160307
Time                  20.51
INSTRUM               spect
PROBHD    5 mm PABBO BB/
PULPROG             zgpg30
TD                    65536
SOLVENT               CDCl3
NS                      128
DS                        4
SWH           24038.461 Hz
FIDRES         0.366798 Hz
AQ            1.3631488 sec
RG                   210.96
DW               20.800 usec
DE                10.00 usec
TE                  297.2 K
D1           2.00000000 sec
D11          0.03000000 sec
TD0                       1

======== CHANNEL f1 ========
SFO1        100.6278588 MHz
NUC1                    13C
P1                10.00 usec
PLW1        48.20000076 W

======== CHANNEL f2 ========
SFO2        400.1516006 MHz
NUC2                     1H
CPDPRG[2           waltz16
PCPD2             90.00 usec
PLW2         9.80000019 W
PLW12        0.27221999 W
PLW13        0.22050001 W

F2 - Processing parameters
SI                    32768
SF          100.6178041 MHz
WDW                      EM
SSB        0
LB                  1.00 Hz
GB         0
PC                     1.40
```

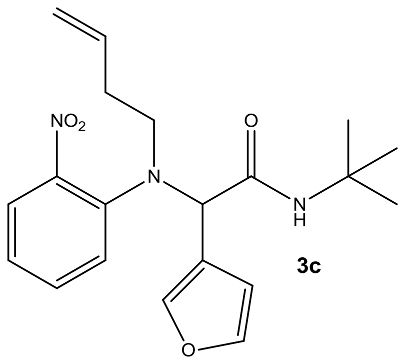

3c

^{1}H NMR, $CDCl_3$

400 MHz

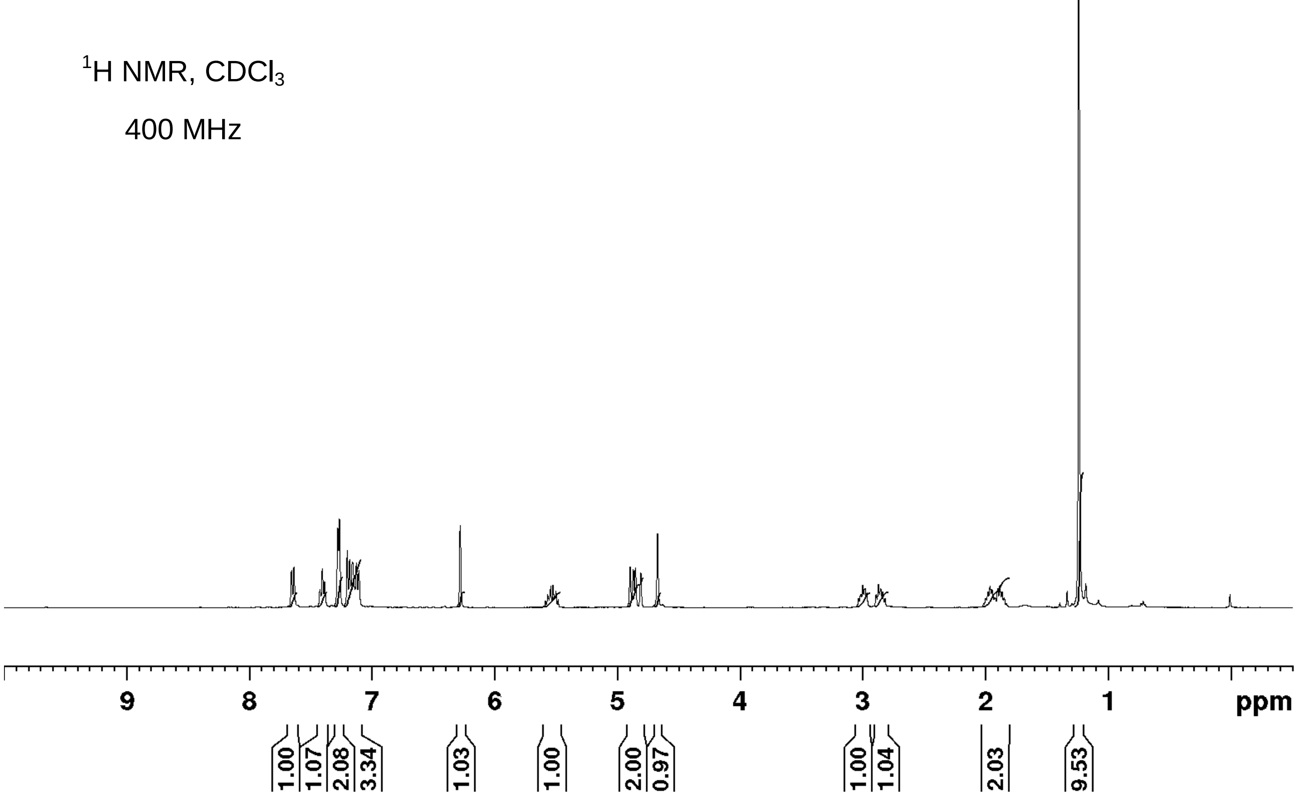

Current Data Parameters
NAME KM-37R-F12-35-3
EXPNO 10
PROCNO 1

F2 - Acquisition Parameters
Date_ 20150513
Time 13.35
INSTRUM spect
PROBHD 5 mm PABBO BB/
PULPROG zg30
TD 65536
SOLVENT CDCl3
NS 16
DS 2
SWH 8012.820 Hz
FIDRES 0.122266 Hz
AQ 4.0894465 sec
RG 95.15
DW 62.400 usec
DE 6.50 usec
TE 292.4 K
D1 1.00000000 sec
TD0 1

======== CHANNEL f1 ========
SFO1 400.1524711 MHz
NUC1 1H
P1 15.00 usec
PLW1 9.80000019 W

F2 - Processing parameters
SI 65536
SF 400.1500353 MHz
WDW EM
SSB 0
LB 0.30 Hz
GB 0
PC 1.00

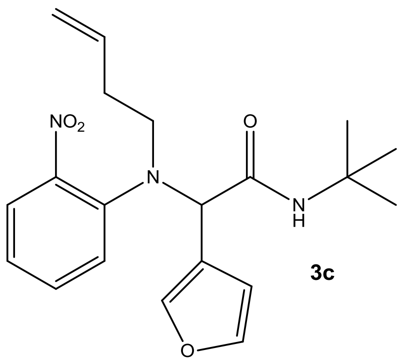

3c

^{13}C NMR, $CDCl_3$

100 MHz

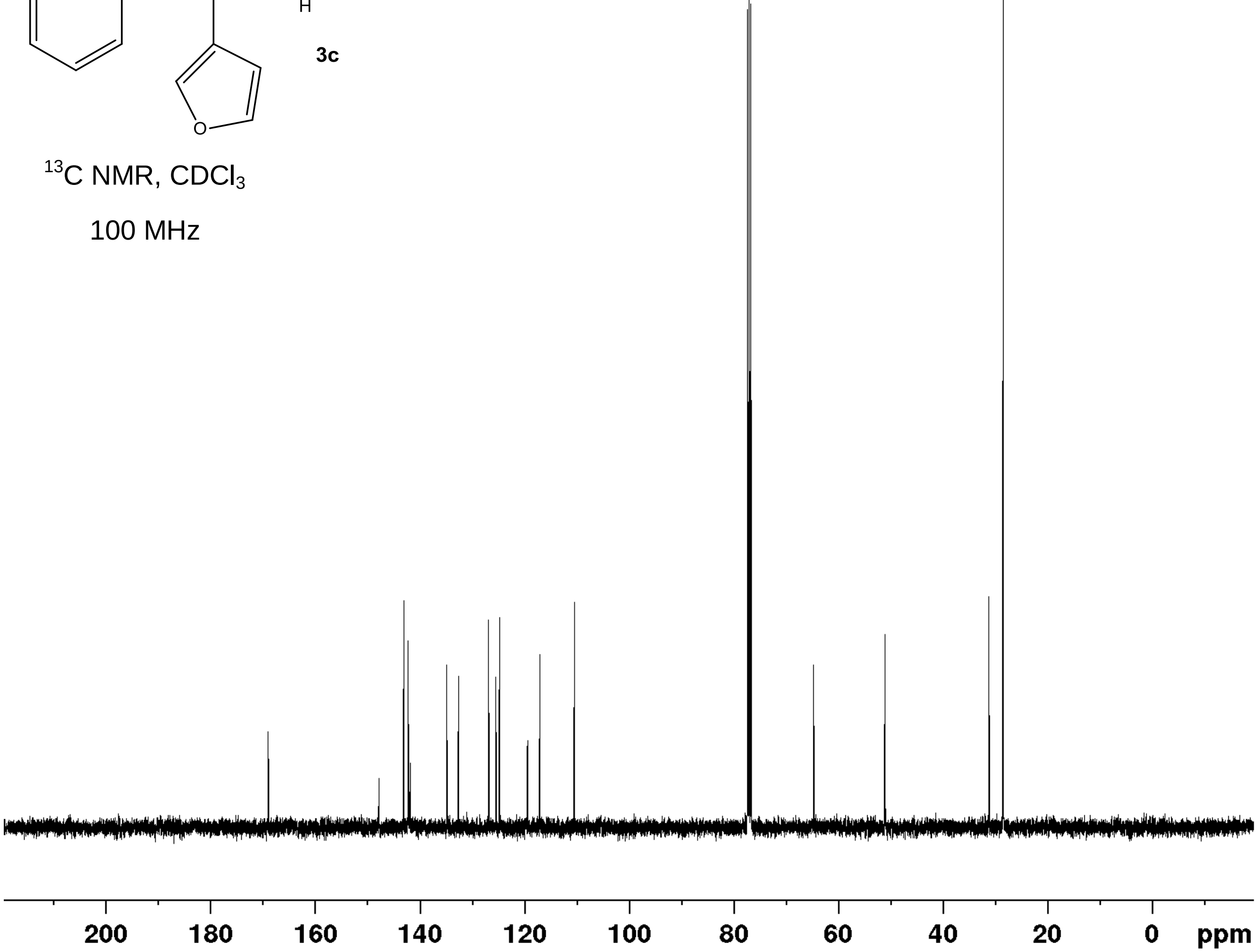

Current Data Parameters
NAME KM-37R-F12-35-3
EXPNO 20
PROCNO 1

F2 - Acquisition Parameters
Date_ 20150513
Time 13.44
INSTRUM spect
PROBHD 5 mm PABBO BB/
PULPROG zgpg30
TD 65536
SOLVENT CDCl3
NS 128
DS 4
SWH 24038.461 Hz
FIDRES 0.366798 Hz
AQ 1.3631488 sec
RG 210.96
DW 20.800 usec
DE 10.00 usec
TE 293.5 K
D1 2.00000000 sec
D11 0.03000000 sec
TD0 1

======== CHANNEL f1 ========
SFO1 100.6278588 MHz
NUC1 13C
P1 10.00 usec
PLW1 48.20000076 W

======== CHANNEL f2 ========
SFO2 400.1516006 MHz
NUC2 1H
CPDPRG[2 waltz16
PCPD2 90.00 usec
PLW2 9.80000019 W
PLW12 0.27221999 W
PLW13 0.22050001 W

F2 - Processing parameters
SI 32768
SF 100.6177903 MHz
WDW EM
SSB 0
LB 1.00 Hz
GB 0
PC 1.40

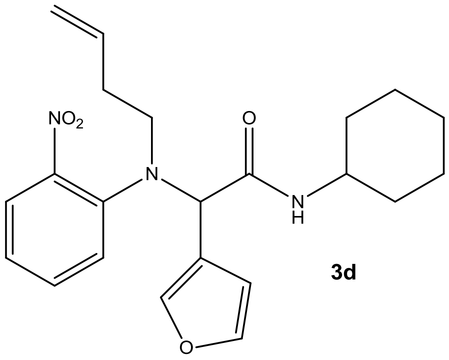

3d

^{1}H NMR, $CDCl_3$

400 MHz

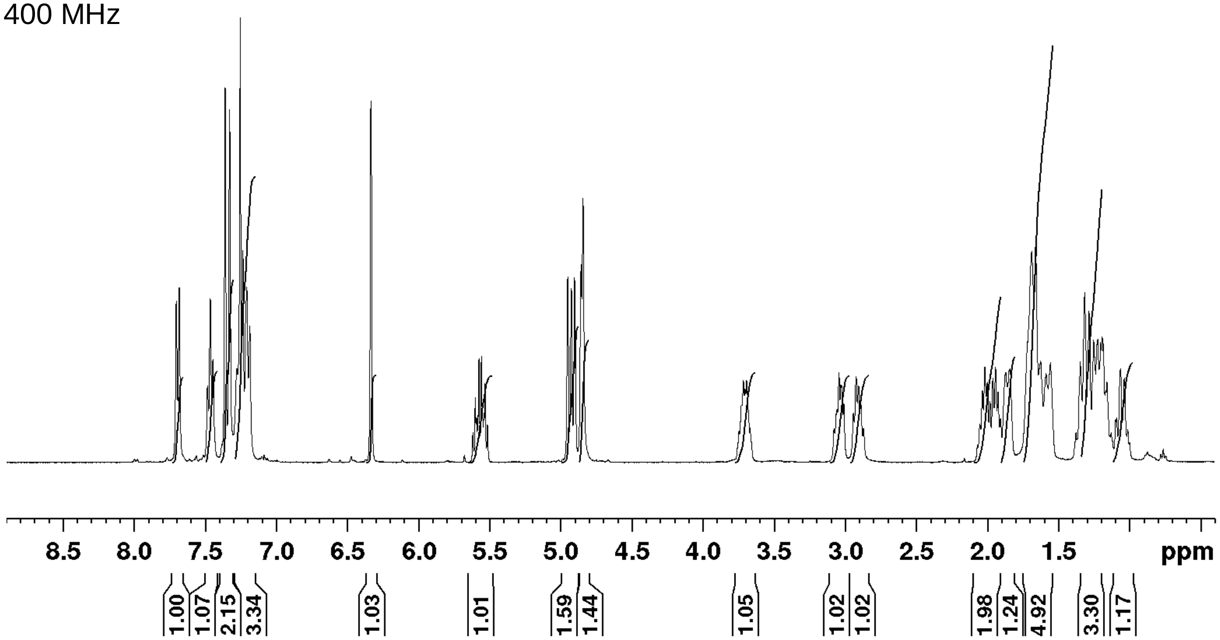

BRUKER

```
Current Data Parameters
NAME              MM-2-73
EXPNO                   3
PROCNO                  1

F2 - Acquisition Parameters
Date_            20160308
Time                15.04
INSTRUM             spect
PROBHD   5 mm PABBO BB/
PULPROG              zg30
TD                  65536
SOLVENT             CDCl3
NS                     16
DS                      2
SWH              8012.820 Hz
FIDRES           0.122266 Hz
AQ              4.0894465 sec
RG                 120.42
DW                 62.400 usec
DE                   6.50 usec
TE                  293.9 K
D1             1.00000000 sec
TD0                     1

======== CHANNEL f1 ========
SFO1          400.1524711 MHz
NUC1                   1H
P1                  15.00 usec
PLW1           9.80000019 W

F2 - Processing parameters
SI                  65536
SF            400.1500119 MHz
WDW                    EM
SSB      0
LB                   0.30 Hz
GB       0
PC                   1.00
```

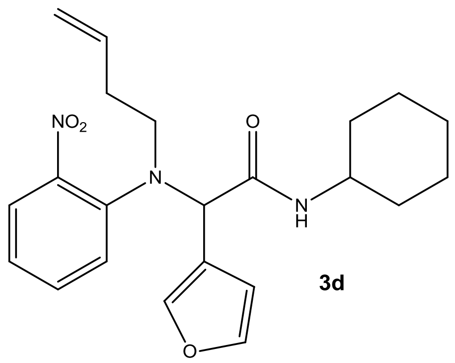

3d

^{13}C NMR, $CDCl_3$

100 MHz

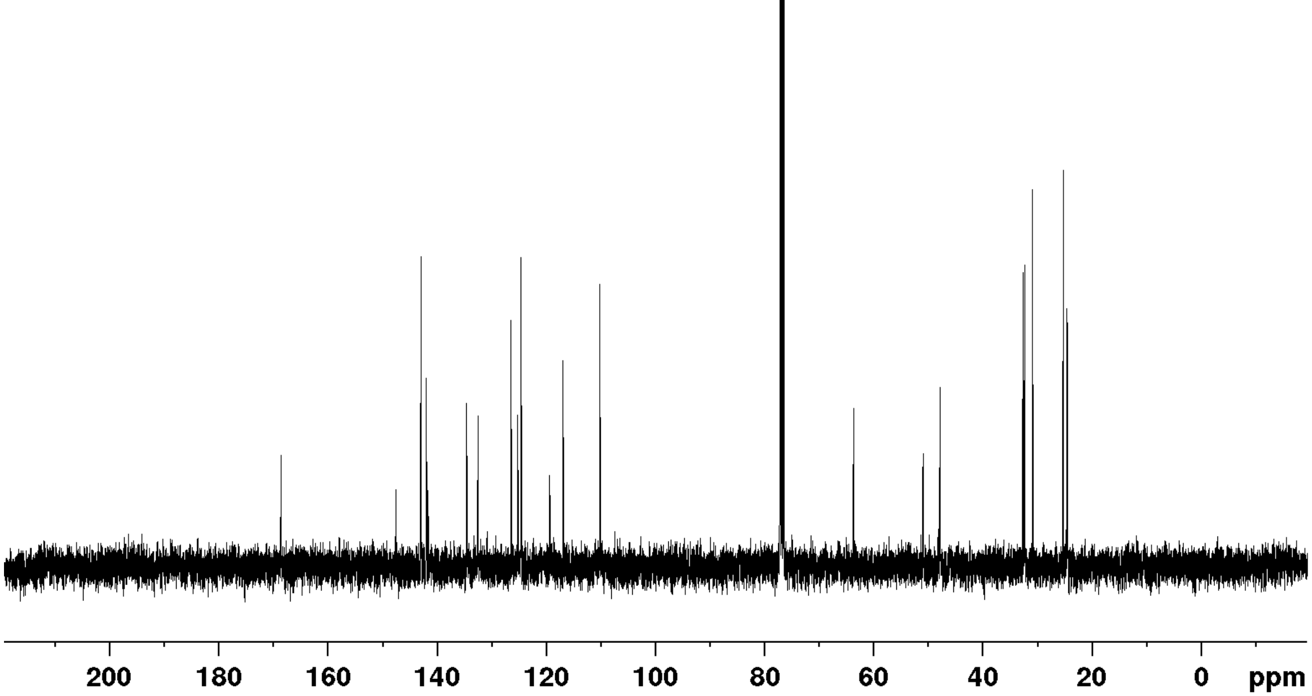


```
Current Data Parameters
NAME              MM-2-73
EXPNO                   4
PROCNO                  1

F2 - Acquisition Parameters
Date_           20160308
Time               15.12
INSTRUM            spect
PROBHD   5 mm PABBO BB/
PULPROG          zgpg30
TD                 65536
SOLVENT            CDCl3
NS                   128
DS                     4
SWH            24038.461 Hz
FIDRES          0.366798 Hz
AQ             1.3631488 sec
RG                210.96
DW                20.800 usec
DE                 10.00 usec
TE                 294.8 K
D1            2.00000000 sec
D11           0.03000000 sec
TD0                    1

======== CHANNEL f1 ========
SFO1         100.6278588 MHz
NUC1                 13C
P1                 10.00 usec
PLW1         48.20000076 W

======== CHANNEL f2 ========
SFO2         400.1516006 MHz
NUC2                  1H
CPDPRG[2         waltz16
PCPD2              90.00 usec
PLW2          9.80000019 W
PLW12         0.27221999 W
PLW13         0.22050001 W

F2 - Processing parameters
SI                 32768
SF           100.6178041 MHz
WDW                   EM
SSB      0
LB                  1.00 Hz
GB       0
PC                  1.40
```

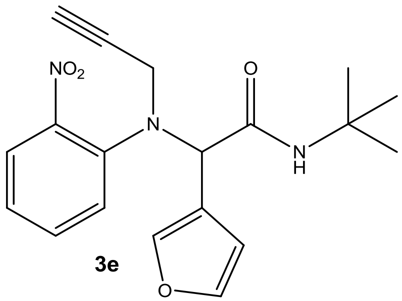

3e

^1H NMR, $CDCl_3$

400 MHz

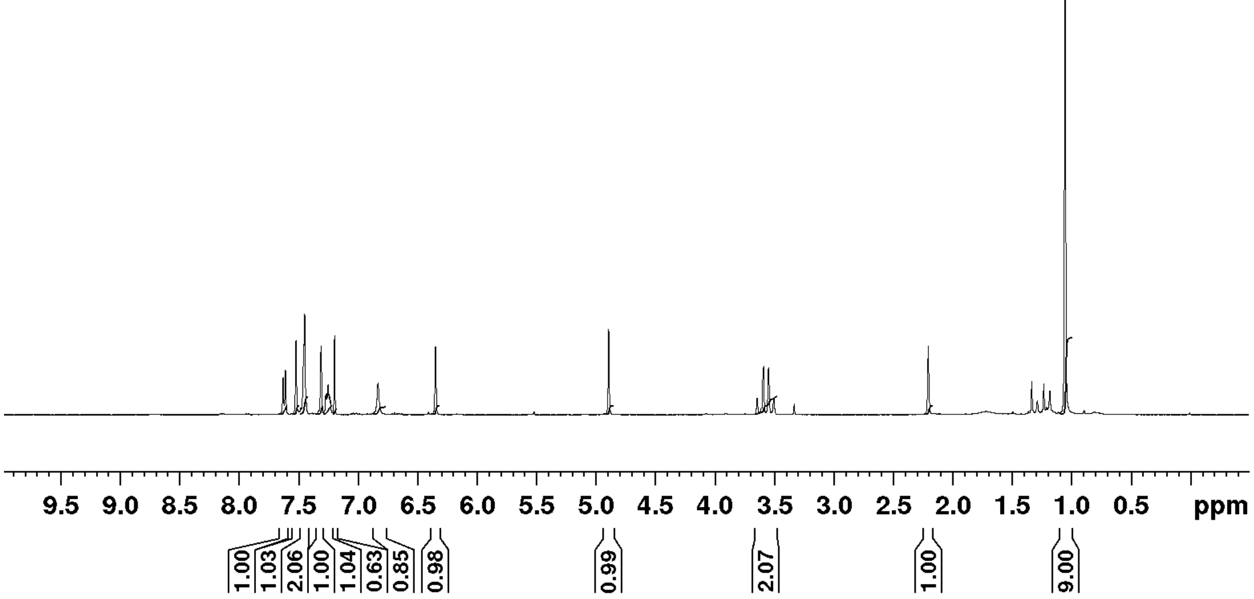


```
Current Data Parameters
NAME      KM-36R-F61-70-3
EXPNO                  10
PROCNO                  1

F2 - Acquisition Parameters
Date_            20150513
Time                13.49
INSTRUM             spect
PROBHD    5 mm PABBO BB/
PULPROG              zg30
TD                  65536
SOLVENT             CDCl3
NS                     16
DS                      2
SWH              8012.820 Hz
FIDRES           0.122266 Hz
AQ              4.0894465 sec
RG                 152.01
DW                 62.400 usec
DE                   6.50 usec
TE                  292.6 K
D1             1.00000000 sec
TD0                     1

======== CHANNEL f1 ========
SFO1          400.1524711 MHz
NUC1                   1H
P1                  15.00 usec
PLW1           9.80000019 W

F2 - Processing parameters
SI                  65536
SF            400.1500364 MHz
WDW                    EM
SSB      0
LB                   0.30 Hz
GB       0
PC                   1.00
```

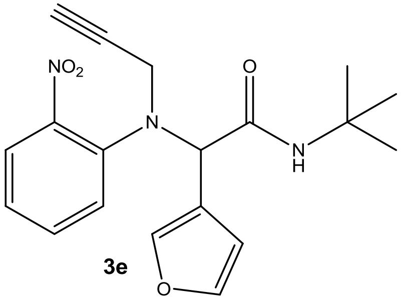

3e

^{13}C NMR, $CDCl_3$

100 MHz

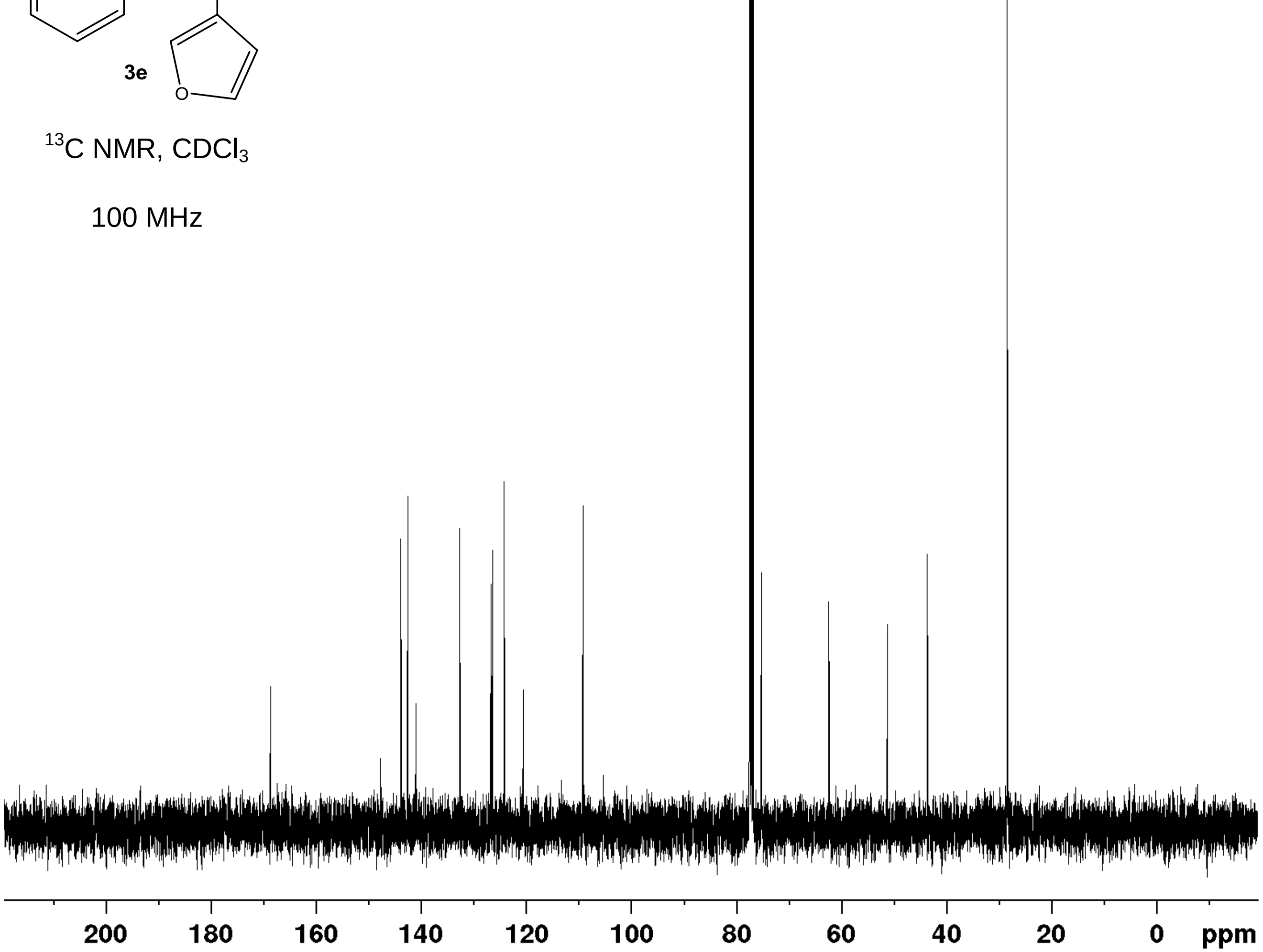


```
Current Data Parameters
NAME      KM-36R-F61-70-3
EXPNO                  20
PROCNO                  1

F2 - Acquisition Parameters
Date_            20150513
Time                13.57
INSTRUM             spect
PROBHD   5 mm PABBO BB/
PULPROG           zgpg30
TD                 65536
SOLVENT            CDCl3
NS                   128
DS                     4
SWH           24038.461 Hz
FIDRES         0.366798 Hz
AQ            1.3631488 sec
RG               210.96
DW               20.800 usec
DE                10.00 usec
TE                293.5 K
D1           2.00000000 sec
D11          0.03000000 sec
TD0                    1

======== CHANNEL f1 ========
SFO1        100.6278588 MHz
NUC1                 13C
P1                 10.00 usec
PLW1         48.20000076 W

======== CHANNEL f2 ========
SFO2        400.1516006 MHz
NUC2                  1H
CPDPRG[2          waltz16
PCPD2              90.00 usec
PLW2          9.80000019 W
PLW12         0.27221999 W
PLW13         0.22050001 W

F2 - Processing parameters
SI                 32768
SF           100.6177852 MHz
WDW                   EM
SSB      0
LB                  1.00 Hz
GB       0
PC                  1.40
```

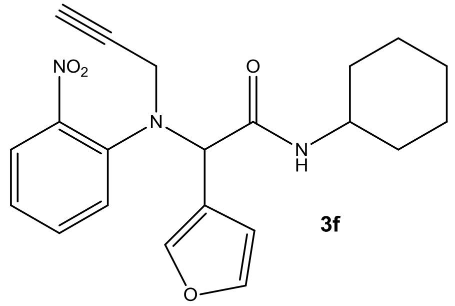

3f

^{1}H NMR, $CDCl_3$

400 MHz

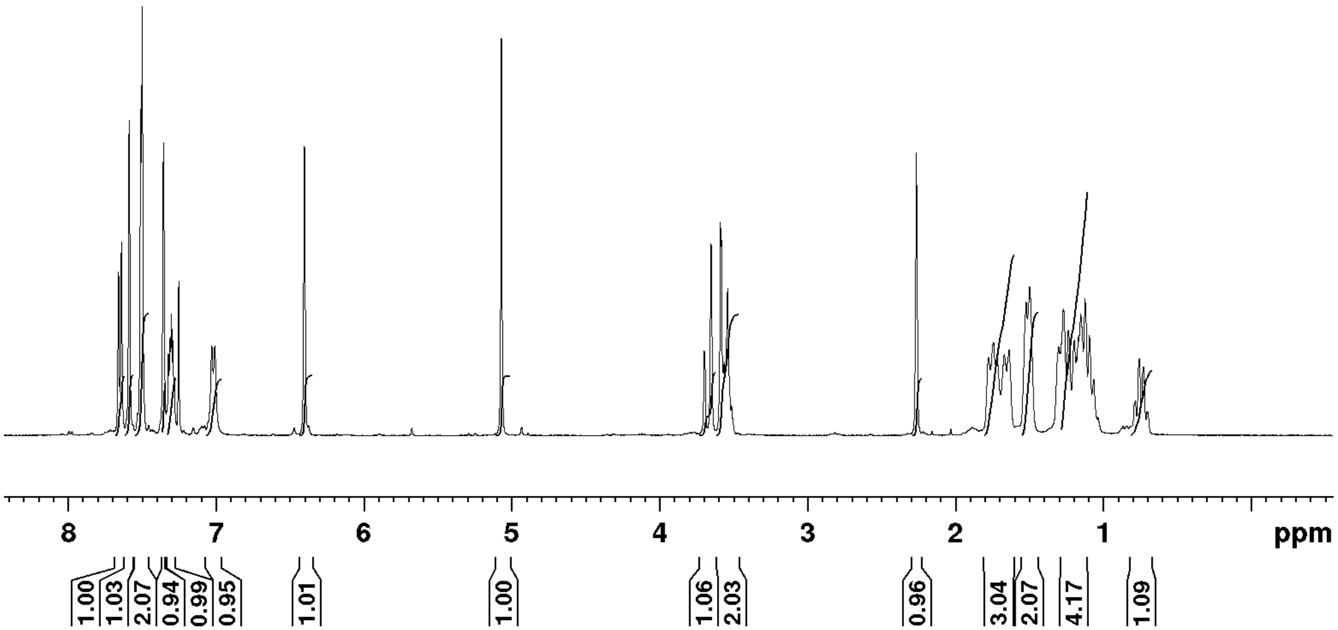


```
Current Data Parameters
NAME                MM-2-72
EXPNO                     3
PROCNO                    1

F2 - Acquisition Parameters
Date_              20160308
Time                  14.50
INSTRUM               spect
PROBHD   5 mm PABBO BB/
PULPROG                zg30
TD                    65536
SOLVENT               CDCl3
NS                       16
DS                        2
SWH                8012.820 Hz
FIDRES             0.122266 Hz
AQ                4.0894465 sec
RG                    57.34
DW                   62.400 usec
DE                     6.50 usec
TE                    293.8 K
D1               1.00000000 sec
TD0                       1

======== CHANNEL f1 ========
SFO1        400.1524711 MHz
NUC1                     1H
P1                    15.00 usec
PLW1             9.80000019 W

F2 - Processing parameters
SI                    65536
SF              400.1500119 MHz
WDW                      EM
SSB      0
LB                     0.30 Hz
GB       0
PC                     1.00
```

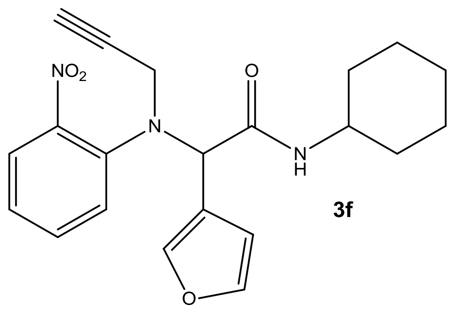

3f

^{13}C NMR, $CDCl_3$

100 MHz

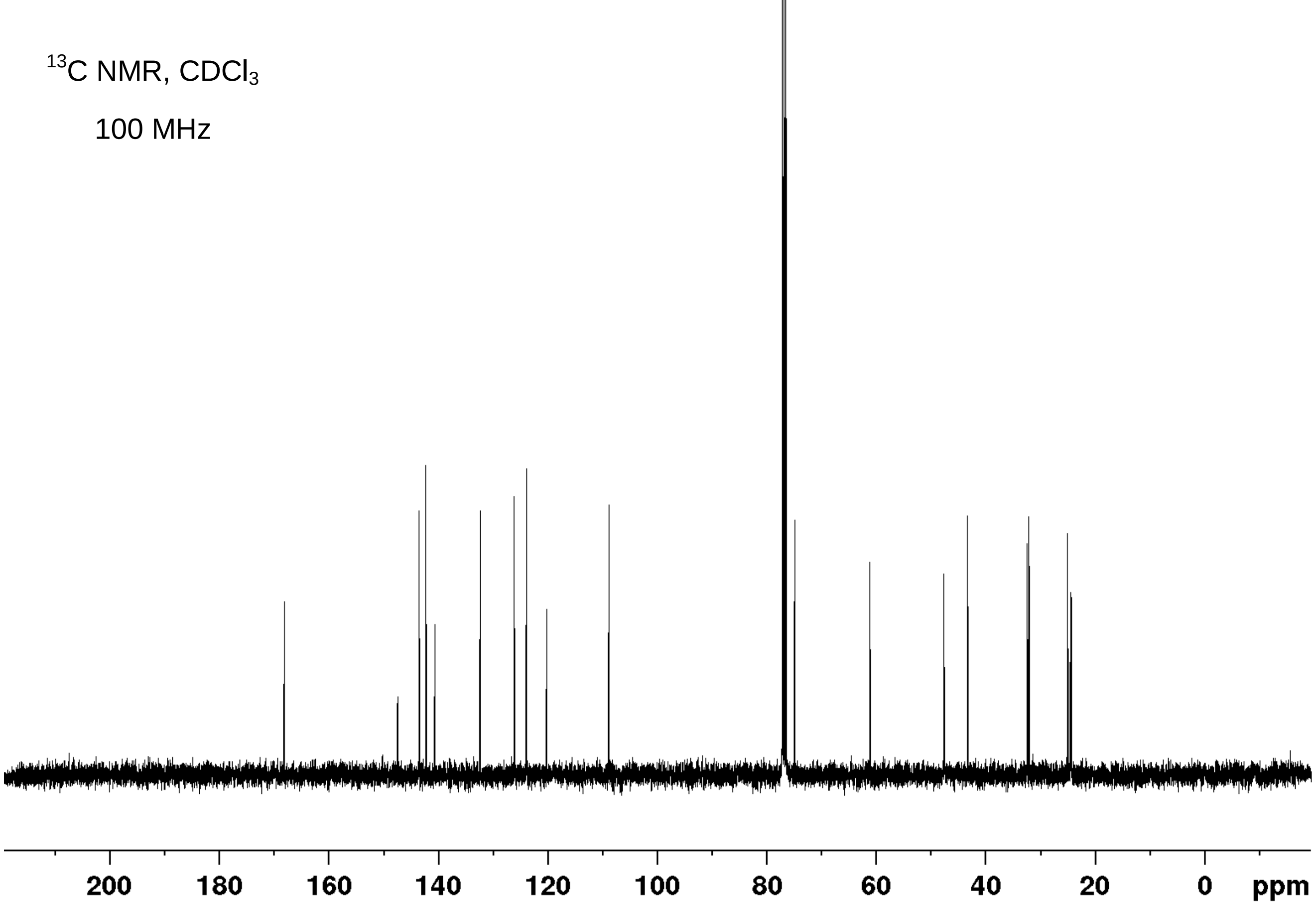


```
Current Data Parameters
NAME              MM-2-72
EXPNO                   2
PROCNO                  1

F2 - Acquisition Parameters
Date_            20160307
Time                21.04
INSTRUM             spect
PROBHD   5 mm PABBO BB/
PULPROG           zgpg30
TD                  65536
SOLVENT             CDCl3
NS                    128
DS                      4
SWH             24038.461 Hz
FIDRES           0.366798 Hz
AQ              1.3631488 sec
RG                 210.96
DW                 20.800 usec
DE                  10.00 usec
TE                  297.3 K
D1             2.00000000 sec
D11            0.03000000 sec
TD0                     1

======== CHANNEL f1 ========
SFO1          100.6278588 MHz
NUC1                  13C
P1                  10.00 usec
PLW1          48.20000076 W

======== CHANNEL f2 ========
SFO2          400.1516006 MHz
NUC2                   1H
CPDPRG[2          waltz16
PCPD2               90.00 usec
PLW2           9.80000019 W
PLW12          0.27221999 W
PLW13          0.22050001 W

F2 - Processing parameters
SI                  32768
SF            100.6178041 MHz
WDW                    EM
SSB      0
LB                   1.00 Hz
GB       0
PC                   1.40
```

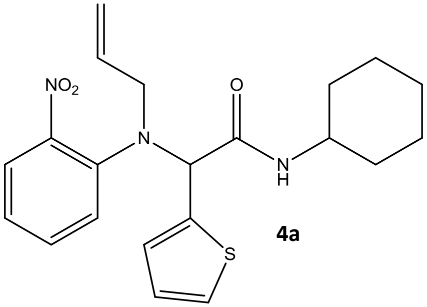

4a

^{1}H NMR, $CDCl_3$

400 MHz

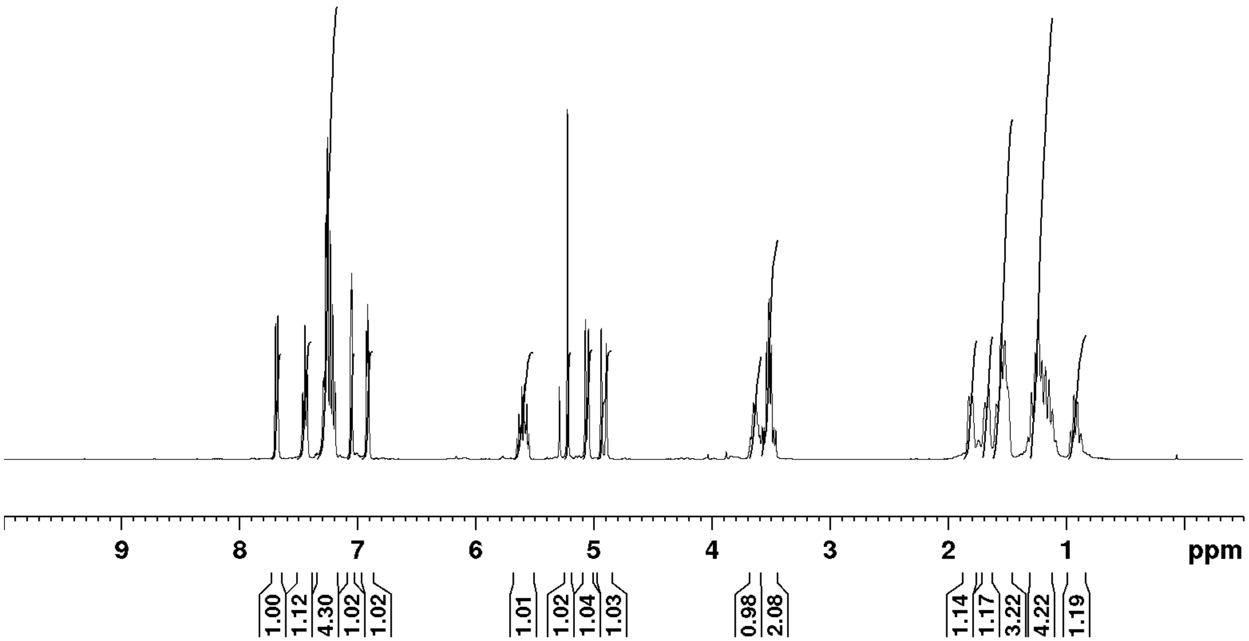


```
Current Data Parameters
NAME       MM-2-26-2-F14-21
EXPNO                    10
PROCNO                    1

F2 - Acquisition Parameters
Date_            20150512
Time                13.38
INSTRUM             spect
PROBHD    5 mm PABBO BB/
PULPROG              zg30
TD                  65536
SOLVENT             CDCl3
NS                     16
DS                      2
SWH             8012.820 Hz
FIDRES          0.122266 Hz
AQ             4.0894465 sec
RG                  82.91
DW                 62.400 usec
DE                   6.50 usec
TE                  292.2 K
D1             1.00000000 sec
TD0                     1

======== CHANNEL f1 ========
SFO1         400.1524711 MHz
NUC1                  1H
P1                 15.00 usec
PLW1          9.80000019 W

F2 - Processing parameters
SI                  65536
SF           400.1500108 MHz
WDW                    EM
SSB       0
LB                   0.30 Hz
GB        0
PC                   1.00
```

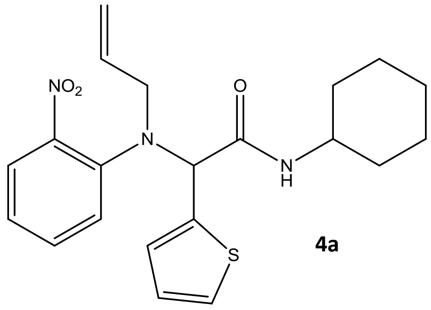

4a

^{13}C NMR, $CDCl_3$

100 MHz

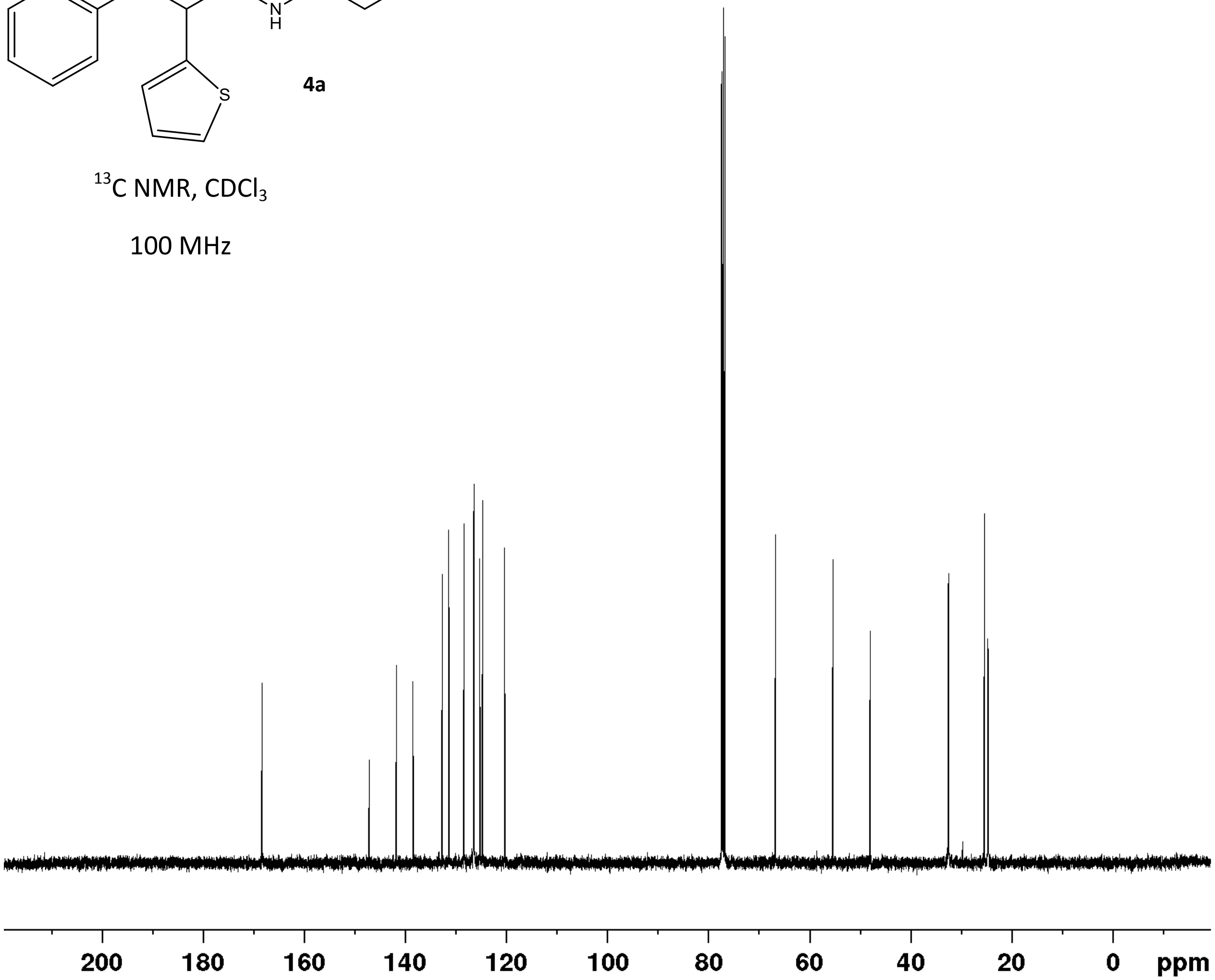


```
Current Data Parameters
NAME      MM-2-26-2-F14-21
EXPNO                    2
PROCNO                   1

F2 - Acquisition Parameters
Date_             20150904
Time                 17.15
INSTRUM              spect
PROBHD    5 mm PABBO BB/
PULPROG             zgpg30
TD                   65536
SOLVENT              CDCl3
NS                     256
DS                       4
SWH              24038.461 Hz
FIDRES            0.366798 Hz
AQ               1.3631488 sec
RG                  210.96
DW                  20.800 usec
DE                   10.00 usec
TE                   294.5 K
D1              2.00000000 sec
D11             0.03000000 sec
TD0                      1

======== CHANNEL f1 ========
SFO1           100.6278588 MHz
NUC1                   13C
P1                   10.00 usec
PLW1           48.20000076 W

======== CHANNEL f2 ========
SFO2           400.1516006 MHz
NUC2                    1H
CPDPRG[2           waltz16
PCPD2                90.00 usec
PLW2            9.80000019 W
PLW12           0.27221999 W
PLW13           0.22050001 W

F2 - Processing parameters
SI                   32768
SF             100.6177926 MHz
WDW                     EM
SSB       0
LB                    1.00 Hz
GB        0
PC                    1.40
```

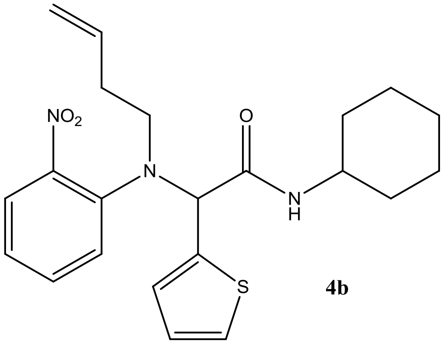

4b

^{1}H NMR, $CDCl_3$

400 MHz

```
Current Data Parameters
NAME      AF 1-10-1 R15-34
EXPNO                    2
PROCNO                   1

F2 - Acquisition Parameters
Date_             20160527
Time                 13.16
INSTRUM              spect
PROBHD    5 mm PABBO BB/
PULPROG               zg30
TD                   65536
SOLVENT              CDCl3
NS                      16
DS                       2
SWH              8012.820 Hz
FIDRES           0.122266 Hz
AQ              4.0894465 sec
RG                   66.61
DW                  62.400 usec
DE                    6.50 usec
TE                   294.0 K
D1              1.00000000 sec
TD0                      1

======== CHANNEL f1 ========
SFO1          400.1524711 MHz
NUC1                    1H
P1                   15.00 usec
PLW1            9.80000019 W

F2 - Processing parameters
SI                   65536
SF            400.1500102 MHz
WDW                     EM
SSB      0
LB                    0.30 Hz
GB       0
PC                    1.00
```

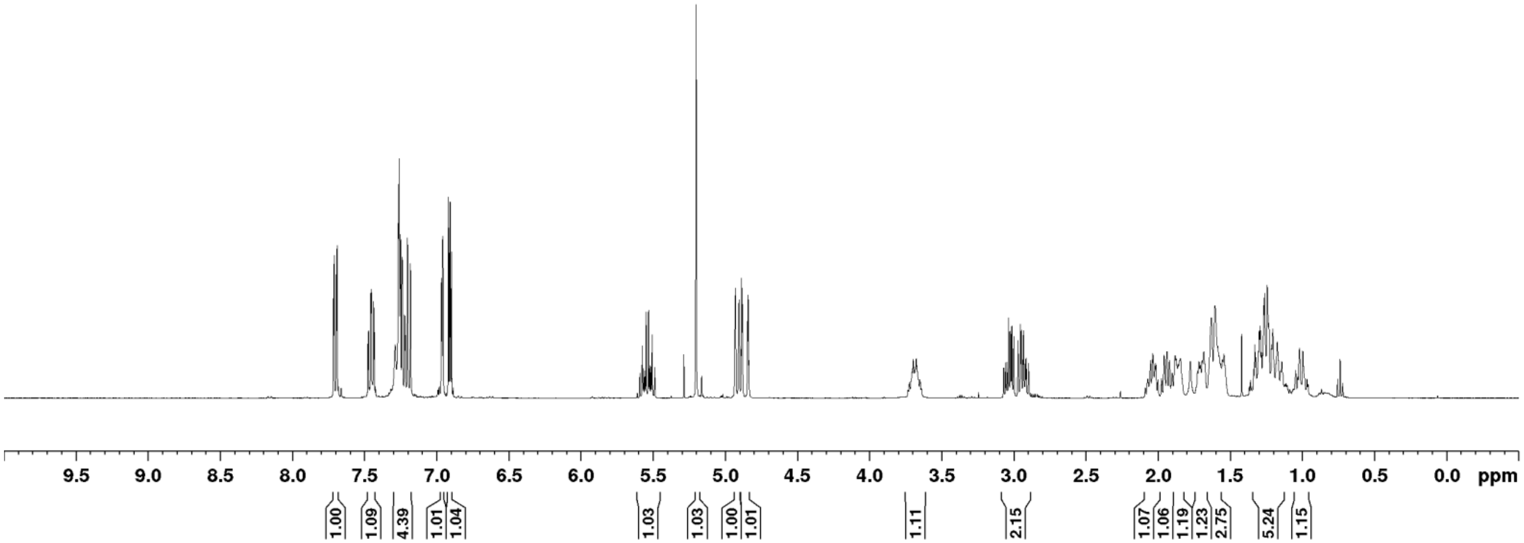

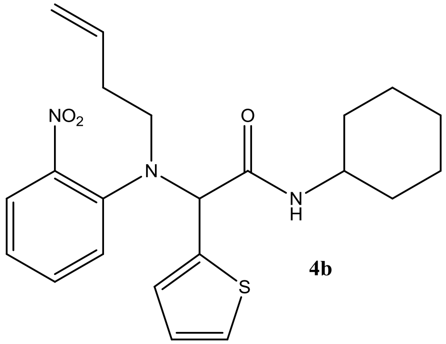

4b

^{13}C NMR, $CDCl_3$

100 MHz

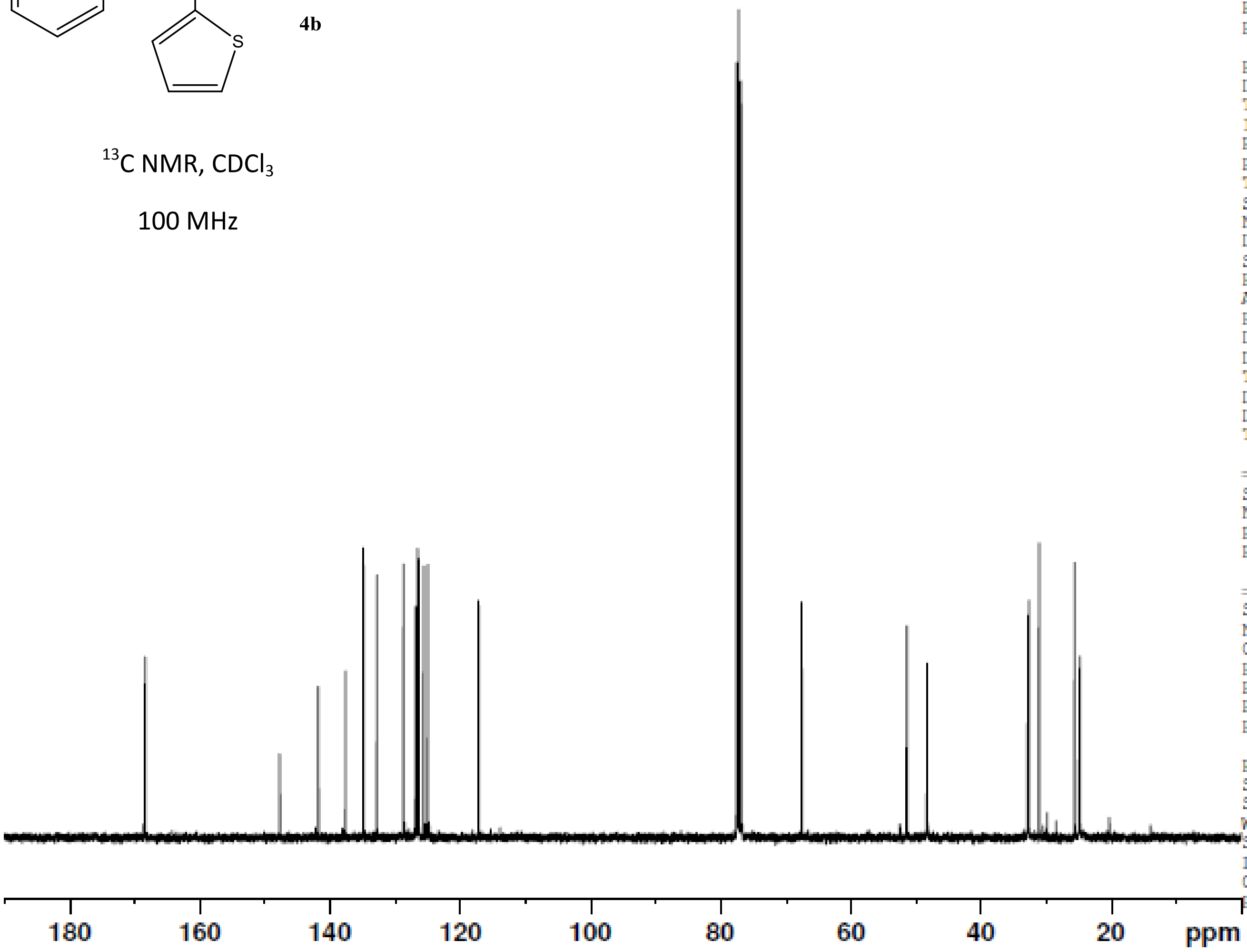

BRUKER

Current Data Parameters
NAME AF 1-10-1 pure pdct
EXPNO 1
PROCNO 1

F2 - Acquisition Parameters
Date_ 20160529
Time 13.33
INSTRUM spect
PROBHD 5 mm PABBO BB/
PULPROG zgpg30
TD 65536
SOLVENT CDCl3
NS 512
DS 4
SWH 24038.461 Hz
FIDRES 0.366798 Hz
AQ 1.3631488 sec
RG 210.96
DW 20.800 usec
DE 10.00 usec
TE 295.5 K
D1 2.00000000 sec
D11 0.03000000 sec
TD0 1

======= CHANNEL f1 =======
SFO1 100.6278588 MHz
NUC1 13C
P1 10.00 usec
PLW1 48.20000076 W

======= CHANNEL f2 =======
SFO2 400.1516006 MHz
NUC2 1H
CPDPRG[2 waltz16
PCPD2 90.00 usec
PLW2 9.80000019 W
PLW12 0.27221999 W
PLW13 0.22050001 W

F2 - Processing parameters
SI 32768
SF 100.6177865 MHz
WDW EM
SSB 0
LB 1.00 Hz
GB 0
PC 1.40
